# Lithocholic acid inhibits dendritic cell activation by reducing intracellular glutathione via TGR5 signaling

**DOI:** 10.7150/ijbs.71287

**Published:** 2022-07-11

**Authors:** Jianping Hu, Yiting Zhang, Shenglan Yi, Chaokui Wang, Xinyue Huang, Su Pan, Jinglu Yang, Gangxiang Yuan, Sisi Tan, Hong Li

**Affiliations:** The First Affiliated Hospital of Chongqing Medical University, Chongqing Key Laboratory of Ophthalmology, Chongqing Eye Institute, and Chongqing Branch of National Clinical Research Center for Ocular Diseases, Chongqing, P. R. China.

**Keywords:** Dendritic cells, Bile acids, TGR5, Uveitis, Autoimmune disease

## Abstract

Dendritic cells (DCs) are the major antigen-presenting cells and play an important role in autoimmune uveitis. Emerging evidence suggests that bile acids (BAs) regulate DCs maturation. However, the underlying mechanisms by which BAs regulate the function of DCs still need to be clarified. Here, we demonstrate that lithocholic acid (LCA) inhibits the production of pro-inflammatory cytokines and the expression of surface molecules in bone marrow-derived dendritic cells (BMDCs). LCA attenuates the severity of EAU by modulating the maturation of splenic CD11C^+^MHCII^high^ DCs. Notably, Takeda G-protein coupled receptor 5 (TGR5) deficiency partially reverses the inhibitory effect of LCA on DCs *in vitro* and *in vivo*. TGR5 activation also downregulates the NF-κB and MAPK pathways by inhibiting glutathione production and inducing oxidative stress in DCs, which leads to apoptosis and autophagy in DCs. In addition, LCA or INT-777 treatment increases the TGR5 expression in monocyte-derived dendritic cells (MD-DCs) of patients with active BD, whereas both LCA and TGR5 agonists inhibit the activation of MD-DCs. These results suggest that LCA and TGR5 agonists might be potential therapeutic drugs for the treatment of autoimmune uveitis.

## Introduction

Uveitis is an autoimmune inflammatory disease marked by inflammation of intraocular entities 1. Vogt-Koyanagi-Harada (VKH) syndrome and Behcet's disease (BD) are the most common forms of uveitis in China 2. Experimental autoimmune uveoretinitis (EAU) is the most widely used animal model of autoimmune uveitis and is characterized by autoimmune intraocular inflammation mediated by CD4+ T helper (Th) cells 2,3. The maturation and activation of antigen-presenting cells (APCs), such as dendritic cells (DCs) and macrophages, are crucial in the pathogenesis of uveitis. These cells promote the differentiation of CD4+ T cells, whereas DCs further participate in the maintenance of peripheral immune tolerance 4-6.

Primary bile acids (BAs) are produced in the liver and converted to secondary BAs by intestinal bacteria 7. BAs are generally considered to play key roles in host metabolism and energy balance via several nuclear receptors and/or G-protein-coupled receptors 7. Previous studies indicated that BAs are involved in the maintenance of innate immune responses via the aforementioned receptors 8. Recent studies suggest that BAs also play important roles in the adaptive immune response and modulate the secretion of IL-12 in DCs, inhibiting the differentiation of Th17 cells but promoting that of Treg cells 9,10. Additionally, gut microbiota-related dysmetabolism of BAs has been implicated in the development of several autoimmune and inflammatory diseases, such as type 1 diabetes (T1D), inflammatory bowel disease (IBD) and multiple sclerosis (MS) 11-13.

Takeda G protein-coupled receptor 5 (TGR5) is a membrane receptor activated by BAs, deoxycholic acid (DCA) and lithocholic acid (LCA) are preferential agonists 7,14,15. However, the most potent ligand for farnesoid X receptor (FXR, a nuclear receptor) is chenodeoxycholic acid (CDCA) in humans 7,14,15. BAs stimulate TGR5 signaling to regulate several physiological pathways related to metabolic homeostasis, including the improvement of glucose tolerance and enhancement of energy expenditure 16-18. Additionally, it has been reported that the activation of TGR5 in monocytes and macrophages can reduce the production of pro-inflammatory cytokines and inhibit the expression of co-stimulatory molecules, suggesting that BAs play an immunomodulatory role through TGR5 8,19.

Glutathione (GSH) regulates several metabolic and cell cycle-dependent functions, such as scavenging of exogenous toxins and free radicals, protein synthesis, and maintenance of intracellular redox balance 20. In addition, GSH regulates the proliferation of lymphocytes, the function of polymorphonuclear cells, and the secretion of several cytokines and chemokines 21. Intracellular GSH levels in APCs influence the production of cytokines such as IL-12, IL-1β and IL-23 22,23. Severe GSH depletion in APCs also inhibits the proliferation and differentiation of CD4+ T cells 24.

In the present study, we found that BAs regulate the secretion of cytokines in DCs and their subsequent antigen presentation ability via TGR5 signaling. In addition, TGR5 activation promotes apoptosis and autophagy in DCs by inhibiting intracellular GSH production.

## Materials and methods

### Mouse model

TGR5^-/-^ mice were obtained from Viewsolid Biotech (Beijing, China). TGR5^+/+^ (C57BL/6J) mice were purchased from Jackson Laboratory (Bar Harbor, ME, USA). EAU was induced in 6- to 8-week-old female mice through subcutaneous injection of 300 µg human IRBP_651-670_ dissolved in 0.2 ml emulsion 1:1 (vol/vol) supplemented with CFA containing 2.5 mg/mL* Mycobacterium tuberculosis* strain H37Ra (BD Biosciences, USA). The mice also received intraperitoneal injections of 0.5 µg* Bordetella pertussis* toxin (Sigma-Aldrich, MO, USA) suspended in 0.1 ml PBS. The toxin was injected once on the first day and after 2 days. Then, EAU mice were fed a special LCA diet (0.01%) or normal diet. Clinical manifestations and histopathological changes in the mice were evaluated as previously described 1.

### Human study

Healthy individuals (n=16), patients with active BD (n=8) and patients with inactive BD (n=8) were enrolled in this study. BD diagnosis was performed according to the international nomenclature committee guidelines 25. All patients were first-time visitors to our hospital and did not receive any treatment prior to enrollment and extraction of samples.

### Real-Time PCR

TRIzol (Invitrogen, CA, USA) was used to extract the total RNA from Bone marrow dendritic cells (BMDCs), retinal tissues, and monocyte-derived dendritic cells (MD-DCs). The PrimeScript RT reagent kit (Takara, Dalian, China) was used to synthesize cDNA from the RNA. The resultant DNA was amplified by real-time PCR as previously reported 1. The sequences of the primers used in this study are shown in Table S2.

### Cell culture

BMDCs from the mice were extracted and cultured as previously described 26. Briefly, 5×10^5^ BMDCs were stimulated using lipopolysaccharides (LPS) (1 μg/mL, Sigma-Aldrich) and bile acids (10 µM, Sigma-Aldrich) or INT-747(100 µM, MedChem Express, USA) or INT-777 (100 µM, MedChem Express) for 24 h.

Mice CD4+ T cells were isolated using mouse CD4 microbeads (Miltenyi Biotec, Bergisch Gladbach, Germany) and cocultured with BMDCs pretreated with LCA or INT-777 at a ratio of 5:1 (CD4+ T cells: BMDCs) for 5 days.

Human CD14+ monocytes in PBMCs were isolated using Human CD14 microbeads (Miltenyi Biotec). CD14+ monocytes were incubated with 50 ng/mL IL-4 (AcroBiosystems, Newark, NJ, USA) and 100 ng/mL GM-CSF (AcroBiosystems) to induce DC maturation. 7 days later, 5×10^5^ MD-DCs were treated with 100 ng/ml LPS and 10 µM LCA or 100 µM INT-777 for 24 h.

### Inhibition of TGR5 and FXR expression

To explore the role of TGR5 and FXR in BMDCs, the cells were transfected with TGR5-siRNAs or FXR-siRNAs using Lipofectamine 3000 (Invitrogen, CA, USA) in accordance with the manufacturer's protocol. An adenovirus encoding human TGR5-siRNA was constructed (Hanbio, Shanghai, China) and transfected into MD-DCs as previously reported 5.

### Evaluation of blood-retinal barrier (BRB) integrity

The integrity of the BRB was evaluated using Evans blue (Sigma-Aldrich, MO, USA) as previously described 1. The flat-placed retina was observed using a fluorescence microscope.

### Flow cytometry

Anti-mouse IL-17A-PE, CD4-APC and IFN-γ-PE-cy7, anti-mouse IgG isotype, anti-human IFN-γ-PE-cy7, CD4-APC and IL-17A-PE and anti-human IgG isotype were obtained from eBioscience. For intracellular expression of IFN-γ and IL-17, CD4+ T cells were incubated for 1 hours with ionomycin (1 μg/mL), PMA (50 ng/mL) and for another 4 hours with brefeldin A (10 μg/ml, Sigma-Aldrich), harvested, washed and fixed before permeabilization.

BMDCs or MD-DCs were stained for 30 minutes at 4 °C with anti-mouse CD86-FITC (eBioscience), CD11c-APC (eBioscience), CD80-PE (eBioscience), CD40-FITC (BioLegend, San Diego, CA, USA), MHCII-PE (eBioscience), anti-human CD86-PE-cy7 (BioLegend), CD40-FITC (BioLegend), HLA-DR-PE-cy5.5 (BioLegend), and CD80-PE (BioLegend).

### Enzyme-linked immunosorbent assay (ELISA)

The concentrations of human IFN-γ, IL-1β, IL-17, IL-6 and TNF-α as well as mouse IL-1β, IL-12/p70, IL-6, IL-23, IL-17, IFN-γ and TNF-α in the supernatants of BMDCs, MD-DCs or mouse serum were analyzed using Duoset ELISA kits (R&D Systems, MN, USA). The concentration of human IL-12/p70 in the supernatants of MD-DCs was assessed using an ELISA kit (Thermo Fisher Scientific).

### RNA-seq and screening of DEGs between TGR5^-/-^ and TGR5^+/+^ BMDCs

RNA from BMDCs of TGR5^-/-^ and TGR5^+/+^ mice treated with LCA was sequenced. The technique was performed using the Illumina NovaSeq 6000 platform. DEGs were identified using DESeq2. P values were adjusted to control for the false discovery rate using Benjamini and Hochberg's method. DEGs with an adjusted P value <0.05 and |log2foldchange|>1 were considered statistically significant**.**

### GO terms and KEGG pathway enrichment analysis

The GO database (http://www.geneontology.org) provides functional classification, including categories of cellular component (CC), molecular function (MF) and biological processes (BP). Enriched pathways were evaluated by the KEGG database (http://www.kegg.jp/kegg/pathway.html). GO terms and KEGG pathway enrichment analyses were performed on DEGs using the DAVID database. FDR<0.05 was considered significant.

### Examination of the activity of antioxidant enzymes and reactive oxygen species (ROS)

The production of intracellular ROS in cultured cells was assessed using 2′,7′-dichlorofluorescein diacetate (DCFH-DA, Sigma-Aldrich). Before LPS stimulation, the cells were pretreated with DCFH-DA for 20 min. The fluorescence DCFH-DA signal was analyzed using flow cytometry. Briefly, frozen sections of eyeball tissue were stained with dihydroethidium (DHE, Servicebio, Wuhan, China) at 4 °C for 30 min before being observed under a fluorescence microscope.

Cell extracts of BMDCs and MD-DCs were collected to detect the concentrations of GPx (Cayman), CAT (Cayman) and GSH (Cayman) using the appropriate assay kits according to the manufacturer's instructions. Intracellular GSH levels in the retinal tissues of EAU mice were quantified using monochlorobimane (MCB, MedChem Express, USA). Frozen sections of eyeballs were stained with MCB for 30 min, rinsed and observed under a fluorescence microscope.

### Western blot

The expression of proteins of interest in BMDCs was analyzed using western blotting as previously described 1. The following primary antibodies were used: anti-TGR5 (Abcam, Cambridge, MA, USA), FXR (Abcam), Bax (Abcam), BCL-2 (Abcam), caspase3 (Abcam), cleaved-caspase3 (Abcam), LC3 (Abcam), Belin1(Abcam), P62 (Abcam), P65 (Abcam), Ikbα (Abcam), ERK1/2 (Santa Cruz), P38 (Abcam), JNK (Abcam), phospho-P65 (CST), phospho-Ikbα (Abcam), phospho-ERK1/2 (Santa Cruz), phospho-P38 (Abcam), phospho-JNK (Abcam), and β-actin (Abcam).

### Ethics statement

The protocols of all studies were approved by the ethics committee of our hospital (2018-049). All participants agreed to participate in this study and gave informed written consent. All experiments involving human participants strictly followed the principles of the Helsinki Declaration. The animal protocols were approved by the Animal Care and Use Committee of our hospital and were conducted in line with the ARVO Use of Animals in Ophthalmic and Vision Research guidelines.

### Statistical analysis

Differences between groups were analyzed using one-way ANOVA, whereas Dunn's correction analysis was used for multiple groups. Differences in independent groups were analyzed using unpaired Student's t test and the Mann-Whitney U test. Comparisons between paired groups were performed using the t test and the Wilcoxon test. Continuous data are expressed as the mean ± standard deviation (SD). Statistical significance was set at *p<0.05 or **p<0.01.

## Results

### Bile acids inhibit the production of pro-inflammatory cytokines and the expression of surface markers on DCs

**Figure 1:**
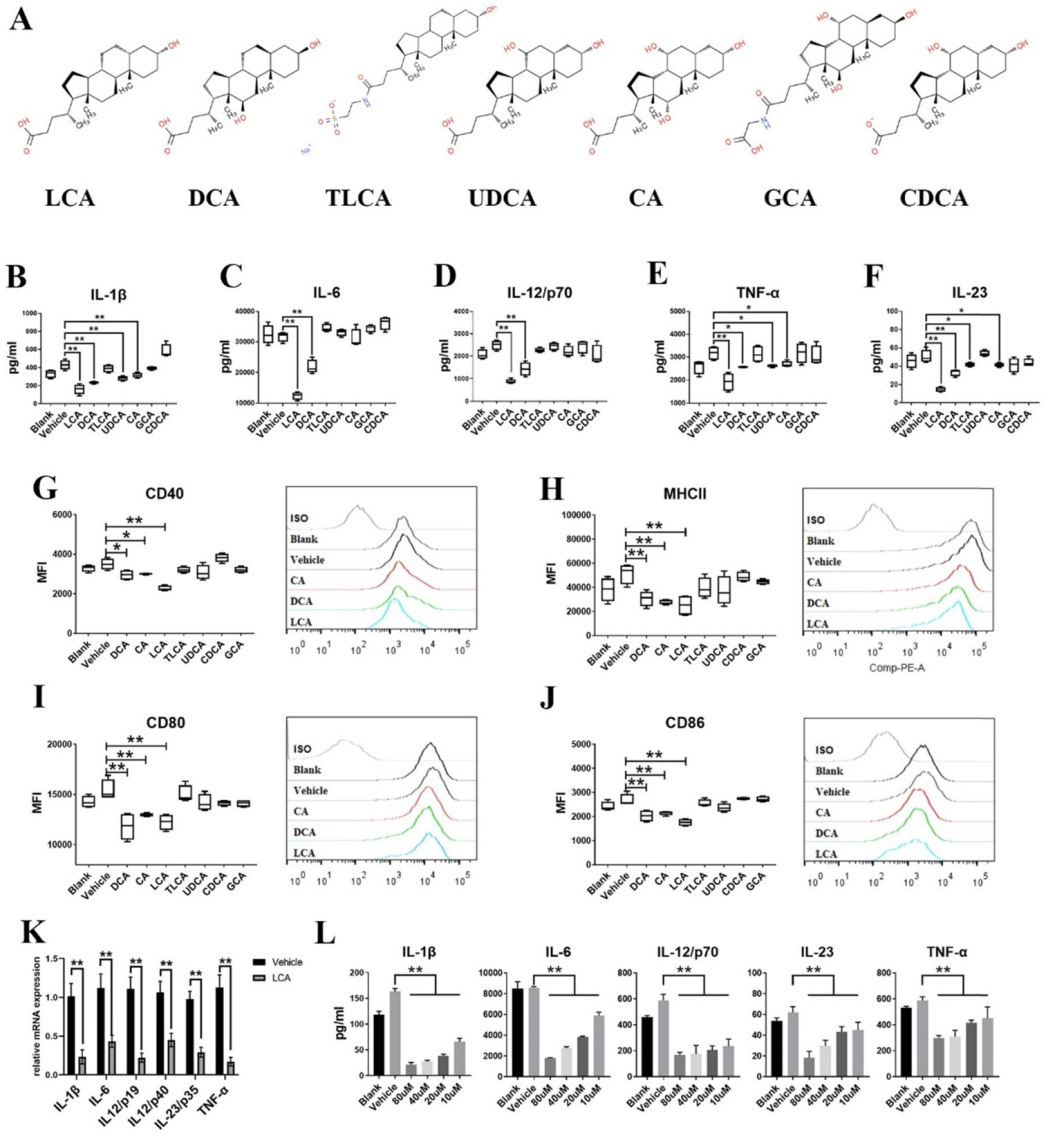
** Bile acids inhibit the secretion of pro-inflammatory cytokines and the expression of surface markers on dendritic cells. A.** The structures of bile acids. **B-F.** The expression of IL-1β, IL-6, IL-12/p70, IL-23 and TNF-α in BMDCs primed with LPS and thereafter treated with selected BAs (10 µM) was tested by ELISA (n=4 per group). **G-J.** The expression of CD40, CD80, CD86 and MHCII in BMDCs primed with LPS and thereafter treated with selected BAs (10 µM) was tested by Flow cytometry (10 µM; n=4 per group). **K.** The mRNA expression of IL-1β, IL-6, IL-12/p19, IL-12/p40, IL-23/p35 and TNF-α in BMDCs primed with LPS and thereafter treated with LCA (10 µM) was assayed by RT-qPCR (n=4 per group). **L.** The expression of IL-1β, IL-6, IL-12/p70, IL-23 and TNF-α in BMDCs primed with LPS and thereafter treated with LCA at doses of 80 µM, 40 µM, 20 µM and 10 µM was assayed by ELISA (n=4 per group). Data are shown as mean ± SD. ns p>0.05, *p<0.05, **p<0.01.

To study the effects of different BAs on DCs, LPS-primed BMDCs were treated with CDCA, LCA, DCA, taurolithocholic acid (TLCA), ursodeoxycholic acid (UDCA), cholic acid (CA) or glycocholic acid (GCA) (Figure 1A). The results showed that LCA, DCA, CA, TLCA and UDCA inhibited the secretion of several pro-inflammatory cytokines, including IL-12/p70, IL-1β, IL-23, IL-6 and TNF-α (Figures 1B-F). However, DCA, LCA and CA further, but only slightly, inhibited the expression of the surface markers CD40, CD86, CD80 and MHCII in BMDCs (Figures 1G-J). DCA and LCA inhibited the expression of all the aforementioned pro-inflammatory cytokines and surface markers, with LCA exerting the greatest effect. Therefore, LCA was used in most of the subsequent experiments. LCA inhibited the mRNA expression of IL-12/p19, IL-12/p40, IL-6, IL-1β, IL-23/p35 and TNF-α in BMDCs (Figure 1K). We further found that the inhibition of pro-inflammatory cytokines secretion by LCA occurred in a dose-dependent manner (Figure 1L). Interestingly, the LCA dosage required to inhibit cytokines secretion was comparable to that of BAs in peripheral circulation (2-10 μM) 27. BAs affect anti-inflammatory functions by upregulating the expression of IL-10, which is involved in the activation of CREB 28. Notably, LCA had no effect on IL-10 expression or CREB activation in BMDCs (Figures S1A-C).

### LCA inhibits the secretion of pro-inflammatory cytokines in DCs via TGR5 signaling

**Figure 2:**
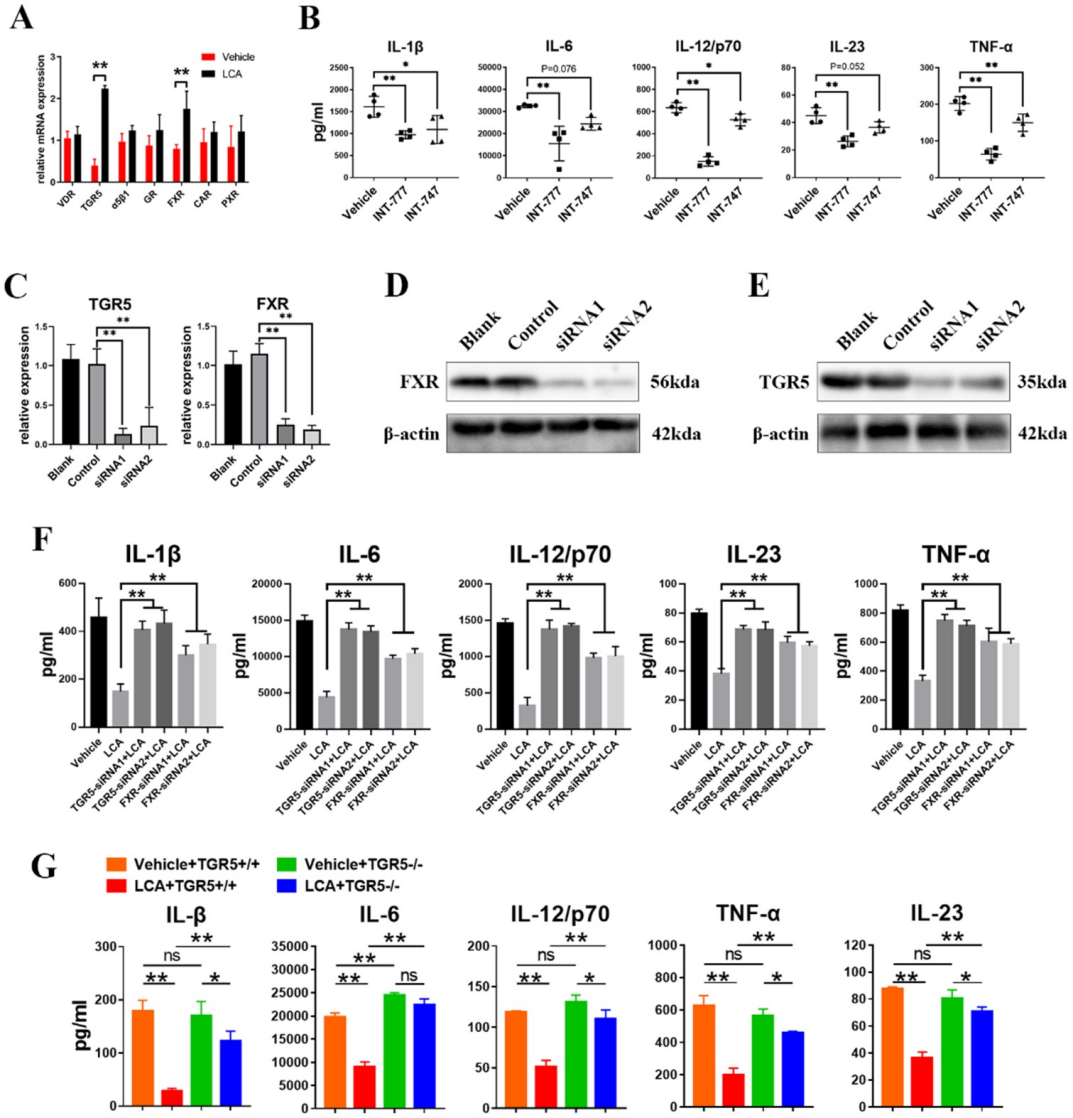
** LCA inhibits the secretion of pro-inflammatory cytokines in DCs via TGR5 signaling. A.** The mRNA expression of bile acid receptors (VDR, TGR5, α5β1, GR, CAR, PXR, FXR) in BMDCs primed with LPS thereafter treated with LCA was tested by RT-qPCR (n=4 per group). **B.** The secretion of IL-1β, IL-6, IL-12/p70, IL-23 and TNF-α by BMDCs primed with LPS thereafter treated with INT-777 or INT-747 was assayed by ELISA (n=4 per group). **C.** The mRNA expression of TGR5 or FXR in BMDCs respectively treated with TGR5-siRNAs or FXR-siRNAs was tested by RT-qPCR (n=4 per group). **D.** The expression of TGR5 in BMDCs treated with TGR5-siRNAs was tested by Western blot. **E.** The expression of FXR in BMDCs treated with FXR-siRNAs was tested by Western blot. **F.** TGR5 or FXR expression in the BMDCs was inhibited using corresponding siRNAs. The secretion of IL-1β, IL-6, IL-12/p70, IL-23 and TNF-α by BMDCs primed with LPS and stimulated with LCA was tested by ELISA. (n=4 per group). **G.** The expression of IL-1β, IL-6, IL-12/p70, IL-23 and TNF-α in TGR5^+/+^ and TGR5^-/-^ BMDCs primed with LPS and thereafter treated with LCA was assayed by ELISA (n=4 per group). Data are shown as mean ± SD. ns p>0.05, *p<0.05, **p<0.01.

BAs can activate several receptors, including TGR5, FXR, vitamin D receptor (VDR), glucocorticoid receptor (GR), α5β1, pregnane X receptor (PXR) and constitutive androstane receptor (CAR) 7. The results showed that LCA induced the mRNA expression of FXR and TGR5 in BMDCs (Figure 2A). In addition, INT-777 (a TGR5-specific agonist) significantly inhibited the secretion of IL-6, IL-1β, IL-23, IL-12/p70 and TNF-α in BMDCs (Figure 2B). However, INT-747 (an FXR-specific agonist) only reduced the expression of TNF-α, IL-1β and IL-12/p70 (Figure 2B). Then, the expression of TGR5 and FXR in BMDCs was disrupted using siRNAs (Figures 2C-E). We found that inhibition of TGR5 expression reversed the inhibitory effect of LCA on the secretion of cytokines on a greater scale than FXR (Figure 2F). *In vitro* studies using TGR5^-/-^ BMDCs found a similar result (Figure 2G). These findings suggest that LCA regulates the function of DCs via TGR5 signaling.

### LCA attenuates the severity of EAU in mice through TGR5 signaling

**Figure 3:**
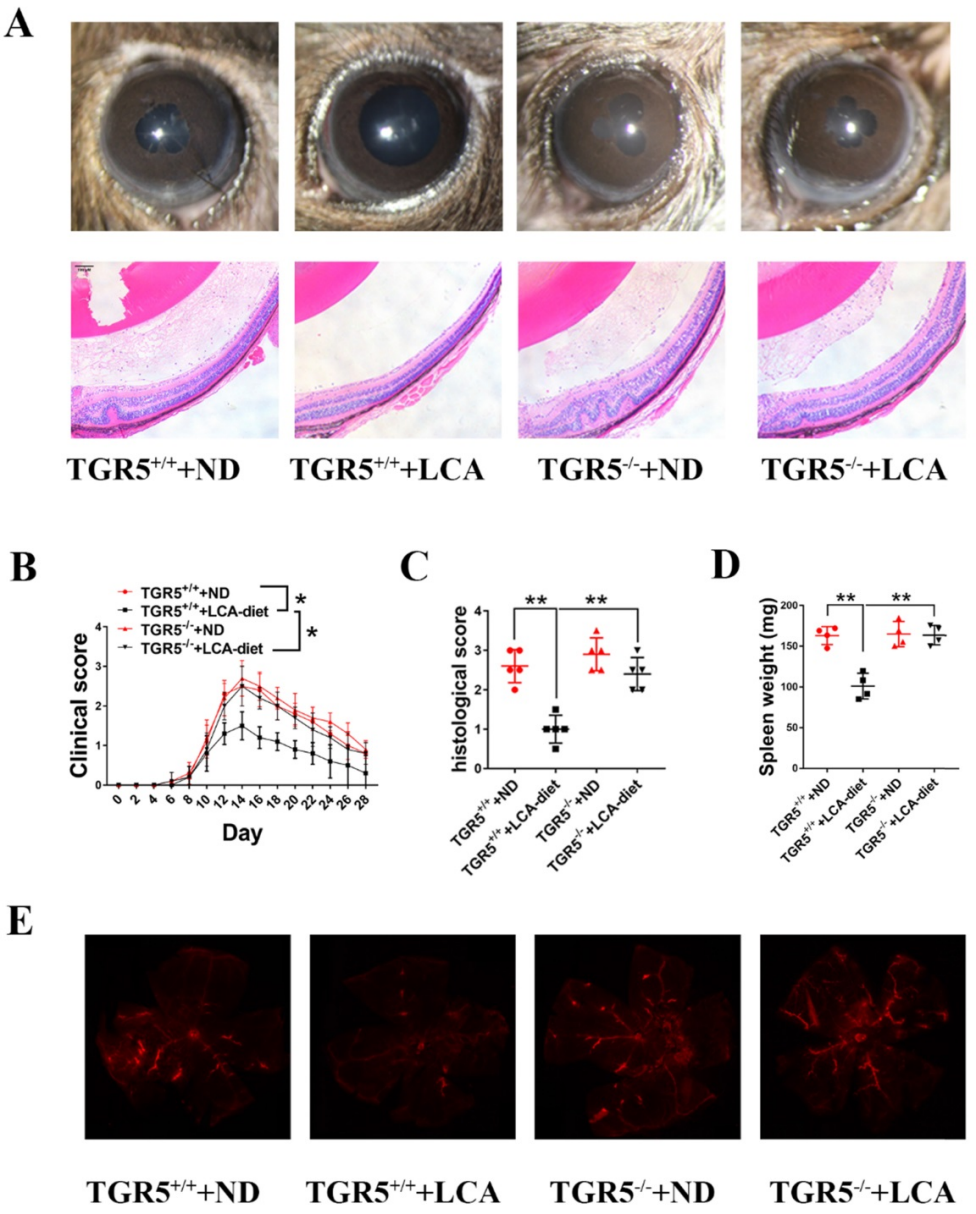
** LCA attenuates the severity of EAU in mice via TGR5 signaling.** TGR5^+/+^ mice and TGR5^-/-^ mice were injected with IRBP_651-670_ and CFA and fed on LCA-diet or ND (n= 4-6 per group). **A.** Slit-lamp and hematoxylin and eosin (H&E) staining section images of EAU eyes at the 14^th^ day after IRBP_651-670_ and CFA injection. **B.** The clinical scores were measured every two days after inducing EAU (p value was evaluated at 14^th^ day after immunization, *p<0.05). **C.** The histological scores were assessed by hematoxylin and eosin (H&E) staining paraffin-embedded sections at the 14^th^ day after IRBP_651-670_ and CFA injection. **D.** The weight of spleen of the EAU mice at the 14^th^ day after IRBP_651-670_ and CFA injection. E. Representative Evans-blue images of retina tissue sections of EAU mice at the 14^th^ day after IRBP_651-670_ and CFA injection. Scale bar 100 µM. Data are shown as mean ± SD. ns p>0.05, *p<0.05, **p<0.01.

DCs play an important role in immune responses and have been implicated in the development of uveitis 6. We found that feeding TGR5^+/+^ mice an LCA diet significantly ameliorated the severity and slowed the progression of EAU (Figures 3A-B). However, TGR5 knockout significantly reversed this effect (Figures 3A-B). Notably, the inhibitory effect of LCA on pathological manifestations occurred in TGR5^+/+^ EAU mice but not in TGR5^-/-^ EAU mice (Figures 3A and 3C). Evans blue injection further revealed that LCA only had a protective effect on the integrity of retinal vessels in TGR5^+/+^ EAU mice (Figure 3E). LCA also decreased the expression of MCP-1 and IL-6, but increased that of IL-10 in the retinal tissues of TGR5^+/+^ EAU mice (Figure S2A).

To further explore the involvement of TGR5 signaling in DCs, we weighed the spleens of EAU mice (Figure 3D) and analyzed the proportions of splenic CD11c+MHC^high^ DCs using flow cytometry (FCM). We found that LCA diet significantly disrupted the maturation of DCs to CD11^+^MHCII^high^ subtypes in splenocytes of TGR5^+/+^ EAU mice (Figures S3A-B). Consistently, we found that the production of pro-inflammatory cytokines by splenic CD11c+ cells isolated from these mice was decreased. However, inhibition of TGR5 reversed the inhibitory effect of LCA on splenic DCs in EAU mice (Figures S3A-C). LCA had no effect on the expression of surface markers on CD11+ DCs *in vivo* (data not shown).

### LCA inhibits the antigen presentation function of DCs via TGR5 signaling

**Figure 4:**
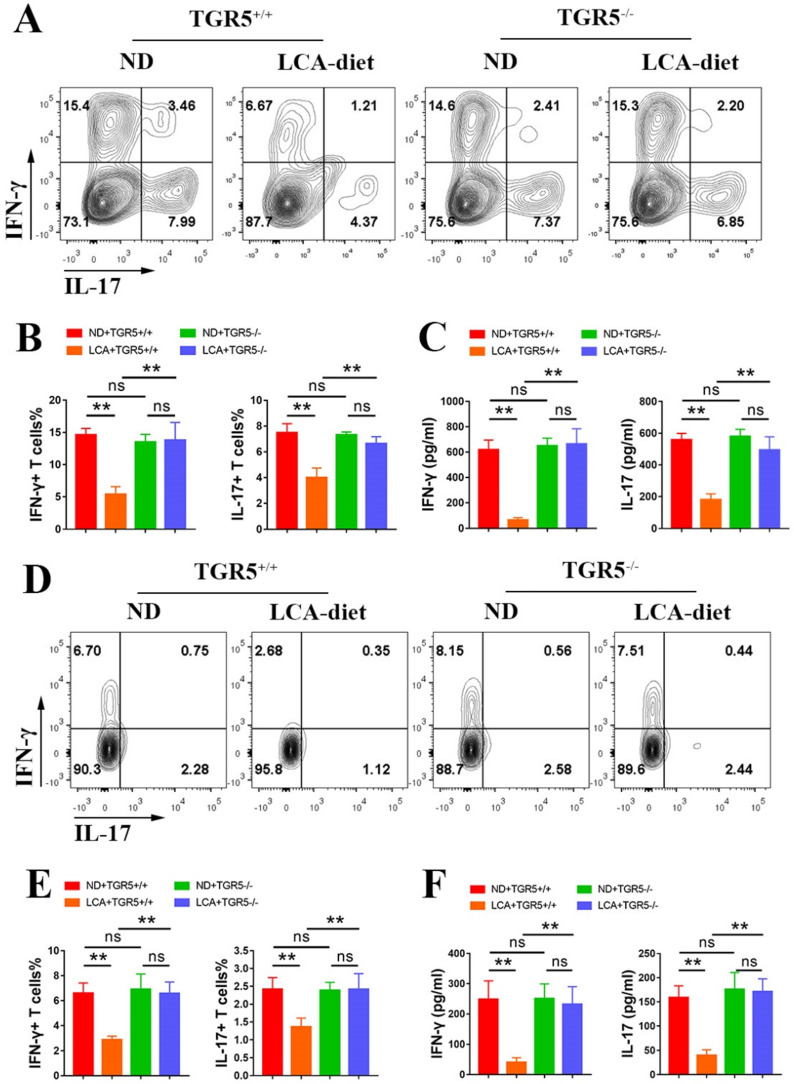
** LCA inhibits the antigen presentation function of DCs via TGR5 signaling. A-C.** CD11c+ DCs isolated from splenocytes of TGR5^+/+^ and TGR5^-/-^ EAU mice were co-cultured with naïve T cells (DCs: naïve T cells =1: 5, n=4 per group). **A and B.** The proportion of Th1 and Th17 cells was measured by Flow cytometry. **C.** The expression of IL-17 and IFN-γ in culture supernatants was tested by ELISA. **D-F.** TGR5^+/+^ mice and TGR5^-/-^ mice were injected with IRBP_651-670_ and CFA, prior to the LCA diet (n= 4-6 per group). **D and E.** The proportion of Th1 and Th17 cells in splenocytes of EAU mice was measured by Flow cytometry. F. The expression of IL-17 and IFN-γ in the serum of EAU mice was tested by ELISA. Data are shown as mean ± SD. ns p>0.05, *p<0.05, **p<0.01.

To explore the role of TGR5 signaling in the antigen-presenting ability of DCs, CD11c+ cells from TGR5^+/+^ and TGR5^-/-^ EAU mice fed either ND or LCA diet were co-cultured with naïve CD4+ T cells from TGR5^+/+^ EAU mice at a ratio of 1:5 (CD11c+ DCs: CD4+ T cells). CD11c^+^ DCs isolated from TGR5^+/+^ EAU mice fed LCA diet significantly inhibited the differentiation of Th17 and Th1 cells (Figures 4A-B) and the secretion of IFN-γ and IL-17 (Figure 4C). The proportions of Th17 and Th1 cells and the levels of IFN-γ and IL-17 were significantly higher in TGR5^-/-^ group than in TGR5^+/+^ group (Figures 4A-C).

The role of LCA on the antigen-presenting ability of BMDCs was also evaluated. TGR5^+/+^ and TGR5^-/-^ BMDCs were pretreated with LCA, primed with LPS, and co-cultured with TGR5^+/+^ naïve CD4+ T cells from EAU mice at a ratio of 1: 5 (BMDCs: CD4+ T cells). LCA-pretreated BMDCs significantly inhibited the differentiation of Th1 and Th17 cells and the production of IFN-γ and IL-17 (Figures S4A-C). However, TGR5 deficiency significantly blocked this effect (Figures S4A-C).

Further studies revealed that LCA inhibited the differentiation of Th17 and Th1 cells in the spleens and the levels of IFN-γ and IL-17 in the serum of TGR5^+/+^ EAU mice, but not in TGR5^-/-^ EAU mice (Figure 4D-F). A similar result was also observed in the retinas of EAU mice (Figure S2B-C).

### LCA inhibits the activation of human dendritic cells derived from monocytes via TGR5 signaling

**Figure 5:**
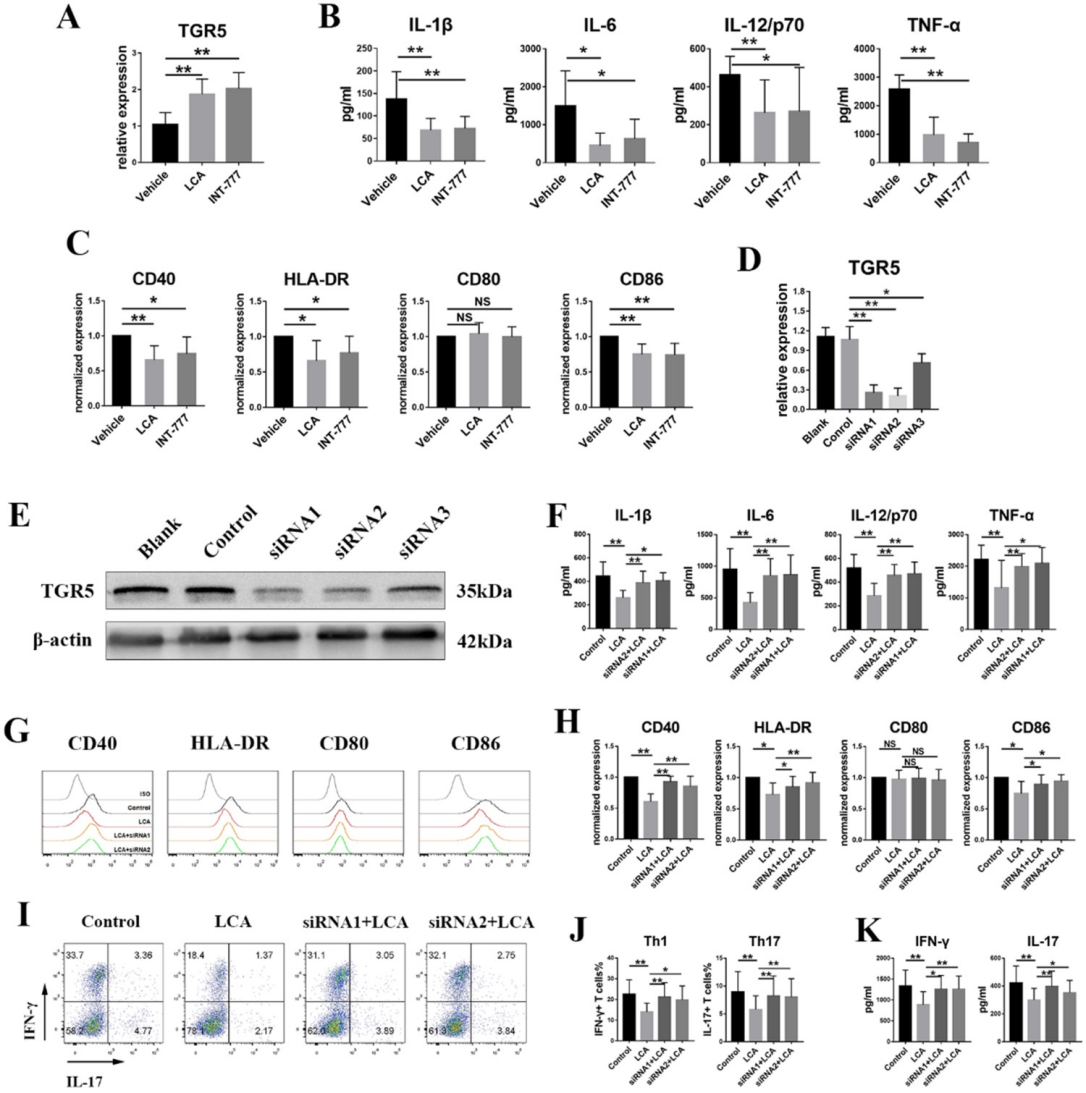
** LCA inhibits the activation of human dendritic cells derived from monocytes via TGR5 signaling. A.** The mRNA expression of TGR5 in MD-DCs primed with LPS and thereafter treated with LCA or INT-777 was tested by RT-PCR (n=6 per group). **B and C.** MD-DCs isolated from 8 active BD patients were primed with LPS and thereafter treated with LCA or INT-777. **B.** The expression of IL-1β, IL-6, IL-12/p70 and TNF-α in the cultured supernatant was tested by ELISA. **C.** The expression of CD40, CD80, CD86 and HLA-DR in MD-DCs was assayed by Flow cytometry. **D.** The expression of TGR5 mRNA in MD-DCs treated with TGR5-siRNAs was tested by RT-PCR (n=4 per group). **E.** The protein level of TGR5 in MD-DCs treated with TGR5-siRNAs was tested by Western blot. **F-H.** MD-DCs were pretreated with TGR5-siRNAs, primed with LPS and thereafter stimulated using LCA (n=6 per group). **F.** The expression of IL-1β, IL-6, IL-12/p70 and TNF-α in the cultured supernatant was tested by ELISA. **G and H.** The expression of CD40, CD80, CD86 and HLA-DR in MD-DCs was assayed by Flow cytometry. **I-K.** LCA treated MD-DCs following TGR5-siRNA were co-cultured with naïve T cells (MD-DCs: naïve T cells =1: 5, n=4 per group). **I and J.** The proportion of Th1 and Th17 cells was assayed by Flow cytometry. **K.** The level of IL-17 and IFN-γ in the cultured supernatants was tested by ELISA. Data are shown as mean ± SD. ns p>0.05, *p<0.05, **p<0.01.

According to our previous studies, active BD patients displayed downregulated TGR5 expression in MD-DCs 29. Further analyses were performed to evaluate whether LCA inhibits the activation of human DCs via TGR5 signaling. LPS-primed MD-DCs from active BD patients were also incubated with LCA or INT-777. We found that LCA and INT-777 significantly increased the mRNA expression of TGR5 (Figure 5A) but inhibited the expression of HLA-DR, CD86 and CD40 and the secretion of TNF-α, IL-6, IL-1β and IL-12/p70 (Figures 5B-C). However, LCA did not affect the expression of CD80 (Figure 5C). TGR5 expression was knocked down in MD-DCs using TGR5-siRNA (Figures 5D-E). The results showed that inhibition of TGR5 significantly decreased TGR5 mRNA expression and reversed the effect of LCA on MD-DCs (Figures 5F-H).

LCA-pretreated MD-DCs from BD patients were co-cultured with naïve CD4+ T cells from healthy individuals at a ratio of 1: 5. The FCM test revealed that LCA-pretreated MD-DCs significantly reduced the percentages of Th1 and Th17 cells (Figures 5I-J). The concentrations of IFN-γ and IL-17 in the supernatant were also decreased in LCA-pretreated group (Figure 5K). However, inhibition of TGR5 using siRNA significantly reversed this effect (Figures 5I-K).

### TGR5 signaling inhibits the functions of DCs by regulating intracellular glutathione metabolism

**Figure 6:**
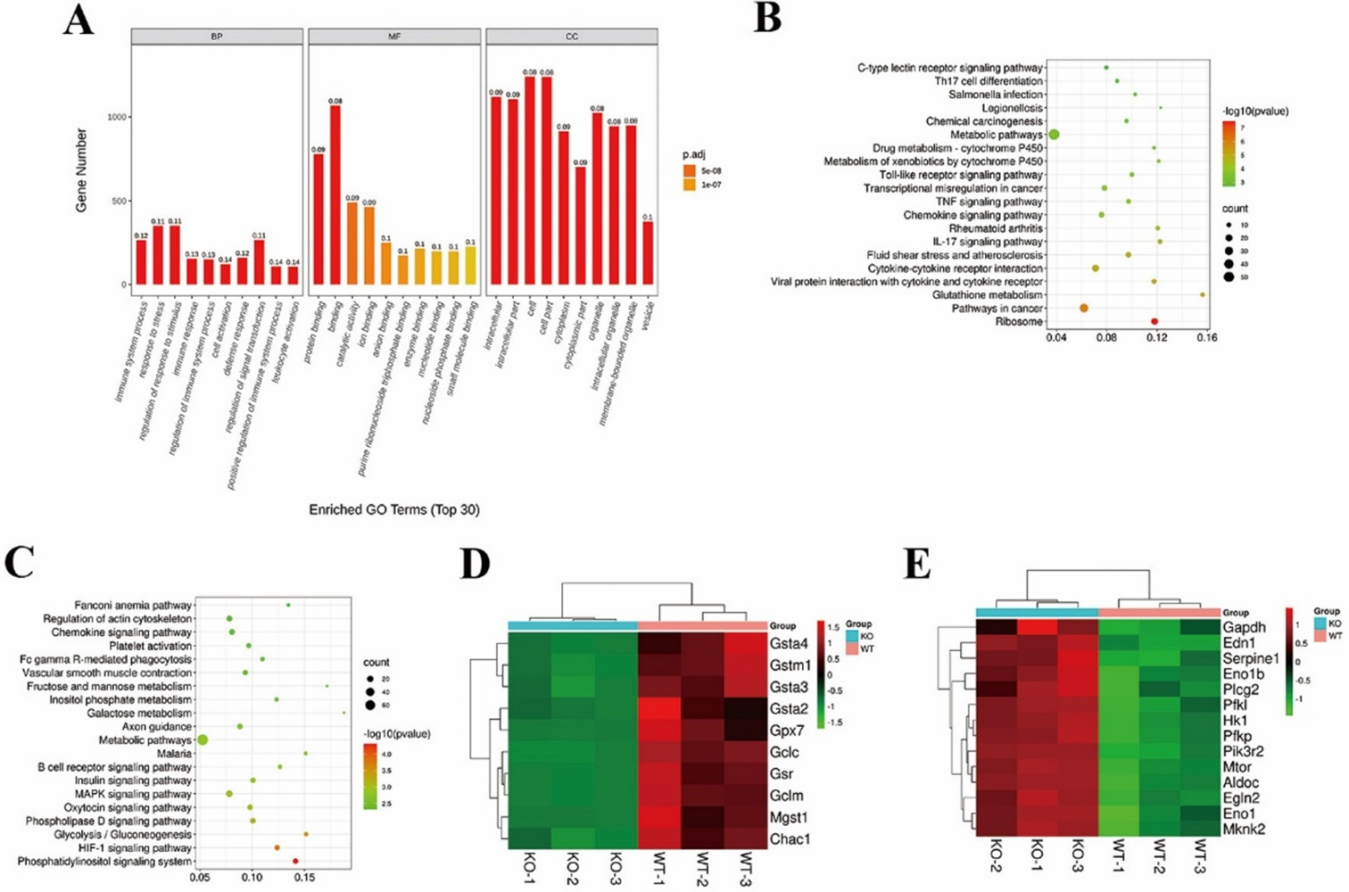
** TGR5 signaling regulates intracellular glutathione metabolism in DCs.** TGR5^+/+^ and TGR5^-/-^ BMDCs were treated with LCA. **A.** The top 10 significantly enriched GO terms: molecular function (MF), cellular component (CC) and biological process (BP) are presented. The x-axis shows the categories of GO terms and y-axis shows the Gene number. **B.** The top 10 up-regulated pathway enrichment analyses from the KEGG database. **C.** The top 10 down-regulated pathway enrichment analyses from the KEGG database. **D.** Heatmap of DEGs associated with Glutathione metabolism. **E.** Heatmap of DEGs associated with HIF-1 pathway.

We performed RNA-seq on LPS-stimulated TGR5^+/+^ BMDCs and TGR5^-/-^ BMDCs treated with LCA to determine how TGR5 signaling inhibits DC activation. Our analyses revealed 1584 differentially expressed genes (DEGs) in TGR5^+/+^ BMDCs compared with TGR5^-/-^ BMDCs, of which 641 DEGs were upregulated and 943 DEGs were downregulated (Table S1). GO enrichment analysis was performed according to three major categories: molecular function (MF), biological processes (BP) and cellular components (CC). A total of 423 GO terms were significantly enriched (FDR<0.01), and the top 30 GO terms are shown in Figure 6A. For the BP analysis, 329 enrichments were observed, the top-ranked of which included response to stress, immune system process and immune response. The MF category was mainly enriched in protein binding. The CC analysis included 54 GO terms, of which the identified genes were mostly enriched in intracellular, cytoplasm and organelle.

KEGG pathway enrichment analysis revealed that 24 significant pathways were upregulated in TGR5^+/+^ BMDC group, including ribosome expression and glutathione metabolism pathways and pathways in cancer (Figure 6B). In general, 12 pathways, including the phosphatidylinositol signaling system and the HIF-1 signaling pathway, were downregulated (Figure 6C). The DEGs of glutathione metabolism and the HIF-1 signaling pathway are shown in Figure 6D and Figure 6E.

### TGR5 signaling suppresses pro-inflammatory cytokine production by DCs by inhibiting glutathione production and inducing oxidative stress

**Figure 7:**
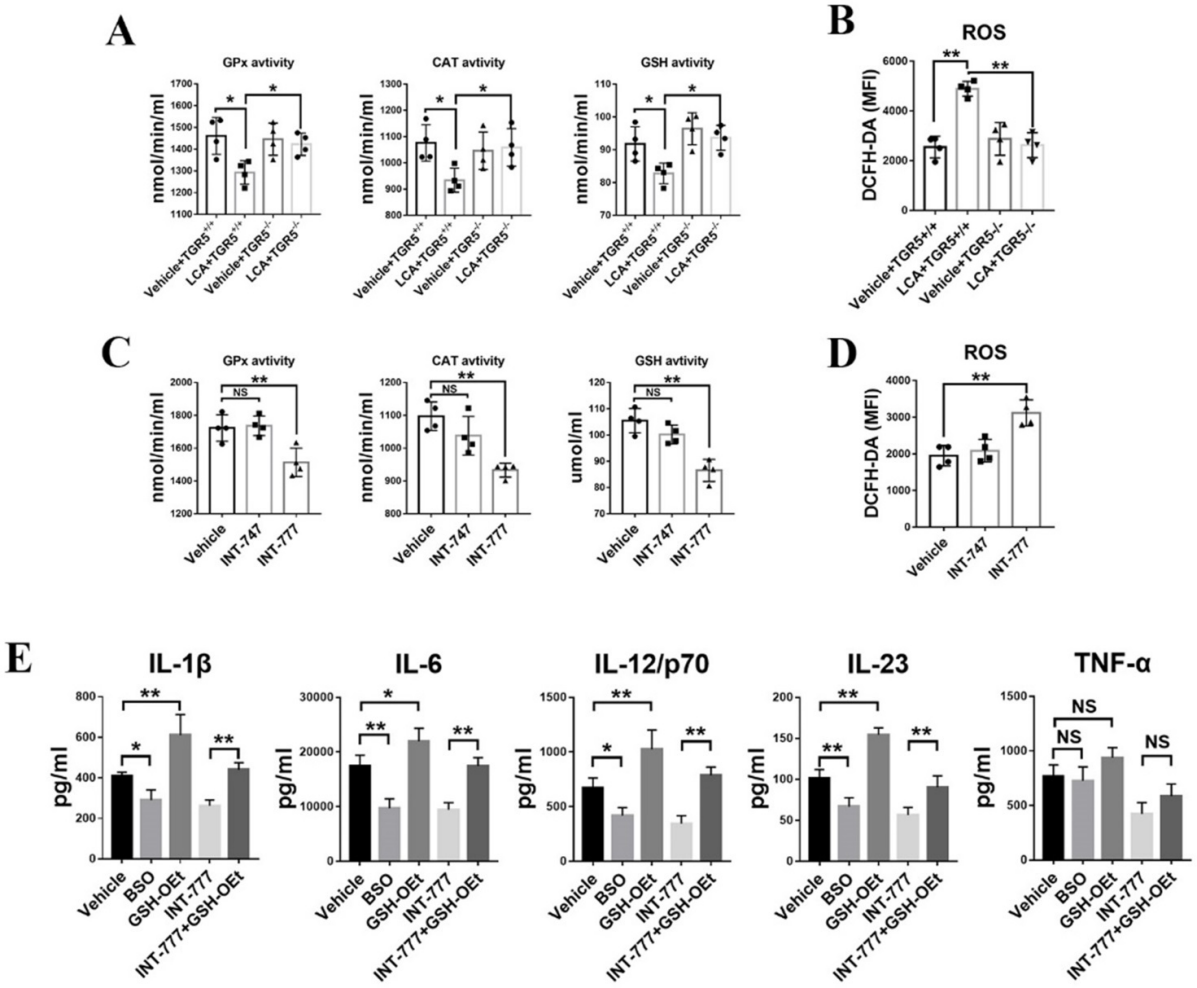
** TGR5 activation suppresses the secretion of pro-inflammatory cytokines in DCs by inhibiting glutathione production and inducing oxidative stress. A.** Catalytic enzyme activities of GPx, CAT and GSH in TGR5^+/+^ and TGR5^-/-^ BMDCs treated with LCA (n=4 per group). **B.** ROS levels in TGR5^+/+^ and TGR5^-/-^ BMDCs treated with LCA (n=4 per group). **C.** Catalytic enzyme activities of GPx, CAT and GSH in BMDCs treated with INT-777 and INT-747 (n=4 per group). **D.** ROS levels in in BMDCs treated with INT-777 and INT-747 (n=4 per group). **E.** The expression of IL-1β, IL-6, IL-12/p70, IL-23 and TNF-α in BMDCs primed with LPS and thereafter treated with BSO, GSH-OEt, INT-777 or GSH-OEt+INT-777 (n=4 per group). The analysis was performed using ELISA. Data are shown as mean ± SD. ns p>0.05, *p<0.05, **p<0.01.

Lack of glutathione inhibits DC maturation and the subsequent production of pro-inflammatory cytokines 22,24. Herein, we found that LCA significantly inhibited the activity of intracellular catalytic enzymes, including glutathione catalase (CAT), peroxidase (GPx) and glutathione (GSH) in TGR5^+/+^ BMDCs (Figure 7A). However, TGR5 deficiency disrupted the effect of LCA (Figure 7A). In contrast to INT-747, INT-777 also regulated the activity of intracellular catalytic enzymes in TGR5^+/+^ BMDCs (Figure 7C). These findings suggested that LCA disrupted the activity of antioxidant enzymes in DCs via TGR5 signaling. Intracellular ROS were stained with DCFH-DA and tested by FCM. The results showed that LCA induced ROS accumulation in TGR5^+/+^ BMDCs but had no effect on TGR5^-/-^ BMDCs (Figure 7B). Similar results were observed in INT-777-treated TGR5^+/+^ BMDCs (Figure 7D).

Then, L-buthionine-sulfoximine (BSO) and glutathione reduced form ethyl ester (GSH-OEt) were used to block or increase GSH expression in TGR5^+/+^ BMDCs, respectively. We found that GSH-OEt treatment induced the production of IL-6, IL-1β, IL-23 and IL-12p/70 (Figure 7E). However, BSO had the opposite effect (Figure 7E). Additionally, the expression levels of the above cytokines were higher in INT-777+ GSH-OEt treatment group than in INT-777 group (Figure 7E). Interestingly, neither GSH-OEt nor BSO affected TNF-α expression (Figure 7E).

ROS accumulation has been implicated in chronic inflammation 30. The levels of ROS and GSH in the retinal tissue of TGR5^-/-^ and TGR5^+/+^ EAU mice fed a diet with or without LCA were examined by fluorescence microscopy. However, LCA had no effect on ROS and GSH levels in the retinal tissues of EAU mice (Figures S5A-B).

### LCA promotes apoptosis and autophagy in DCs via TGR5 signaling

**Figure 8:**
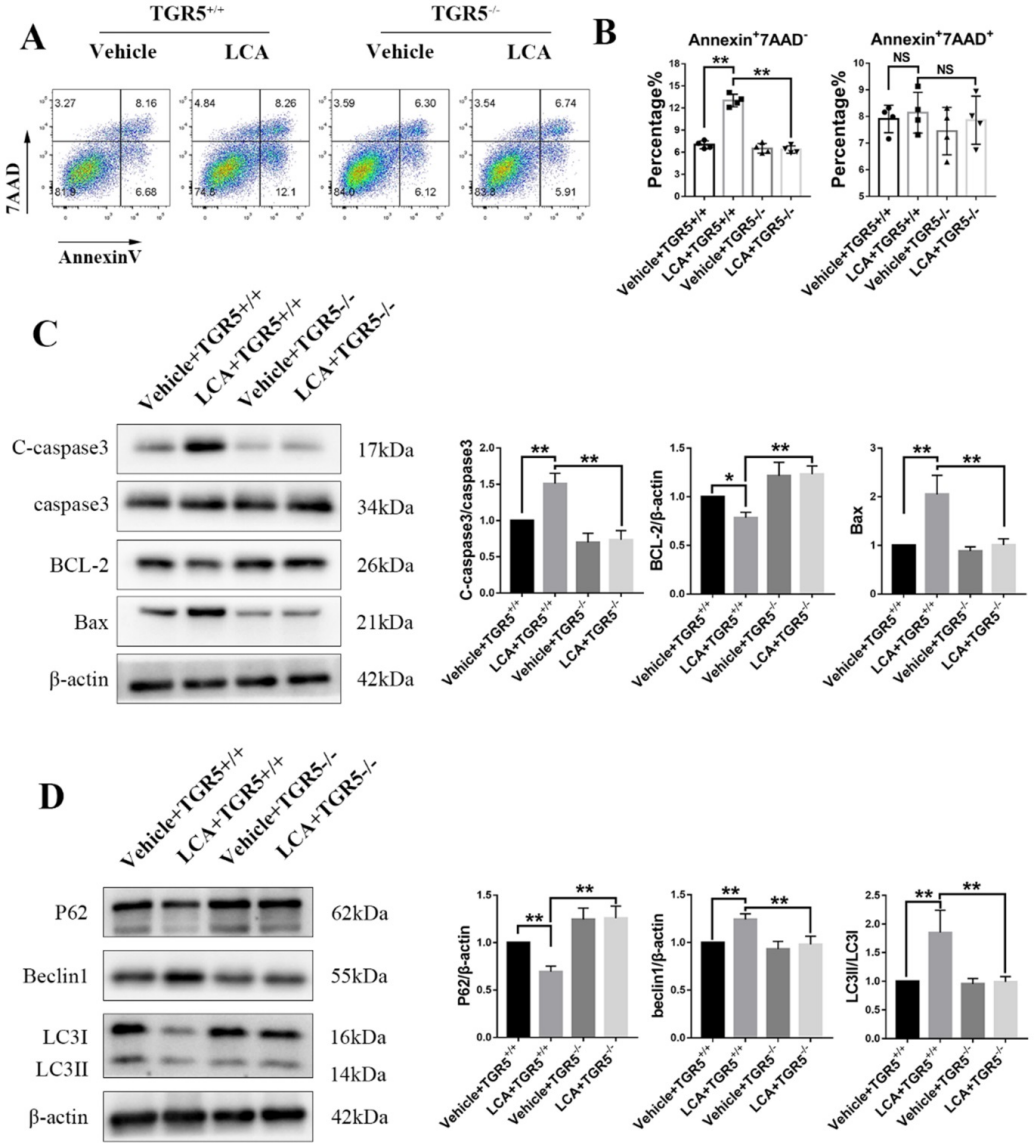
** LCA promotes apoptosis and autophagy in DCs via TGR5 signaling.** TGR5^+/+^ and TGR5^-/-^ BMDCs were treated with LCA. **A and B.** The proportion of apoptotic cells was analyzed using Flow cytometry (n=4 per group). **C.** The expression of Bax, Bcl-2 and cleaved caspase-3 protein in TGR5^+/+^ and TGR5^-/-^ BMDCs treated with LCA was measured by Western blot (n=3 per group). **D.** The expression of P62, Beclin1 and LC3II/LC3I proteins in TGR5^+/+^ and TGR5^-/-^ BMDCs treated with LCA was measured by Western blot (n=3 per group). Data are shown as mean ± SD. ns p>0.05, *p<0.05, **p<0.01.

In this study, we found that LCA slightly increased the proportion of Annexin+7AAD- cells in TGR5^+/+^ BMDCs (Figures 8A-B). TGR5 deficiency significantly reversed this effect (Figures 8A-B). LCA increased the expression of Bax and cleaved caspase-3 but decreased that of Bcl-2 in TGR5^+/+^ BMDCs, but not in TGR5^-/-^ BMDCs (Figure 8C). We further evaluated the expression of LC3 and Beclin-1 in BMDCs, two important proteins involved in autophagy. We found that LCA increased the LC3II/LC3I ratio and the expression of Beclin1, suggesting that LCA induces autophagy in BMDCs (Figure 8D). LCA also reduced the expression of P62, which was degraded during autophagy (Figure 8D). Similarly, inhibition of TGR5 reversed the effect of LCA on autophagy (Figure 8D). MAPK and NF-κB pathways are involved in the pathogenesis of cell apoptosis and autophagy. We found that LCA regulated the phosphorylation of P38 and ERK1/2 and inhibited NF-κB activation in TGR5^+/+^ BMDCs. However, LCA had no effect on these pathways in TGR5^-/-^ BMDCs (Figure S7A-B).

To explore whether GSH is involved in TGR5-mediated apoptosis and autophagy, TGR5^+/+^ BMDCs were treated with vehicle, BSO, INT-777 or INT-777+GSH-OEt. We found that both INT-777 and BSO reduced the proportion of Annexin+7AAD- cells (Figures S6A-B). GSH-OEt significantly reversed the inhibitory effect of INT-777 (Figures S6A-B). Compared with vehicle or INT-777+ GSH-OET group, INT-777 or BSO significantly decreased the level of BCL-2 and increased the expression of Bax and cleaved caspase-3 (Figure S6C), and increased the LC3II/LC3I ratio and the expression of Beclin1 and decreased the level of P62 (Figure S6D). In addition, the phosphorylation levels of ERK1/2, P38, NF-κB and IκBα were higher in INT-777 and BSO groups (Figures S8A-B).

## Discussion

BAs are known as integrators of metabolism, and emerging evidence has shown that BAs are important signaling molecules in the immune response 7,8,19. BAs are thought to maintain immune homeostasis via several nuclear receptors and/or G-protein-coupled receptors (GPCRs) 7. Herein, we found that BAs inhibit the activation of DCs via the TGR5 receptor and regulate autoimmune uveitis. In addition, we found that BAs and TGR5 signaling promote intracellular GSH depletion, which disrupts the intracellular redox status in DCs.

The anti-inflammatory roles of secondary BAs were first described in chronic cholestasis, characterized by under-secretion of TNF-α, IL-6 and IL-1β and by macrophages 31,32. Secondary BAs modulate the secretion of IL-12 by DCs, inhibiting the differentiation of Th17 cells and promoting the differentiation of Treg cells 9,10. In the present study, we found that several BAs, including CA, CDCA, LCA and DCA, inhibited the production of pro-inflammatory cytokines in DCs. In addition to the *in vitro* studies, dysmetabolism of bile acid was also observed in patients with IBD, while it is worth mentioning that only secondary BAs (DCA and LCA) inhibited the secretion of IL-18 and IL-1β in intestinal epithelial cells 11. Plasma BAs are significantly low in MS, and supplementation with TUDCA, can ameliorate the severity of EAE 13. In this study, the mouse model revealed that dietary intake of LCA significantly alleviated the severity of EAU by decreasing the expression of Th17, Th1 cells and CD11c^+^MHCII^high^ DCs.

BAs exert anti-inflammatory effects by activating the expression of BA receptors such as GPCRs as well as nuclear receptors 7. TGR5 and FXR are the main receptors for BAs and belong to the GPCR families and nuclear receptor, respectively 7. LCA and DCA are the preferred ligands for TGR5, while CA and CDCA are the most potent FXR ligands 7. INT-747 has been shown to inhibit the activation and differentiation of intestinal DCs, and exacerbated the severity of colitis in FXR^-/-^ mice 33,34. Current study showed that TGR5 agonists promoted the differentiation of monocytes to IL-12 hypo-producing DCs 9. Herein, we found that LCA mostly increased the expression of FXR and TGR5 in BMDCs, whereas TGR5 agonists suppressed the production of IL-23, IL-1β, IL-12, IL-6, and TNF-α. Meanwhile, the FXR agonist only affected the expression of IL-12, IL-1β and TNF-α. However, TGR5-siRNAs reversed the effect of LCA on the secretion of pro-inflammatory cytokines in BMDCs, and the effect was greater than that of FXR-siRNAs. These findings suggest that LCA is the most significant inhibitor of pro-inflammatory cytokine secretion via TGR5 signaling. However, LCA had no effect on the activation and proliferation of CD4+ T cells *in vitro*, which was caused by the low expression of TGR5 in T cells.

Glutathione, a major component of the antioxidant defense system of living cells, is present in both prokaryotes and eukaryotes. GSH and oxidative stress are thought to play a key role in immunity, including autoimmunity 35-37. Studies have shown that GSH depletion can promote apoptosis of CD4+ T lymphocytes and influence CD4+ T-cell proliferation via the mTOR pathway by increasing the expression of ROS 30,38,39. GSH depletion promotes the release of pro-inflammatory cytokines in LPS-induced epithelial cells 36,40. In contrast, GSH depletion regulates the adaptive immune response by inhibiting the secretion of IL-12 and IL-27 and disrupting the expression of surface molecules in BMDCs 23,41. GSH depletion and oxidative stress reduce the expression of IL-1β, TNF-α and IL-6 in LPS-induced macrophages 42. Collectively, these findings demonstrate that glutathione performs various functions in different cell types. Consistent with the above findings, we found that TGR5 activation in BMDCs modulates the expression of GSH, whereas inhibition of GSH by BSO significantly reduces the production of pro-inflammatory cytokines in BMDCs. This in turn regulates the antigen-presenting ability of DCs. However, inhibition of GSH expression results in ROS accumulation, which participates in the maturation of DCs and upregulates the expression of costimulatory molecules 43-46. Sangyong Jon et al. found that ROS scavenging using bilirubin suppresses the expression of MHC II and costimulatory molecules on DCs 47. Therefore, we hypothesize that overexpression of ROS resulting from the inhibition of GSH expression would promote apoptosis and autophagy in DCs. Emerging evidence also suggests that apoptosis and autophagy play important roles in inhibiting DC maturation 48-50. Furthermore, increased levels of ROS are often found in chronic inflammatory entities 30. However, dietary intake of LCA or inhibition of TGR5 has no effect on GSH expression and ROS accumulation in the retinas of EAU mice, suggesting that LCA may not be able to cross the blood-retinal barrier.

In macrophages, TGR5 inhibits the activation of NF-κB via the cAMP-PKA pathway 19. Notably, this inhibition is dependent on the secretion of IL-10 induced by CREB 19,28,51. In addition, we found that TGR5 signaling inhibited the phosphorylation of IκBα, which released the NFκB-p65 subunit in DCs. However, LCA and TGR5 agonists had no effect on the production of IL-10 and the phosphorylation of CREB in DCs. Glutathionylation is known to play an important role in regulating the activity of NF-κB and IκBα 52, and glutathionylation of IKK modulates the LPS-induced release of cytokines 53. Inhibition of GSH induced by HEMA has no effect on LPS-induced phosphorylation of NF-κB/p65 and IκBα in macrophages 54. In this study, we further observed that TGR5 agonist or BSO inhibited NF-κB activation, whereas GSH reversed the inhibitory effect of NF-kb activation induced by TGR5 agonist. In addition to NF-κB, p38/MAPK and ERK/MAPK are important target molecules for TGR5 signaling, which was also downregulated by GSH depletion.

TGR5 was found decreased in macrophages of active VKH patients and decreased in DCs of active BD patients 55. Both INT-777 and LCA suppressed the inflammatory functions of MD-DCs from BD patients. These findings further support the consensus that secondary BAs and TGR5 signaling play crucial roles in autoimmune and inflammatory diseases. Thus, TGR5 is a potential therapeutic target for the control and treatment of autoimmune and inflammatory diseases.

## Conclusions

In summary, BAs inhibit the expression of pro-inflammatory cytokines and surface markers in DCs, with LCA exerting the greatest effect. Although FXR can be activated by LCA, TGR5 is the most potent downstream target of LCA. LCA inhibits DC activation through TGR5 signaling to regulate the development of autoimmune uveitis. This effect of LCA is associated with a decreased intracellular GSH in DCs. Inhibition of GSH attenuates the release of pro-inflammatory cytokines via the NF-κB and MAPK pathways. Additionally, LCA promoted apoptosis and autophagy in DCs by inhibiting GSH. Therefore, LCA and TGR5 agonists are potential therapeutic drugs for the treatment of autoimmune and inflammatory diseases, including autoimmune uveitis.

## Supplementary Material

Supplementary figures and tables.

## Abbreviations

EAU: Experimental autoimmune uveoretinitis
BMDCs: bone marrow derived dendritic cells
DCA: deoxycholic acid
BD: Behcet's disease
VKH: Vogt-Koyanagi-Harada
AAU: acute anterior uveitis
IBD: inflammatory bowel disease
T1D: type 1 diabetes
CFA: complete Freund's adjuvant
CA: Cholic acid
LCA: lithocholic acid
CDCA: Chenodeoxycholic acid
VDR: vitamin D receptor
TGR5: Takeda G-protein coupled receptor 5
GR: glucocorticoid receptor
CAR: constitutive androstane receptor
FXR: farsenoid X receptor
PXR: pregnane X receptor
CREB: cAMP-response element binding protein
MD-DCs: human monocytes derived dendritic cells
