# Supplementary material for: Lithocholic acid inhibits dendritic cell activation by reducing intracellular glutathione via TGR5 signaling
Source: Int J Biol Sci. 2022 Jul 11;18(11):4545–59. doi: 10.7150/ijbs.71287 (PMC9295063; doi:10.7150/ijbs.71287)
Supplement: Supplementary file 1 — Supplementary figures and tables. [file ijbsv18p4545s1.pdf]

Supplementary Materials

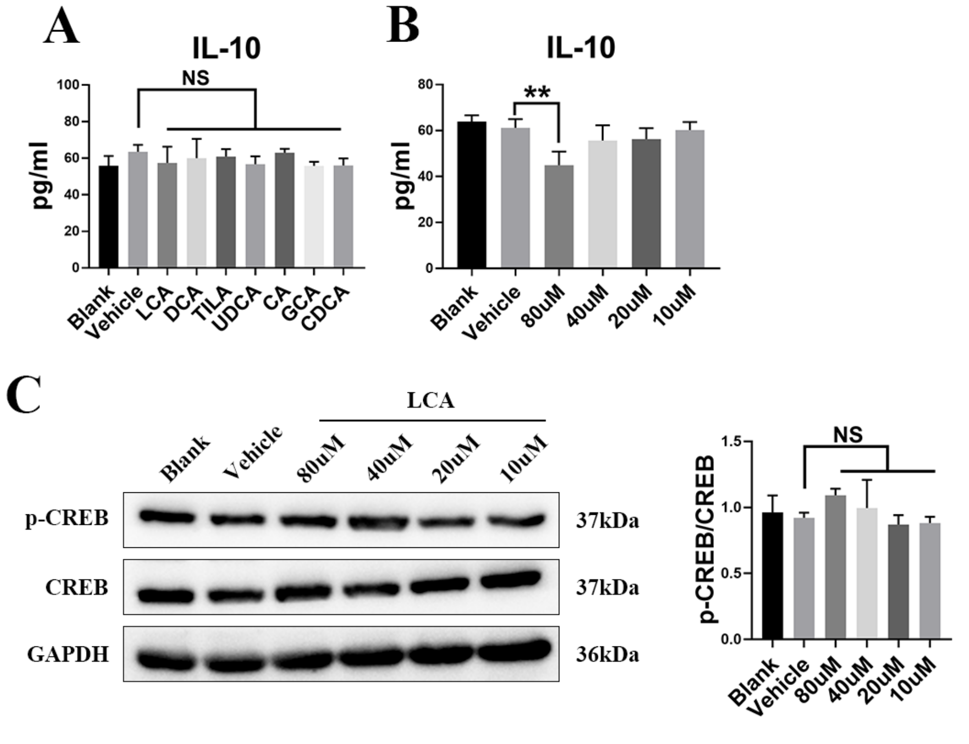

Figure S1. The effect of LCA on the secretion of anti-inflammatory cytokine IL-10 in BMDCs. A. The expression of IL-10 in BMDCs primed with BMDCs and thereafter treated with selected BAs at doses of 10μM was assayed by ELISA (n=4). **B.** The secretion of IL-10 in BMDCs primed with LPS and thereafter treated with LCA at doses of 80μM, 40μM, 20μM and 10μM. The analysis was performed using ELISA (n=4). **C.** Western blot analysis for the expression of P-CREB and CREB proteins in BMDCs primed with LPS and thereafter treated with varied LCA dosages. Data are shown as mean ± SD. ns p>0.05, *p<0.05, **p<0.01.

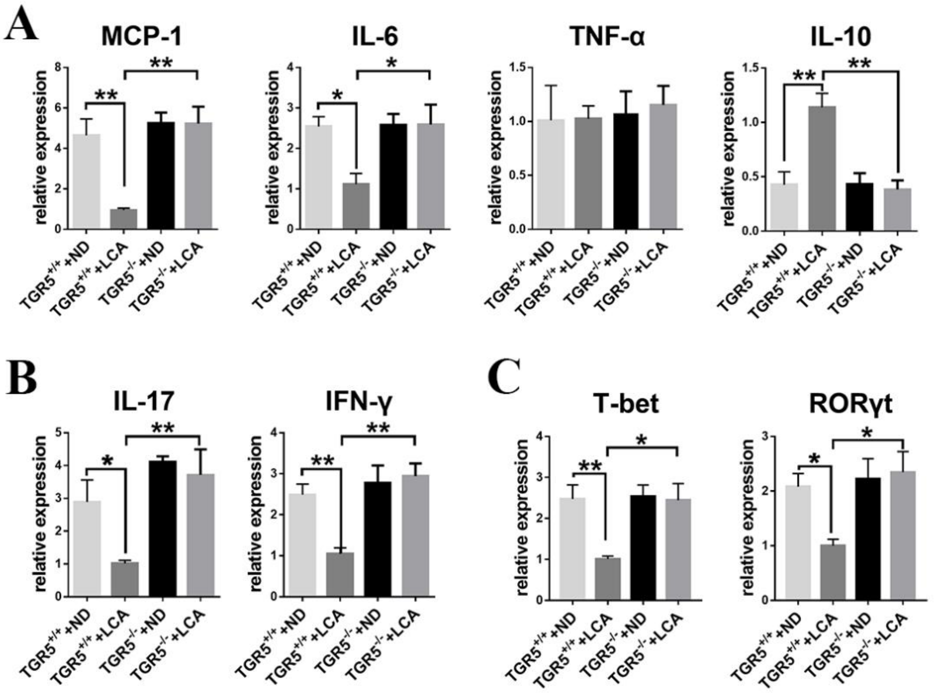

Figure S2. LCA inhibited pro-inflammatory cytokines expression and induced anti-inflammatory cytokine expression in the retina of EAU via TGR5 signaling. **A.** The relative mRNA expression of MCP-1, IL-6, IL-10 and TNF-αin the retinal tissues of EAU mice was tested by RT-qPCR ($n \geq 4$ per group). **B.** The relative mRNA expression of IFN-γ and IL-17 (Th1 and Th17) in the retinal tissues of EAU mice was tested by RT-qPCR ($n \geq 4$ per group). **C.** The relative mRNA expression of transcription factors T-bet and RORγT in the retinal tissues of EAU mice was tested by RT-qPCR ($n \geq 4$ per group). Data are shown as mean ± SD. ns $p>0.05$, *$p<0.05$, **$p<0.01$.

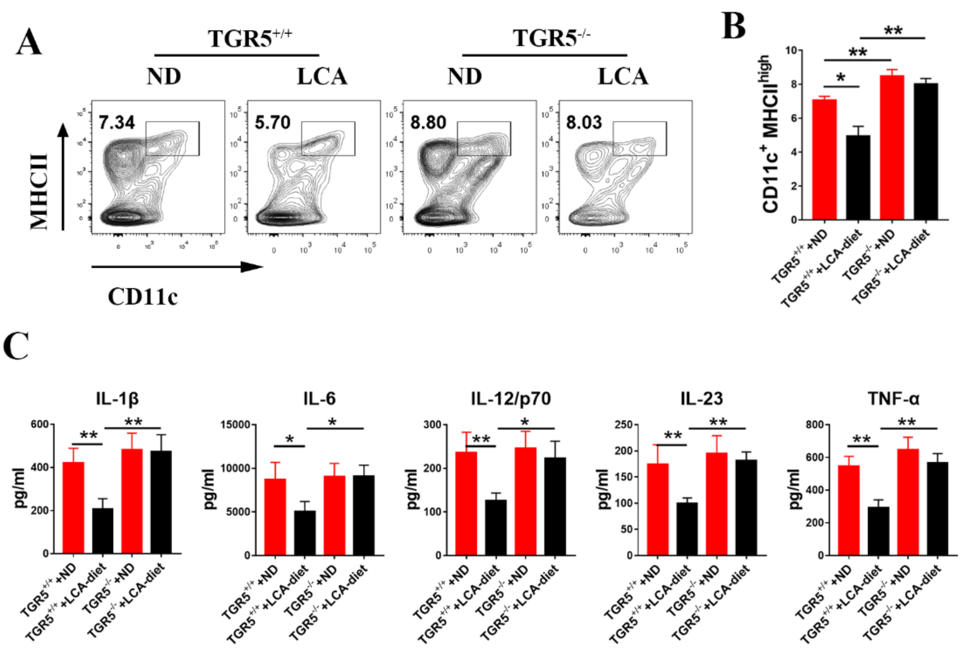

Figure S3. LCA inhibited the activation of splenic DCs in EAU mice via TGR5 signaling. TGR5$^{+/+}$ and TGR5$^{-/-}$ mice were injected with IRBP$_{651-670}$ and CFA, and thereafter fed with LCA-diet (n=4-6 per group). **A and B.** Flow cytometric analysis for the proportion of CD11c^{+}MHC^{high} DCs cells in splenocytes of EAU mice. **C.** The expression of IL-1β, IL-6, IL-12/p70, IL-23 and TNF-α in LPS primed CD11c^{+} cells isolated from splenocytes of EAU mice was tested by ELISA. Data are shown as mean ± SD. ns p>0.05, *p<0.05, **p<0.01.

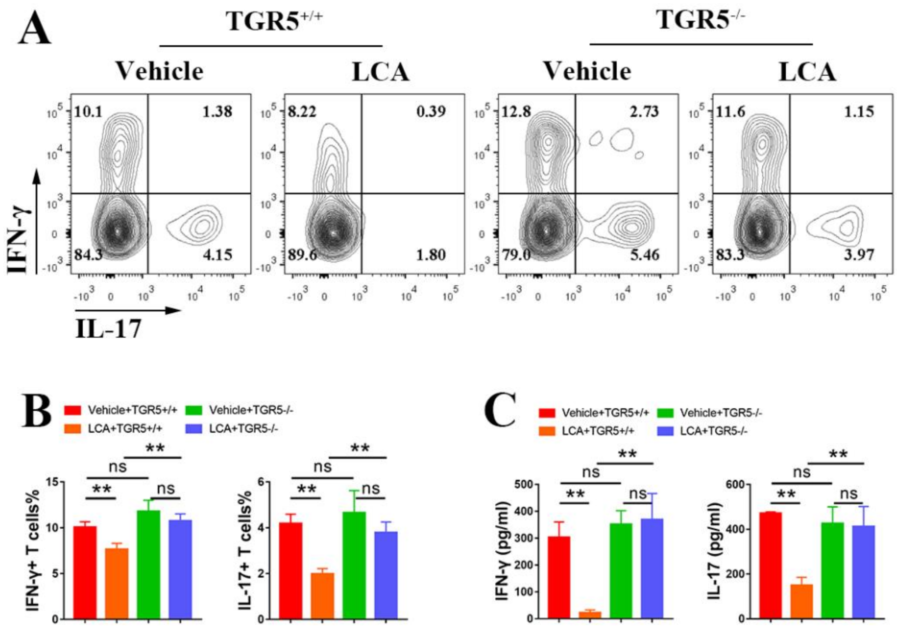

Figure S4. LCA regulates the antigen presentation function of DC function via TGR5 signaling pathway. TGR5$^{+/+}$ and TGR5$^{-/-}$ BMDCs were pre-treated with LCA and thereafter co-cultured with naïve T cells (BMDCs: Naïve T; 1: 5) (n=4 per group). **A and B**. Flow cytometric analysis for the proportion of Th1 and Th17 cells. **C.** The level of IL-17 and IFN-γ in cultured supernatants was tested by ELISA**.** Data are shown as mean ± SD. ns p>0.05, *p<0.05, **p<0.01.

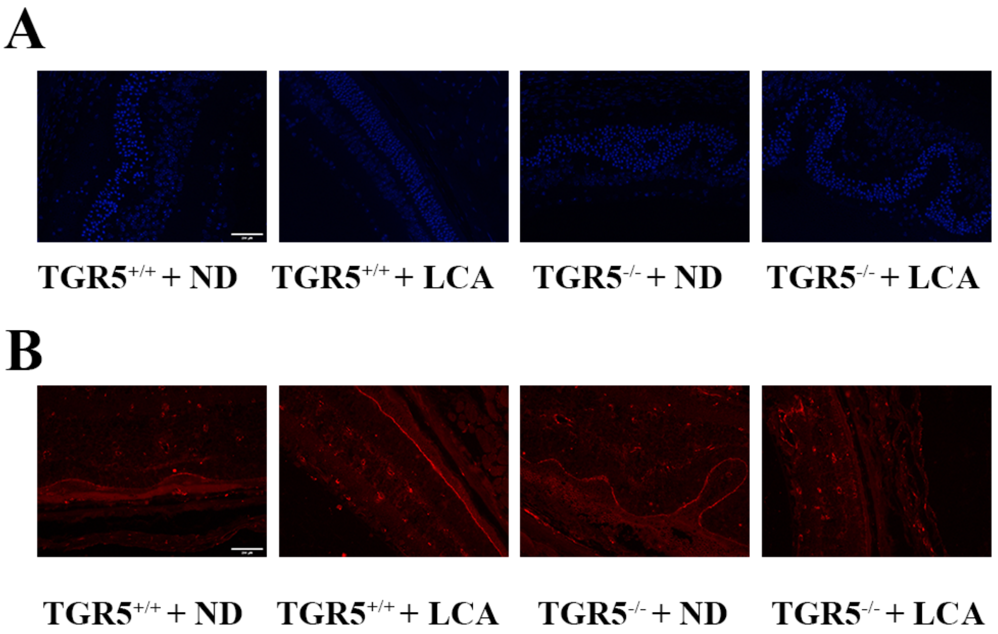

Figure S5. LCA-diet had no effect on ROS and GSH in the retina of EAU mice TGR5$^{+/+}$ mice and TGR5$^{-/-}$ mice were injected with IRBP$_{651-670}$ and CFA, and thereafter fed on LCA-diet. **A.** GSH level in the retinal tissues of EAU mice. **B.** ROS level in the retinal tissues of EAU mice. Scale bar 200μM.

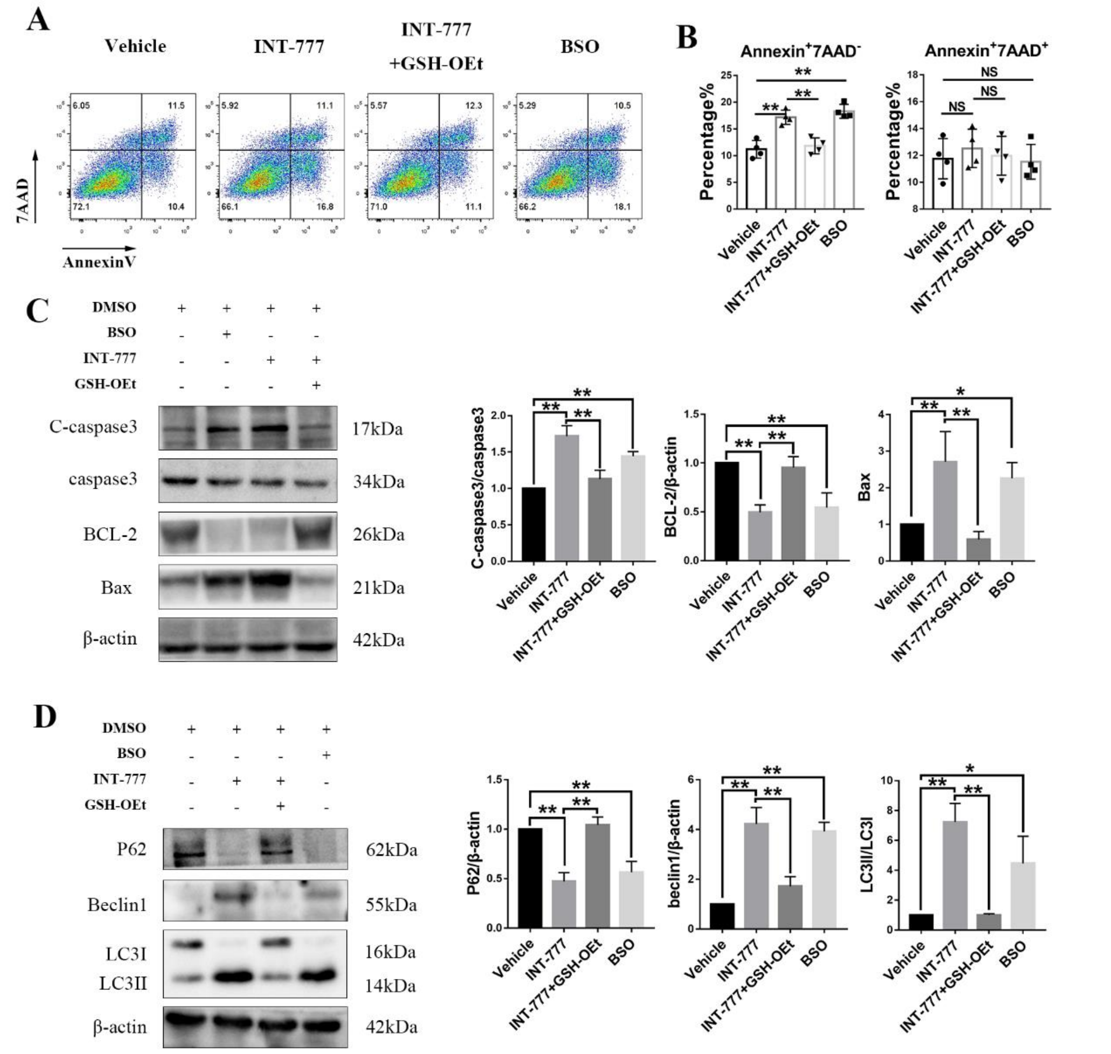

Figure S6. TGR5 promotes apoptosis and autophagy of DCs by inhibiting production of GSH. BMDCs were treated with Vehicle, INT-777, BSO or INT-777+BSO. **A and B.** Flow cytometric analysis for the proportion of apoptotic cells using AnnexinV-APC/7AAD. **C.** The level of Bcl-2 and cleaved caspase-3 protein was measured by Western blot. **D.** The level of P62, Beclin1 and LC3II/I protein level was measured by Western blot. Data are shown as mean ± SD. ns $p>0.05$, *$p<0.05$, **$p<0.01$.

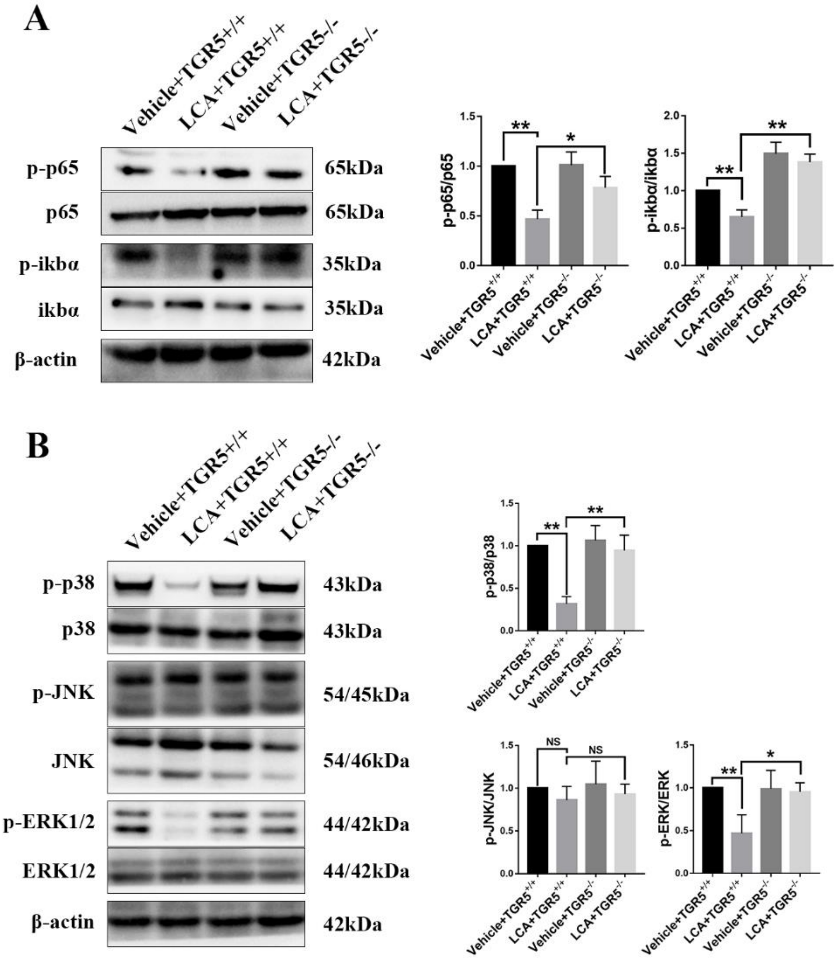

Figure S7. TGR5 signaling inhibits activation of NF-κb pathway and MAPK pathways in DCs. TGR5$^{+/+}$ and TGR5$^{-/-}$ BMDCs were treated with LCA**. A.** The phosphorylation level of P65 and Iκbα in DCs was assayed by western blot. **B.** The phosphorylation level of P38, ERK1/2 and JNK in DCs was assayed by western blot. Data are shown as mean ± SD. ns $p>0.05$, *$p<0.05$, **$p<0.01$.

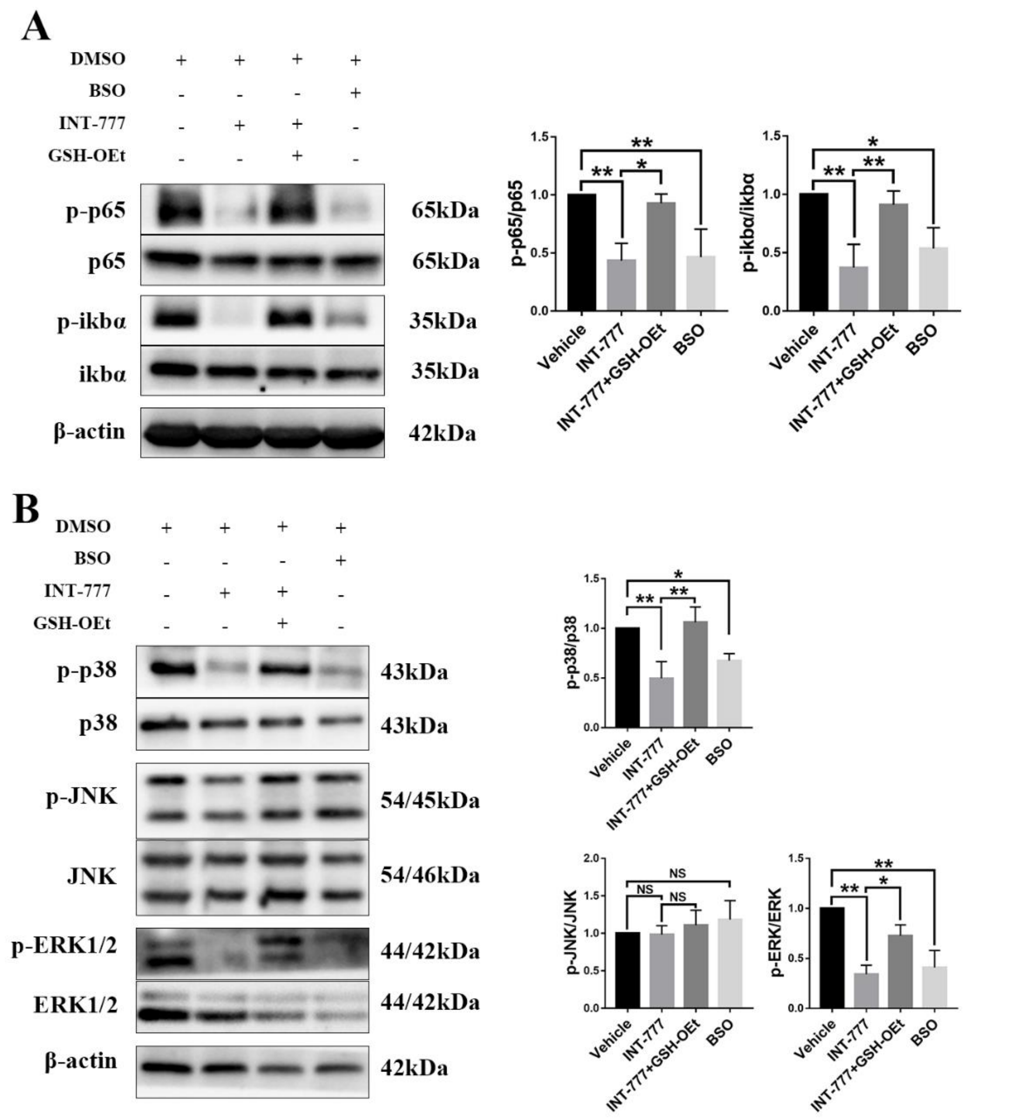

Figure S8. GSH depletion inhibited activation of NF-κb pathway and MAPK pathways in DCs. BMDCs were treated with Vehicle, INT-777, BSO or INT-777+BSO. **A.** The phosphorylation levels of P65 and Iκbα was assayed by western blot. **B.** The phosphorylation of P38, ERK1/2 and JNK was assayed by western blot. Data are shown as mean ± SD. ns $p>0.05$, *$p<0.05$, **$p<0.01$.

Table S1. Full list of Differentially expressed genes between TGR5$^{+/+}$ and TGR5$^{-/-}$ comparison

gene_id	Log^2 Fold Change	P-value adj	Symbol
ENSMUSG00000087064	-5.396	0.002455	Sap30bpos
ENSMUSG00000090077	-4.9253	0.012995	Lime1
ENSMUSG00000030340	-4.5139	0.016334	Scnn1a
ENSMUSG00000033170	-4.2928	0.026456	Card10
ENSMUSG00000085887	-3.8432	6.76E-05	Arhgap27os3
ENSMUSG00000051378	-3.8254	0.017514	Kif18b
ENSMUSG00000108444	-3.6341	1.44E-06	Klk2-ps
ENSMUSG00000083907	-3.5228	0.007723	Plk-ps1
ENSMUSG00000040703	-3.489	0.004171	Cyp2s1
ENSMUSG00000070999	-3.3325	0.001641	Ccin
ENSMUSG00000041734	-3.33	2.41E-05	Kirrel
ENSMUSG00000092528	-3.0159	0.002986	Nlrp1c-ps
ENSMUSG00000086965	-3.012	0.023891	Rtl10
ENSMUSG00000026829	-2.9758	0.000177	Gbgt1
ENSMUSG00000029074	-2.9598	0.000211	Ttll10
ENSMUSG00000026180	-2.9054	2.10E-22	Cxcr2
ENSMUSG00000031970	-2.8997	2.76E-05	Dbndd1
ENSMUSG00000031907	-2.8647	5.88E-07	Zfp90
ENSMUSG00000097405	-2.8102	0.016712	D630044L22Rik
ENSMUSG00000034227	-2.7983	0.003715	Foxj1
ENSMUSG00000001506	-2.7162	0.00034	Col1a1
ENSMUSG00000052435	-2.674	0.011487	Cebpe
ENSMUSG00000032484	-2.6637	0.000192	Ngp
ENSMUSG00000105096	-2.6439	4.90E-06	Gbp10
ENSMUSG00000003348	-2.6009	6.88E-09	Mob3a
ENSMUSG00000079019	-2.5864	0.010431	Insl3
ENSMUSG00000074577	-2.5783	0.030391	Ripor3
ENSMUSG00000041945	-2.5768	0.000122	Mfsd9
ENSMUSG00000061577	-2.5751	0.00562	Adgrg5
ENSMUSG00000090326	-2.5133	3.87E-06	Dthd1
ENSMUSG00000006445	-2.5048	5.23E-05	Epha2
ENSMUSG00000035773	-2.4635	0.011015	Kiss1r
ENSMUSG00000027932	-2.439	2.67E-26	Slc27a3
ENSMUSG00000114235	-2.4372	5.93E-07	F530104D19Rik
ENSMUSG00000100658	-2.4353	1.36E-12	F730311O21Rik
ENSMUSG00000002799	-2.4237	2.64E-17	Jag2
ENSMUSG00000096954	-2.4133	7.84E-31	Gdap10
ENSMUSG00000030890	-2.3913	0.015701	Ilk
ENSMUSG00000049577	-2.3897	2.84E-06	Zfpm1
ENSMUSG00000045322	-2.3847	2.44E-12	Tlr9
ENSMUSG00000021823	-2.3401	4.29E-27	Vcl
ENSMUSG00000067642	-2.3279	0.035155	Adgrf3
ENSMUSG00000078923	-2.3255	0.000155	Ube2v1
ENSMUSG00000049103	-2.3203	5.98E-26	Ccr2
ENSMUSG00000081801	-2.2831	0.028528	Dnmt3l-ps1
ENSMUSG00000024013	-2.2551	3.91E-12	Fgd2
ENSMUSG00000021279	-2.2471	1.87E-28	Cdc42bpb
ENSMUSG00000018341	-2.2308	3.00E-28	Il12rb2

ENSMUSG00000021256	-2.2218	1.32E-14	Vash1
ENSMUSG00000000248	-2.2187	2.52E-06	Clec2g
ENSMUSG00000046541	-2.2003	2.71E-08	Zfp526
ENSMUSG00000044867	-2.1962	0.007266	Gimap1os
ENSMUSG00000074259	-2.1938	4.29E-06	Gramd2
ENSMUSG00000114598	-2.1865	1.46E-05	D130062J10Rik
ENSMUSG00000039834	-2.1848	2.24E-12	Zfp335
ENSMUSG00000031822	-2.1785	1.50E-23	Gse1
ENSMUSG00000024186	-2.1696	8.19E-09	Rgs11
ENSMUSG00000022066	-2.1676	0.020635	Entpd4b
ENSMUSG00000091311	-2.1556	3.28E-07	Spata31d1b
ENSMUSG00000043557	-2.1535	0.034771	Mdga1
ENSMUSG00000028874	-2.1438	2.46E-06	Fgr
ENSMUSG00000046844	-2.1263	0.000556	Vat1l
ENSMUSG00000090231	-2.1247	0.001159	Cfb
ENSMUSG00000034311	-2.1198	1.04E-05	Kif4
ENSMUSG00000024936	-2.1188	0.000273	Kcnk7
ENSMUSG00000067786	-2.1168	3.75E-08	Nnat
ENSMUSG00000028458	-2.1151	6.99E-16	Tesk1
ENSMUSG00000085664	-2.089	7.92E-05	Atxn7l1os2
ENSMUSG00000041538	-2.0829	2.79E-24	H2-Ob
ENSMUSG00000059022	-2.0733	4.42E-07	Kcp
ENSMUSG00000014444	-2.0639	6.88E-08	Piezo1
ENSMUSG00000035835	-2.0583	6.70E-06	Plppr3
ENSMUSG00000003344	-2.0567	1.26E-18	Btbd2
ENSMUSG00000034872	-2.0539	0.000766	Gipc3
ENSMUSG00000015127	-2.0433	3.91E-12	Unkl
ENSMUSG00000031805	-2.0432	1.28E-18	Jak3
ENSMUSG00000029607	-2.0381	0.000145	Ankrd61
ENSMUSG00000025500	-2.038	4.33E-06	Lmntd2
ENSMUSG00000026944	-2.0267	4.01E-16	Abca2
ENSMUSG00000028078	-2.0163	1.35E-06	Dclk2
ENSMUSG00000067341	-2.0148	0.003045	H2-Eb2
ENSMUSG00000037509	-2.0008	0.000502	Arhgef4
ENSMUSG00000001227	-2.0007	3.04E-07	Sema6b
ENSMUSG00000039508	-1.9989	0.016432	Calhm4
ENSMUSG00000032363	-1.9977	4.42E-09	Adamts7
ENSMUSG00000017718	-1.9871	1.15E-09	Afmid
ENSMUSG00000034917	-1.9847	4.06E-10	Tjp3
ENSMUSG00000030830	-1.9838	3.90E-51	Itgal
ENSMUSG00000082016	-1.9799	0.000105	Pgam1-ps2
ENSMUSG00000038665	-1.9706	0.028504	Dgki
ENSMUSG00000041954	-1.9651	6.83E-26	Tnfrsf18
ENSMUSG00000038880	-1.9646	5.06E-06	Mrps34
ENSMUSG00000090062	-1.9581	0.012227	Galnt6os
ENSMUSG00000029228	-1.9555	0.002986	Lnx1
ENSMUSG00000022801	-1.9532	1.73E-21	Lrch3
ENSMUSG00000024785	-1.9371	7.51E-18	Rcl1
ENSMUSG00000024913	-1.9346	1.64E-05	Lrp5
ENSMUSG00000056724	-1.9319	5.82E-28	Nbeal2
ENSMUSG00000018381	-1.9289	8.28E-05	Abi3
ENSMUSG00000001098	-1.9287	1.33E-18	Kctd10
ENSMUSG00000020684	-1.9247	0.036622	Rasl10b

ENSMUSG00000066363	-1.9237	7.23E-08	Serpina3f
ENSMUSG00000037341	-1.9125	2.13E-10	Slc9a7
ENSMUSG00000021880	-1.9057	8.82E-28	Rnase6
ENSMUSG00000046908	-1.9053	7.78E-11	Ltb4r1
ENSMUSG00000020135	-1.9021	0.019812	Apc2
ENSMUSG00000079487	-1.8981	1.18E-25	Med12
ENSMUSG00000068874	-1.8978	3.40E-11	Selenbp1
ENSMUSG00000027931	-1.8899	4.18E-24	Npr1
ENSMUSG00000035852	-1.8891	0.026029	Misp
ENSMUSG00000037012	-1.8771	6.98E-22	Hk1
ENSMUSG00000021950	-1.8756	1.37E-07	Anxa8
ENSMUSG00000062028	-1.8697	6.94E-05	Irgc1
ENSMUSG00000036882	-1.8632	1.01E-05	Arhgap33
ENSMUSG00000032741	-1.8585	3.50E-18	Tpcn1
ENSMUSG00000084984	-1.8584	1.47E-07	Far1os
ENSMUSG00000018169	-1.8562	9.63E-05	Mfng
ENSMUSG00000020527	-1.8531	5.74E-07	Myo19
ENSMUSG00000029718	-1.8489	0.006353	Pcolce
ENSMUSG00000021702	-1.8449	0.019899	Thbs4
ENSMUSG00000064177	-1.8435	0.031834	Ghrl
ENSMUSG00000066357	-1.84	5.78E-14	Wdr6
ENSMUSG00000000673	-1.8371	6.53E-13	Haao
ENSMUSG00000035863	-1.8371	1.17E-07	Palm
ENSMUSG00000024622	-1.8368	5.72E-12	Hmgxb3
ENSMUSG00000006398	-1.834	0.035955	Cdc20
ENSMUSG00000042388	-1.8299	2.69E-07	Dlgap3
ENSMUSG00000025153	-1.8266	3.17E-19	Fasn
ENSMUSG00000067399	-1.8231	0.001671	Trim43c
ENSMUSG00000033703	-1.8226	7.94E-10	Fuk
ENSMUSG00000030200	-1.8189	2.62E-07	Bcl2l14
ENSMUSG00000024130	-1.8169	3.20E-18	Abca3
ENSMUSG00000000157	-1.8122	1.31E-08	Itgb2l
ENSMUSG00000029088	-1.8078	0.02957	Kcnip4
ENSMUSG00000034177	-1.8074	5.41E-09	Rnf43
ENSMUSG00000000552	-1.8041	5.96E-12	Zfp385a
ENSMUSG00000059810	-1.7957	5.70E-13	Rgs3
ENSMUSG00000004815	-1.7955	4.11E-11	Dgkq
ENSMUSG00000017386	-1.7906	1.17E-11	Traf4
ENSMUSG00000024978	-1.7904	7.16E-08	Gpam
ENSMUSG00000058709	-1.7888	1.67E-17	Egln2
ENSMUSG00000033227	-1.7875	0.003298	Wnt6
ENSMUSG00000031722	-1.7857	0.00011	Hp
ENSMUSG00000022843	-1.7843	0.002443	Clcn2
ENSMUSG00000003190	-1.777	1.48E-09	Bcl2l12
ENSMUSG00000074811	-1.7761	1.11E-12	Hps6
ENSMUSG00000028776	-1.7716	0.000235	Tinagl1
ENSMUSG00000074628	-1.7712	0.034166	Tldc2
ENSMUSG00000038406	-1.7698	8.29E-06	Scaf1
ENSMUSG00000029163	-1.7658	4.50E-16	Emilin1
ENSMUSG00000037552	-1.7634	1.30E-11	Plekhg2
ENSMUSG00000049562	-1.7616	2.25E-11	Ap5b1
ENSMUSG00000031303	-1.7583	1.43E-16	Map3k15
ENSMUSG00000029275	-1.7533	2.59E-05	Gfi1

ENSMUSG00000024968	-1.7519	0.026071	Rcor2
ENSMUSG00000073940	-1.7516	1.22E-06	Hbb-bt
ENSMUSG00000041351	-1.7514	0.000364	Rap1gap
ENSMUSG00000108314	-1.7446	0.000756	Prkcz2
ENSMUSG00000032185	-1.7431	8.05E-17	Carm1
ENSMUSG00000050994	-1.7392	2.07E-08	Adgb
ENSMUSG00000035547	-1.7391	3.04E-07	Capn5
ENSMUSG00000041977	-1.7377	4.80E-20	Arhgef11
ENSMUSG00000019823	-1.7343	3.62E-16	Mical1
ENSMUSG00000029119	-1.7297	3.16E-15	Man2b2
ENSMUSG00000000486	-1.7245	0.015544	1-Sep
ENSMUSG00000020282	-1.7214	0.010758	Rhbdf1
ENSMUSG00000028991	-1.7212	2.25E-26	Mtor
ENSMUSG00000030468	-1.718	2.11E-20	Siglecg
ENSMUSG00000029516	-1.7168	0.001283	Cit
ENSMUSG00000043795	-1.7161	3.23E-12	Prr33
ENSMUSG00000021108	-1.7116	3.55E-06	Prkch
ENSMUSG00000031493	-1.7088	0.022669	Ggn
ENSMUSG00000001588	-1.7031	1.23E-15	Acap1
ENSMUSG00000015337	-1.6996	0.014832	Endog
ENSMUSG00000017390	-1.6988	2.05E-16	Aldoc
ENSMUSG00000034471	-1.6982	8.98E-05	Caskin2
ENSMUSG00000030088	-1.698	5.52E-11	Aldh1l1
ENSMUSG00000042677	-1.6979	1.89E-15	Zc3h12a
ENSMUSG00000032845	-1.697	1.11E-11	Alpk2
ENSMUSG00000039055	-1.694	0.02617	Eme1
ENSMUSG00000042766	-1.6931	6.82E-06	Trim46
ENSMUSG00000052117	-1.6898	8.95E-10	D630039A03Rik
ENSMUSG00000020850	-1.6896	9.35E-22	Prpf8
ENSMUSG00000055809	-1.6874	3.17E-09	Dnaaf3
ENSMUSG00000037239	-1.6802	5.77E-14	Spred3
ENSMUSG00000042328	-1.6798	2.74E-14	Hps4
ENSMUSG00000056399	-1.6792	0.040744	Prss34
ENSMUSG00000057672	-1.6745	2.68E-16	Pkn1
ENSMUSG00000089736	-1.6701	0.002311	Tgfbr3l
ENSMUSG00000035279	-1.6622	0.009037	Ssc5d
ENSMUSG00000059895	-1.662	5.57E-12	Ptp4a3
ENSMUSG00000022758	-1.6583	0.00597	P2rx6
ENSMUSG00000024212	-1.6524	1.98E-17	Mllt1
ENSMUSG00000026135	-1.6521	1.30E-13	Zfp142
ENSMUSG00000001053	-1.6515	1.27E-09	N4bp3
ENSMUSG00000097993	-1.6514	8.25E-05	Ptprv
ENSMUSG00000022443	-1.6501	2.34E-24	Myh9
ENSMUSG00000036526	-1.6486	4.85E-06	Card11
ENSMUSG00000055485	-1.6448	1.80E-17	Soga1
ENSMUSG00000039201	-1.6428	0.00012	Tbc1d25
ENSMUSG00000078922	-1.6376	8.95E-15	Tgtp1
ENSMUSG00000049625	-1.6374	1.68E-10	Tifab
ENSMUSG00000033624	-1.6366	9.46E-14	Pdpr
ENSMUSG00000048031	-1.6365	0.00856	Fcrl5
ENSMUSG00000015647	-1.6346	0.005374	Lama5
ENSMUSG00000029478	-1.6326	7.17E-14	Ncor2
ENSMUSG00000050908	-1.6315	0.019851	Tvp23a

ENSMUSG00000041642	-1.6296	1.82E-21	Kif21b
ENSMUSG00000054150	-1.6259	0.00281	Syne3
ENSMUSG00000041528	-1.6252	4.42E-21	Rnf123
ENSMUSG00000029547	-1.6216	2.19E-16	Ints1
ENSMUSG00000024201	-1.6203	1.21E-18	Kdm4b
ENSMUSG00000047959	-1.6196	0.001112	Kcna3
ENSMUSG00000036587	-1.6188	0.039893	Fut7
ENSMUSG00000029102	-1.6175	0.000371	Hgfac
ENSMUSG00000063382	-1.6159	1.49E-23	Bcl9l
ENSMUSG00000019464	-1.6133	0.00375	Ptger1
ENSMUSG00000047793	-1.6077	0.000298	Sned1
ENSMUSG00000063506	-1.6075	9.34E-06	Arhgap22
ENSMUSG00000006731	-1.6073	5.08E-14	B4galnt1
ENSMUSG00000038644	-1.6044	5.95E-11	Pold1
ENSMUSG00000032855	-1.604	9.65E-23	Pkd1
ENSMUSG00000054640	-1.604	7.73E-08	Slc8a1
ENSMUSG00000027171	-1.6026	8.78E-12	Prrg4
ENSMUSG00000027004	-1.601	0.001635	Frzb
ENSMUSG00000019433	-1.5983	4.09E-12	Gipc1
ENSMUSG00000025017	-1.5966	1.57E-18	Pik3ap1
ENSMUSG00000024855	-1.5941	9.95E-26	Pacs1
ENSMUSG00000013973	-1.594	2.90E-08	Dedd
ENSMUSG00000026121	-1.5931	5.00E-13	Sema4c
ENSMUSG00000006784	-1.5918	0.002129	Ttc25
ENSMUSG00000097331	-1.5872	0.000371	F420014N23Rik
ENSMUSG00000060716	-1.5869	0.002636	Plekhh1
ENSMUSG00000042333	-1.5814	2.74E-14	Tnfrsf14
ENSMUSG00000023938	-1.5809	6.30E-11	Aars2
ENSMUSG00000095427	-1.5803	0.001255	Rps2-ps6
ENSMUSG00000040860	-1.5795	7.95E-08	Crocc
ENSMUSG00000093445	-1.578	0.00016	Lrch4
ENSMUSG00000059323	-1.5774	4.65E-08	Tonsl
ENSMUSG00000042744	-1.5768	9.24E-20	Hectd4
ENSMUSG00000037112	-1.5744	1.75E-11	Sik2
ENSMUSG00000022159	-1.5735	0.000135	Rab2b
ENSMUSG00000028703	-1.5721	7.72E-15	Lrrc41
ENSMUSG00000035640	-1.5674	6.48E-08	Cbarp
ENSMUSG00000015134	-1.5652	0.047467	Aldh1a3
ENSMUSG00000053063	-1.5648	6.16E-05	Clec12a
ENSMUSG00000026860	-1.563	2.71E-11	Sh3glb2
ENSMUSG00000036622	-1.5498	3.52E-09	Atp13a2
ENSMUSG00000015083	-1.5497	0.003402	C8g
ENSMUSG00000029165	-1.5493	1.69E-09	Agbl5
ENSMUSG00000022090	-1.5478	5.55E-06	Pdlim2
ENSMUSG00000021287	-1.5478	8.70E-10	Xrcc3
ENSMUSG00000029101	-1.5474	1.96E-13	Rgs12
ENSMUSG00000027324	-1.5455	6.48E-05	Rpusd2
ENSMUSG00000070000	-1.5439	1.13E-16	Fcho1
ENSMUSG00000031955	-1.5434	4.61E-06	Bcar1
ENSMUSG00000033434	-1.542	1.61E-06	Gtpbp6
ENSMUSG00000057666	-1.5415	0.000114	Gapdh
ENSMUSG00000038797	-1.5409	0.000172	Zscan2
ENSMUSG00000026688	-1.5408	5.16E-05	Mgst3

ENSMUSG00000030588	-1.5408	3.50E-12	Yif1b
ENSMUSG00000048732	-1.5405	3.46E-05	Klhl11
ENSMUSG00000033124	-1.54	3.03E-11	Atg9a
ENSMUSG00000024334	-1.5357	6.59E-11	H2-Oa
ENSMUSG00000020277	-1.5353	3.48E-16	Pfkl
ENSMUSG00000052609	-1.5348	1.45E-11	Plekhg3
ENSMUSG00000003660	-1.5334	2.77E-24	Snrnp200
ENSMUSG00000047417	-1.5294	9.25E-13	Rexo1
ENSMUSG00000069830	-1.5241	0.019121	Nlrp1a
ENSMUSG00000076435	-1.523	9.85E-08	Acsf2
ENSMUSG00000070524	-1.5226	1.05E-05	Fcrlb
ENSMUSG00000005057	-1.5203	1.85E-10	Sh2b2
ENSMUSG00000054874	-1.5193	1.59E-10	Pcnx3
ENSMUSG00000097636	-1.5186	2.56E-07	Mirt1
ENSMUSG00000032777	-1.5165	2.77E-19	Gtf3c1
ENSMUSG00000055725	-1.5155	4.57E-06	Paqr3
ENSMUSG00000021367	-1.5095	3.86E-18	Edn1
ENSMUSG00000075600	-1.5095	2.65E-06	Zc3h3
ENSMUSG00000022212	-1.5081	0.001588	Cpne6
ENSMUSG00000022565	-1.5071	4.98E-16	Plec
ENSMUSG00000020238	-1.5022	2.70E-07	Ncln
ENSMUSG00000029033	-1.5004	5.66E-12	Acap3
ENSMUSG00000059040	-1.5	5.72E-12	Eno1b
ENSMUSG00000061684	-1.4988	1.01E-09	Rpl21-ps8
ENSMUSG00000020439	-1.4986	0.0286	Smtn
ENSMUSG00000024019	-1.4971	3.71E-05	Cmtr1
ENSMUSG00000035206	-1.4952	3.77E-10	Sppl2b
ENSMUSG00000027889	-1.4945	7.17E-14	Ampd2
ENSMUSG00000004788	-1.494	1.38E-12	Eif2b2
ENSMUSG00000026842	-1.4929	8.98E-15	Abl1
ENSMUSG00000028917	-1.4902	3.54E-16	Plekhm2
ENSMUSG00000036606	-1.4881	2.49E-07	Plxnb2
ENSMUSG00000061306	-1.4859	1.90E-17	Slc38a10
ENSMUSG00000022574	-1.4802	0.002972	Naprt
ENSMUSG00000027546	-1.477	0.012607	Atp9a
ENSMUSG00000021280	-1.477	0.002109	Exoc3l4
ENSMUSG00000037992	-1.4765	2.21E-11	Rara
ENSMUSG00000040734	-1.4755	0.000226	Ppp1r13l
ENSMUSG00000079014	-1.4712	5.76E-05	Serpina3i
ENSMUSG00000071477	-1.4693	1.00E-08	Zfp777
ENSMUSG00000042644	-1.4676	1.12E-12	Itpr3
ENSMUSG00000052821	-1.4655	2.13E-14	Cysltr1
ENSMUSG00000024327	-1.4626	0.014918	Slc39a7
ENSMUSG00000043099	-1.4616	8.18E-06	Hic1
ENSMUSG00000023908	-1.4616	0.004867	Pkmyt1
ENSMUSG00000029599	-1.4612	2.37E-19	Ddx54
ENSMUSG00000021061	-1.4611	0.026608	Sptb
ENSMUSG00000051980	-1.4602	0.008687	Casr
ENSMUSG00000034522	-1.4601	1.84E-09	Zfp395
ENSMUSG00000021819	-1.459	3.50E-14	Zswim8
ENSMUSG00000024163	-1.4584	7.20E-16	Mapk8ip3
ENSMUSG00000024870	-1.4575	1.43E-21	Rab1b
ENSMUSG00000039699	-1.4568	5.51E-06	Batf2

ENSMUSG00000001552	-1.4567	1.01E-08	Jup
ENSMUSG00000022096	-1.4563	0.014603	Hr
ENSMUSG00000078789	-1.4557	0.001351	Dph1
ENSMUSG00000000631	-1.4555	3.24E-20	Myo18a
ENSMUSG00000024160	-1.4516	0.000375	Spsb3
ENSMUSG00000004562	-1.4496	1.62E-13	Arhgef40
ENSMUSG00000029500	-1.4493	4.04E-08	Pgam5
ENSMUSG00000070604	-1.449	0.023469	Vsig10l
ENSMUSG00000002409	-1.4464	2.54E-08	Dyrk1b
ENSMUSG00000028465	-1.4446	4.66E-21	Tln1
ENSMUSG00000027962	-1.4446	3.19E-10	Vcam1
ENSMUSG00000025384	-1.4427	1.48E-16	Faap100
ENSMUSG00000034445	-1.4422	5.38E-11	Cyb561a3
ENSMUSG00000034165	-1.4418	7.08E-23	Ccnd3
ENSMUSG00000027950	-1.4415	5.72E-07	Chrnb2
ENSMUSG00000059149	-1.4415	5.32E-10	Mfsd4a
ENSMUSG00000023118	-1.4408	3.54E-12	Sympk
ENSMUSG00000091957	-1.4381	0.007278	Rps2-ps10
ENSMUSG00000006019	-1.4354	2.70E-10	Dhx34
ENSMUSG00000025226	-1.435	0.017353	Fbxl15
ENSMUSG00000052533	-1.4342	1.21E-10	Nup188
ENSMUSG00000019578	-1.4334	7.46E-07	Ubxn6
ENSMUSG00000031834	-1.4329	4.55E-16	Pik3r2
ENSMUSG00000020747	-1.4312	1.90E-13	Tmem94
ENSMUSG00000037579	-1.4284	5.94E-05	Kcnh3
ENSMUSG00000027457	-1.4281	7.55E-06	Snph
ENSMUSG00000063146	-1.4279	3.20E-14	Clip2
ENSMUSG00000021866	-1.4278	2.43E-12	Anxa11
ENSMUSG00000033249	-1.4268	0.009014	Hsf4
ENSMUSG00000019139	-1.4255	1.26E-08	Isyna1
ENSMUSG00000013629	-1.4252	1.81E-06	Cad
ENSMUSG00000029345	-1.4252	6.88E-15	Tfip11
ENSMUSG00000014426	-1.4251	7.39E-13	Map3k4
ENSMUSG00000004054	-1.4249	1.69E-09	Map3k11
ENSMUSG00000038623	-1.4248	3.77E-10	Tm6sf1
ENSMUSG00000046169	-1.4244	7.14E-05	Adamts6
ENSMUSG00000025736	-1.4237	0.000421	Jmjd8
ENSMUSG00000064390	-1.4236	0.015302	Rnu73b
ENSMUSG00000027670	-1.4219	1.85E-11	Ocstamp
ENSMUSG00000029417	-1.4208	0.000281	Cxcl9
ENSMUSG00000079575	-1.4131	2.91E-07	Rbpj-ps3
ENSMUSG00000005465	-1.412	0.03716	Il27ra
ENSMUSG00000031293	-1.4118	0.005213	Rs1
ENSMUSG00000062115	-1.4109	5.17E-16	Rai1
ENSMUSG00000031865	-1.4088	2.62E-15	Dctn1
ENSMUSG00000032295	-1.4081	3.38E-12	Man2c1
ENSMUSG00000035498	-1.408	8.81E-16	Cdcp1
ENSMUSG00000060279	-1.4079	3.09E-13	Ap2a1
ENSMUSG00000059013	-1.4079	0.015645	Sh2d3c
ENSMUSG00000030718	-1.4068	2.65E-16	Ppme1
ENSMUSG00000052142	-1.4052	5.92E-06	Rasal3
ENSMUSG00000040857	-1.4048	1.39E-05	Erf
ENSMUSG00000047746	-1.4043	1.17E-06	Fbxo40

ENSMUSG00000034156	-1.404	8.35E-13	Tspoap1
ENSMUSG00000041782	-1.4026	2.46E-19	Lad1
ENSMUSG00000027582	-1.4008	8.71E-12	Zgpat
ENSMUSG00000031832	-1.4005	1.72E-06	Taf1c
ENSMUSG00000004931	-1.3994	1.52E-08	Apba3
ENSMUSG00000003435	-1.3956	1.29E-16	Supt5
ENSMUSG00000031958	-1.3954	0.044439	Ldhd
ENSMUSG00000050106	-1.3927	5.84E-05	Tmc8
ENSMUSG00000014786	-1.3905	0.000128	Slc9a5
ENSMUSG00000033396	-1.39	3.37E-21	Spg11
ENSMUSG00000040963	-1.3884	8.89E-09	Asgr2
ENSMUSG00000006675	-1.3861	0.002819	P4htm
ENSMUSG00000042594	-1.3852	9.02E-13	Sh2b3
ENSMUSG00000070420	-1.3838	9.02E-10	Zscan25
ENSMUSG00000039787	-1.3825	8.46E-10	Cercam
ENSMUSG00000032867	-1.3804	2.11E-09	Fbxw8
ENSMUSG00000000693	-1.3758	1.56E-05	Loxl3
ENSMUSG00000046971	-1.3753	0.000737	Pla2g4f
ENSMUSG00000030780	-1.3752	0.000413	BC017158
ENSMUSG00000039813	-1.3747	3.15E-06	Tbc1d2
ENSMUSG00000014498	-1.3746	2.77E-18	Ankrd52
ENSMUSG00000005447	-1.3739	1.48E-10	Pafah1b3
ENSMUSG00000050377	-1.3723	1.06E-15	Il31ra
ENSMUSG00000045691	-1.3699	0.018603	Thtpa
ENSMUSG00000002835	-1.3698	7.33E-06	Chaf1a
ENSMUSG00000025792	-1.3696	4.87E-15	Slc25a10
ENSMUSG00000003153	-1.3688	1.59E-07	Slc2a3
ENSMUSG00000054808	-1.3686	3.04E-25	Actn4
ENSMUSG00000011877	-1.367	2.12E-13	Git1
ENSMUSG00000074272	-1.3668	7.24E-06	Ceacam1
ENSMUSG00000060601	-1.3663	0.002411	Nr1h2
ENSMUSG00000023571	-1.3654	1.39E-11	C1qtnf12
ENSMUSG00000029299	-1.3652	0.007812	Abcg3
ENSMUSG00000033792	-1.3616	3.11E-14	Atp7a
ENSMUSG00000003200	-1.3615	1.90E-12	Sh3gl1
ENSMUSG00000042535	-1.3601	5.21E-16	Gtpbp1
ENSMUSG00000047810	-1.36	1.14E-13	Ccdc88b
ENSMUSG00000020782	-1.36	2.61E-05	Llgl2
ENSMUSG00000026276	-1.3595	1.17E-08	2-Sep
ENSMUSG00000055675	-1.3591	3.95E-05	Kbtbd11
ENSMUSG00000042810	-1.3567	2.29E-05	Krba1
ENSMUSG00000090958	-1.3554	2.55E-14	Lrrc32
ENSMUSG00000030530	-1.355	2.49E-14	Furin
ENSMUSG00000024393	-1.3542	7.03E-18	Prrc2a
ENSMUSG00000036854	-1.3515	0.017815	Hspb6
ENSMUSG00000024851	-1.3491	1.54E-12	Pitpnm1
ENSMUSG00000037447	-1.3483	9.46E-14	Arid5a
ENSMUSG00000040260	-1.3469	0.005696	Daam2
ENSMUSG00000060376	-1.3458	0.000264	Bckdha
ENSMUSG00000032558	-1.3457	4.12E-11	Nphp3
ENSMUSG00000106755	-1.3449	0.016848	Tpi-rs11
ENSMUSG00000025701	-1.3424	4.77E-16	Alox5
ENSMUSG00000025262	-1.3421	0.007803	Fam120c

ENSMUSG00000038181	-1.3418	4.54E-13	Chpf2
ENSMUSG00000022504	-1.3417	2.91E-20	Ciita
ENSMUSG00000042292	-1.3415	3.04E-15	Mkl1
ENSMUSG00000089942	-1.3407	4.02E-06	Pira2
ENSMUSG00000034758	-1.3406	2.51E-07	Tle6
ENSMUSG00000036281	-1.3405	1.92E-07	Snapc4
ENSMUSG00000024018	-1.3403	8.13E-08	Ccdc167
ENSMUSG00000034902	-1.3401	5.61E-15	Pip5k1c
ENSMUSG00000035890	-1.3393	1.12E-12	Rnf126
ENSMUSG00000020882	-1.3387	4.41E-08	Cacnb1
ENSMUSG00000032312	-1.3386	3.92E-13	Csk
ENSMUSG00000072294	-1.3386	0.006571	Klf12
ENSMUSG00000037337	-1.3384	3.53E-11	Map4k1
ENSMUSG00000002885	-1.3356	1.39E-12	Adgre5
ENSMUSG00000082286	-1.3351	2.96E-06	Pisd-ps1
ENSMUSG00000039891	-1.3331	3.30E-05	Txlnb
ENSMUSG00000039686	-1.3328	4.11E-10	Zer1
ENSMUSG00000074283	-1.3322	0.004926	Zfp109
ENSMUSG00000003644	-1.3318	6.89E-15	Rps6ka1
ENSMUSG00000038233	-1.3298	0.005053	Fam198a
ENSMUSG00000028671	-1.3298	2.02E-08	Gale
ENSMUSG00000038967	-1.3296	1.08E-05	Pdk2
ENSMUSG00000021221	-1.3294	0.009189	Dpf3
ENSMUSG00000032637	-1.3291	1.06E-12	Atxn2l
ENSMUSG00000024251	-1.3289	6.92E-14	Thada
ENSMUSG00000039308	-1.3285	1.46E-13	Ndst2
ENSMUSG00000074899	-1.3274	0.000977	Sptbn5
ENSMUSG00000026879	-1.3271	9.84E-15	Gsn
ENSMUSG00000022372	-1.3247	4.59E-15	Sla
ENSMUSG00000023008	-1.3243	1.04E-08	Fmnl3
ENSMUSG00000025870	-1.3235	2.46E-05	Arl10
ENSMUSG00000028100	-1.3232	1.10E-09	Nudt17
ENSMUSG00000028068	-1.3225	0.016595	Iqgap3
ENSMUSG00000039853	-1.3207	1.11E-09	Trim14
ENSMUSG00000027951	-1.3193	1.35E-14	Adar
ENSMUSG00000020211	-1.3181	1.60E-09	Sf3a2
ENSMUSG00000022223	-1.3176	0.014011	Sdr39u1
ENSMUSG00000031295	-1.317	2.22E-12	Phka2
ENSMUSG00000029674	-1.3168	1.01E-06	Limk1
ENSMUSG00000033253	-1.3165	4.41E-12	Szt2
ENSMUSG00000074570	-1.3159	2.48E-08	Cass4
ENSMUSG00000004933	-1.3147	0.027232	Matk
ENSMUSG00000017670	-1.3135	9.60E-15	Elmo2
ENSMUSG00000102418	-1.3122	0.001258	Sh2d1b1
ENSMUSG00000038252	-1.3117	2.88E-08	Ncapd2
ENSMUSG00000010066	-1.3113	0.004577	Cacna2d2
ENSMUSG00000034949	-1.3108	7.61E-06	Zfr2
ENSMUSG00000044092	-1.31	6.65E-10	C130050O18Rik
ENSMUSG00000046574	-1.3091	3.41E-11	Prr12
ENSMUSG00000006342	-1.3083	0.004215	Susd2
ENSMUSG00000075254	-1.3058	0.000623	Heg1
ENSMUSG00000046245	-1.3058	2.07E-07	Pilra
ENSMUSG00000078552	-1.3053	0.008319	Dcdc2b

ENSMUSG00000000182	-1.3053	7.15E-08	Fgf23
ENSMUSG00000026941	-1.3053	0.040389	Mamdc4
ENSMUSG00000032898	-1.305	4.21E-16	Fbxo21
ENSMUSG00000009292	-1.305	0.002691	Trpm2
ENSMUSG00000022442	-1.3037	5.56E-06	Ttll1
ENSMUSG00000026858	-1.3031	0.000336	Miga2
ENSMUSG00000015659	-1.3026	1.39E-05	Serac1
ENSMUSG00000034255	-1.3024	4.30E-10	Arhgap27
ENSMUSG00000021144	-1.3016	1.87E-18	Mta1
ENSMUSG00000025743	-1.301	3.53E-12	Sdc3
ENSMUSG00000027463	-1.2992	3.54E-18	Slc52a3
ENSMUSG00000026170	-1.299	0.001102	Cyp27a1
ENSMUSG00000062075	-1.2989	2.55E-06	Lmnb2
ENSMUSG00000019261	-1.2988	2.80E-11	Map1s
ENSMUSG00000033128	-1.2984	2.39E-12	Gga1
ENSMUSG00000011752	-1.2953	5.04E-13	Pgam1
ENSMUSG00000038637	-1.2921	9.42E-08	Lrrc56
ENSMUSG00000013833	-1.292	3.53E-08	Med16
ENSMUSG00000036529	-1.2891	1.04E-10	Sbf1
ENSMUSG00000039637	-1.2868	1.36E-12	Coro7
ENSMUSG00000025494	-1.2868	5.64E-07	Sigirr
ENSMUSG00000043671	-1.2858	5.21E-10	Dpy19l3
ENSMUSG00000002550	-1.2857	8.27E-07	Uck1
ENSMUSG00000025509	-1.2847	3.78E-08	Pnpla2
ENSMUSG00000070003	-1.2809	3.78E-05	Ssbp4
ENSMUSG00000038775	-1.2801	0.000201	Vill
ENSMUSG00000028128	-1.2799	5.71E-05	F3
ENSMUSG00000024942	-1.2793	2.08E-13	Capn1
ENSMUSG00000035198	-1.2789	0.000193	Tubg1
ENSMUSG00000029001	-1.2788	0.001071	Fbxo44
ENSMUSG00000039646	-1.2764	6.39E-09	Vasn
ENSMUSG00000004099	-1.2758	1.87E-17	Dnmt1
ENSMUSG00000051786	-1.2725	4.37E-08	Tubgcp6
ENSMUSG00000045039	-1.2721	1.47E-11	Megf8
ENSMUSG00000061859	-1.2704	0.031139	Patj
ENSMUSG00000037411	-1.2699	8.66E-18	Serpine1
ENSMUSG00000030137	-1.2697	2.34E-05	Tuba8
ENSMUSG00000033594	-1.2682	2.45E-06	Spata2l
ENSMUSG00000018909	-1.2681	1.16E-18	Arrb1
ENSMUSG00000034951	-1.2666	5.59E-12	Cog7
ENSMUSG00000028137	-1.2666	0.003055	Celf3
ENSMUSG00000020393	-1.2655	6.80E-16	Kremen1
ENSMUSG00000044026	-1.2655	0.010671	Slc35g1
ENSMUSG00000009090	-1.2651	8.95E-15	Ap1b1
ENSMUSG00000040524	-1.2649	6.36E-17	Zfp609
ENSMUSG00000036686	-1.2641	2.59E-09	Cc2d1a
ENSMUSG00000030091	-1.264	5.56E-16	Nup210
ENSMUSG00000039976	-1.2635	7.72E-06	Tbc1d16
ENSMUSG00000034330	-1.2634	1.20E-10	Plcg2
ENSMUSG00000040247	-1.2633	1.86E-06	Tbc1d10c
ENSMUSG00000024442	-1.2625	2.97E-08	Dele1
ENSMUSG00000029638	-1.2623	3.50E-05	Glcci1
ENSMUSG00000029127	-1.2605	7.13E-05	Zbtb49

ENSMUSG00000027544	-1.2599	1.01E-10	Nfatc2
ENSMUSG00000055745	-1.2589	0.00743	Rtl6
ENSMUSG00000070808	-1.2584	1.76E-09	Bicra
ENSMUSG00000041679	-1.2568	0.013942	Lrrc29
ENSMUSG00000020697	-1.2568	1.49E-06	Lig3
ENSMUSG00000094441	-1.2561	0.000324	Zfp955a
ENSMUSG00000040276	-1.2547	0.000872	Pacsin1
ENSMUSG00000040327	-1.2542	1.90E-09	Cul9
ENSMUSG00000050592	-1.2524	0.001645	Fam78a
ENSMUSG00000018008	-1.2516	3.61E-13	Cyth4
ENSMUSG00000027580	-1.2515	1.34E-12	Helz2
ENSMUSG00000006307	-1.25	1.59E-14	Kmt2b
ENSMUSG00000032512	-1.2496	1.46E-14	Wdr48
ENSMUSG00000026116	-1.2486	6.58E-22	Tmem131
ENSMUSG00000025366	-1.2478	9.00E-15	Esyt1
ENSMUSG00000079657	-1.2478	0.003526	Rab26
ENSMUSG00000055235	-1.2462	0.00322	Wdr86
ENSMUSG00000020196	-1.2461	3.94E-09	Cabin1
ENSMUSG00000023004	-1.2451	5.95E-25	Tuba1b
ENSMUSG00000024965	-1.2441	1.31E-10	Fermt3
ENSMUSG00000013275	-1.2438	3.81E-15	Slc41a1
ENSMUSG00000024298	-1.2394	6.38E-15	Zfp871
ENSMUSG00000043467	-1.2379	7.60E-15	Zbtb37
ENSMUSG00000041638	-1.2367	1.31E-15	Gcn1l1
ENSMUSG00000031389	-1.2361	4.76E-11	Arhgap4
ENSMUSG00000025161	-1.2356	0.000708	Slc16a3
ENSMUSG00000034269	-1.2316	1.95E-28	Setd5
ENSMUSG00000005442	-1.231	1.01E-13	Cic
ENSMUSG00000029104	-1.2306	1.21E-26	Htt
ENSMUSG00000039738	-1.2303	2.69E-05	Slx4
ENSMUSG00000060591	-1.2297	1.18E-11	Ifitm2
ENSMUSG00000019214	-1.2296	0.011633	Chtf18
ENSMUSG00000037408	-1.2295	6.56E-05	Cnnm4
ENSMUSG00000012443	-1.2294	0.041274	Kif11
ENSMUSG00000028931	-1.2291	1.12E-12	Kcnab2
ENSMUSG00000026814	-1.2275	3.40E-07	Eng
ENSMUSG00000032737	-1.2274	4.66E-11	Inppl1
ENSMUSG00000032092	-1.2267	0.037644	Mpzl2
ENSMUSG00000039981	-1.2266	0.000145	Zc3h12d
ENSMUSG00000056515	-1.2264	1.16E-10	Rab31
ENSMUSG00000057788	-1.2257	2.76E-08	Ddx49
ENSMUSG00000072571	-1.2249	0.0456	Tmem253
ENSMUSG00000030600	-1.2246	0.037131	Lrfn1
ENSMUSG00000049739	-1.2243	1.23E-10	Zfp646
ENSMUSG00000038517	-1.224	0.001672	Tbkbp1
ENSMUSG00000051043	-1.2232	0.001947	Gprc5c
ENSMUSG00000031391	-1.221	2.61E-24	L1cam
ENSMUSG00000006471	-1.2206	7.56E-06	Ndor1
ENSMUSG00000013707	-1.2206	4.64E-07	Tnfaip8l2
ENSMUSG00000042632	-1.2202	8.78E-05	Pla2g6
ENSMUSG00000011114	-1.22	8.99E-13	Tbrg1
ENSMUSG00000029322	-1.2195	0.005339	Plac8
ENSMUSG00000000958	-1.2194	5.26E-10	Slc7a7

ENSMUSG00000060261	-1.2182	1.57E-09	Gtf2i
ENSMUSG00000026580	-1.217	3.01E-05	Selp
ENSMUSG00000033335	-1.2165	3.22E-15	Dnm2
ENSMUSG00000052013	-1.2163	4.07E-10	Btla
ENSMUSG00000023079	-1.2144	4.87E-07	Gtf2ird1
ENSMUSG00000015968	-1.2133	9.83E-11	Cacna1d
ENSMUSG00000021265	-1.2128	0.001883	Slc25a29
ENSMUSG00000040385	-1.2124	2.79E-11	Ppp1ca
ENSMUSG00000017607	-1.2117	2.26E-06	Tns4
ENSMUSG00000022475	-1.2113	9.89E-09	Hdac7
ENSMUSG00000042349	-1.2101	9.80E-17	Ikbke
ENSMUSG00000036980	-1.2094	6.56E-06	Taf6
ENSMUSG00000026792	-1.2072	2.87E-07	Lrsam1
ENSMUSG00000054199	-1.2065	2.82E-07	Gon4l
ENSMUSG00000057530	-1.2057	1.22E-13	Ece1
ENSMUSG00000036565	-1.2056	5.33E-14	Ttyh3
ENSMUSG00000057337	-1.2044	0.003619	Chst3
ENSMUSG00000028862	-1.2041	0.005829	Map3k6
ENSMUSG00000001855	-1.2041	2.08E-13	Nup214
ENSMUSG00000020766	-1.2003	7.60E-05	Galk1
ENSMUSG00000033307	-1.1998	3.77E-10	Mif
ENSMUSG00000025737	-1.199	3.60E-08	Wdr24
ENSMUSG00000000791	-1.1984	0.014379	Il12rb1
ENSMUSG00000003378	-1.1968	8.80E-05	Grik5
ENSMUSG00000041757	-1.1966	0.011377	Plekha6
ENSMUSG00000005198	-1.1963	5.25E-22	Polr2a
ENSMUSG00000041477	-1.1959	1.85E-05	Dcp1b
ENSMUSG00000050395	-1.1955	7.04E-15	Tnfsf15
ENSMUSG00000054452	-1.1953	3.27E-11	Aes
ENSMUSG00000034616	-1.1945	4.29E-08	Ssh3
ENSMUSG00000047090	-1.1936	1.96E-05	Tmem198b
ENSMUSG00000024050	-1.1919	6.59E-10	Wiz
ENSMUSG00000030400	-1.1918	7.78E-06	Ercc2
ENSMUSG00000002781	-1.1907	1.53E-06	Tmem143
ENSMUSG00000057229	-1.1893	5.91E-10	Dmac2
ENSMUSG00000028803	-1.189	6.04E-09	Nipal3
ENSMUSG00000002870	-1.1887	2.50E-06	Mcm2
ENSMUSG00000040158	-1.1886	2.12E-08	Tax1bp3
ENSMUSG00000038521	-1.1885	0.003394	C1s1
ENSMUSG00000034032	-1.1885	4.29E-09	Rpap1
ENSMUSG00000061815	-1.185	0.001276	Rufy4
ENSMUSG00000053877	-1.1832	7.46E-05	Srcap
ENSMUSG00000037003	-1.1827	0.015219	Tns2
ENSMUSG00000002496	-1.1827	1.69E-13	Tsc2
ENSMUSG00000033287	-1.1824	1.27E-08	Kctd17
ENSMUSG00000000916	-1.1823	0.000217	Nsun5
ENSMUSG00000068663	-1.1818	1.49E-17	Clec16a
ENSMUSG00000036644	-1.1817	2.70E-18	Tbc1d9b
ENSMUSG00000031328	-1.1807	1.73E-12	Flna
ENSMUSG00000011267	-1.1802	8.06E-06	Zfp296
ENSMUSG00000020471	-1.1797	1.36E-05	Pold2
ENSMUSG00000024164	-1.1789	1.07E-20	C3
ENSMUSG00000038241	-1.178	7.85E-14	Cep250

ENSMUSG00000055805	-1.1773	3.89E-09	Fmnl1
ENSMUSG00000071647	-1.1761	3.84E-11	Eml3
ENSMUSG00000029610	-1.1757	1.59E-05	Aimp2
ENSMUSG00000037161	-1.1743	6.95E-08	Mgarp
ENSMUSG00000031778	-1.1742	1.20E-10	Cx3cl1
ENSMUSG00000049086	-1.1739	9.83E-05	Bmyc
ENSMUSG00000044134	-1.1735	1.10E-05	Pheta1
ENSMUSG00000001473	-1.1734	4.10E-14	Tubb6
ENSMUSG00000001910	-1.1728	6.58E-10	Nacc1
ENSMUSG00000023991	-1.1727	8.54E-07	Foxp4
ENSMUSG00000003526	-1.1723	2.57E-05	Prodh
ENSMUSG00000032514	-1.171	9.92E-07	Ttc21a
ENSMUSG00000025132	-1.1707	2.57E-11	Arhgdia
ENSMUSG00000039218	-1.169	2.98E-19	Srrm2
ENSMUSG00000038527	-1.1686	0.005341	C1rl
ENSMUSG00000031828	-1.1669	0.000216	Klhl36
ENSMUSG00000057738	-1.1668	6.47E-19	Sptan1
ENSMUSG00000054434	-1.1664	2.79E-07	Tmem120b
ENSMUSG00000031103	-1.1653	4.21E-13	Elf4
ENSMUSG00000086533	-1.1647	0.007373	Mypopos
ENSMUSG00000056492	-1.1646	0.001709	Adgrf5
ENSMUSG00000073758	-1.1634	8.99E-05	Sh3d21
ENSMUSG00000027933	-1.1625	3.51E-14	Ints3
ENSMUSG00000068220	-1.1621	1.61E-10	Lgals1
ENSMUSG00000074269	-1.1618	0.023755	Rec114
ENSMUSG00000022584	-1.1616	6.82E-07	Ly6c2
ENSMUSG00000000732	-1.1607	4.37E-06	Icosl
ENSMUSG00000039205	-1.1601	5.66E-10	Ciz1
ENSMUSG00000034371	-1.1596	1.98E-06	Tkfc
ENSMUSG00000030347	-1.1594	2.46E-07	D6Wsu163e
ENSMUSG00000028479	-1.1589	1.04E-08	Gne
ENSMUSG00000029402	-1.1583	0.000164	Snrnp35
ENSMUSG00000041037	-1.1573	2.68E-08	Irgq
ENSMUSG00000062300	-1.1572	2.77E-09	Nectin2
ENSMUSG00000027377	-1.1558	0.001243	Mall
ENSMUSG00000052949	-1.1557	0.000164	Rnf157
ENSMUSG00000038545	-1.1547	1.32E-09	Cul7
ENSMUSG00000017307	-1.1534	1.49E-08	Acot8
ENSMUSG00000054435	-1.1532	0.013807	Gimap4
ENSMUSG00000059602	-1.1532	0.000191	Syn3
ENSMUSG00000034390	-1.1526	9.89E-15	Cmip
ENSMUSG00000043572	-1.1526	0.004258	Pars2
ENSMUSG00000043505	-1.1522	9.31E-07	Gimap5
ENSMUSG00000020827	-1.1521	1.64E-09	Mink1
ENSMUSG00000025437	-1.1517	3.11E-09	Usp33
ENSMUSG00000024560	-1.1516	4.39E-10	Cxxc1
ENSMUSG00000061046	-1.1496	5.21E-06	Haghl
ENSMUSG00000022571	-1.1492	0.000151	Pycrl
ENSMUSG00000050721	-1.1464	2.38E-15	Plekho2
ENSMUSG00000023143	-1.1463	5.30E-05	Nagpa
ENSMUSG00000035697	-1.1458	1.61E-10	Arhgap45
ENSMUSG00000022568	-1.1441	1.13E-08	Scrib
ENSMUSG00000006641	-1.1429	0.012566	Slc5a6

ENSMUSG00000027379	-1.1427	0.019017	Bub1
ENSMUSG00000031879	-1.1423	2.92E-06	Fam96b
ENSMUSG00000025188	-1.1418	1.34E-07	Hps1
ENSMUSG00000030533	-1.1414	9.74E-10	Unc45a
ENSMUSG00000044452	-1.1406	0.019239	Zfp507
ENSMUSG00000073434	-1.14	2.30E-07	Wdr90
ENSMUSG00000021196	-1.1395	5.11E-19	Pfkp
ENSMUSG00000001865	-1.1391	0.036442	Cpa3
ENSMUSG00000008855	-1.1386	2.14E-05	Hdac5
ENSMUSG00000047284	-1.1372	7.56E-11	Neurl4
ENSMUSG00000052423	-1.1372	1.78E-05	B4galt3
ENSMUSG00000052160	-1.1366	2.24E-06	Pld4
ENSMUSG00000030427	-1.1365	3.54E-08	Lilra6
ENSMUSG00000107476	-1.1365	3.70E-06	Zfp862-ps
ENSMUSG00000005580	-1.1362	0.004361	Adcy9
ENSMUSG00000025473	-1.1356	1.46E-12	Adam8
ENSMUSG00000035637	-1.1352	0.000584	Grhpr
ENSMUSG00000033706	-1.1352	0.000187	Smyd5
ENSMUSG00000022120	-1.1346	6.32E-08	Rnf219
ENSMUSG00000095690	-1.1345	0.001028	Rab11b-ps2
ENSMUSG00000029576	-1.1335	4.06E-07	Radil
ENSMUSG00000020941	-1.1332	1.76E-11	Map3k14
ENSMUSG00000061589	-1.1314	1.69E-12	Dot1l
ENSMUSG00000020700	-1.1301	7.15E-15	Map3k3
ENSMUSG00000019370	-1.1299	4.59E-16	Calm3
ENSMUSG00000068699	-1.1283	7.48E-07	Flnc
ENSMUSG00000003282	-1.1283	2.74E-10	Plag1
ENSMUSG00000031858	-1.1282	1.89E-15	Mau2
ENSMUSG00000042700	-1.1277	8.72E-09	Sipa1l1
ENSMUSG00000002043	-1.1277	2.25E-06	Trappc6a
ENSMUSG00000037020	-1.1277	0.009966	Wdr62
ENSMUSG00000035495	-1.1272	6.14E-14	Tstd2
ENSMUSG00000040712	-1.1271	6.07E-13	Camta2
ENSMUSG00000022102	-1.1258	1.18E-10	Dok2
ENSMUSG00000079334	-1.1258	1.60E-05	Nat6
ENSMUSG00000029095	-1.1254	0.019682	Ablim2
ENSMUSG00000027134	-1.1243	8.04E-11	Lpcat4
ENSMUSG00000029072	-1.1243	0.047841	Tas1r3
ENSMUSG00000027579	-1.1235	0.001149	Srms
ENSMUSG00000000056	-1.1223	6.36E-13	Narf
ENSMUSG00000043432	-1.1222	0.007885	Leng9
ENSMUSG00000025579	-1.1216	1.17E-09	Gaa
ENSMUSG00000034748	-1.1207	5.93E-07	Sirt6
ENSMUSG00000015016	-1.1199	2.99E-06	Acsf3
ENSMUSG00000020917	-1.1173	1.74E-17	Acly
ENSMUSG00000040390	-1.1164	3.93E-05	Map3k10
ENSMUSG00000103821	-1.1158	0.020831	D430013B06Rik
ENSMUSG00000004558	-1.1156	1.40E-09	Ndrg2
ENSMUSG00000037326	-1.1151	2.70E-08	Capn15
ENSMUSG00000032562	-1.1148	1.06E-13	Gnai2
ENSMUSG00000046861	-1.1141	1.71E-10	Hectd3
ENSMUSG00000033216	-1.1135	6.50E-05	Eefsec
ENSMUSG00000037211	-1.1131	0.029462	Spry1

ENSMUSG00000048191	-1.1129	0.010249	Muc6
ENSMUSG00000003865	-1.1127	9.48E-12	Gys1
ENSMUSG00000050640	-1.1117	2.85E-06	Tmem150c
ENSMUSG00000039262	-1.1098	6.21E-13	Prrc2b
ENSMUSG00000030707	-1.109	1.83E-10	Coro1a
ENSMUSG00000003868	-1.1083	5.70E-05	Ruvbl2
ENSMUSG00000041199	-1.1078	0.020465	Rpusd1
ENSMUSG00000012819	-1.1072	7.87E-07	Cdh23
ENSMUSG00000040479	-1.106	2.74E-05	Dgkz
ENSMUSG00000010936	-1.1051	1.52E-15	Vac14
ENSMUSG00000035722	-1.1049	5.02E-10	Abca7
ENSMUSG00000034614	-1.1046	9.01E-05	Pik3ip1
ENSMUSG00000026068	-1.104	0.000455	Il18rap
ENSMUSG00000046947	-1.1039	9.10E-07	Adck2
ENSMUSG00000014164	-1.1033	4.36E-05	Klhl3
ENSMUSG00000038524	-1.1018	9.96E-10	Fchsd1
ENSMUSG00000116138	-1.1018	0.005513	C030006K11Rik
ENSMUSG00000028359	-1.1013	0.008	Orm3
ENSMUSG00000049588	-1.1011	0.034666	Ccdc69
ENSMUSG00000042404	-1.1009	6.66E-19	Dennd4b
ENSMUSG00000029723	-1.1007	9.28E-12	Tsc22d4
ENSMUSG00000018474	-1.0999	5.99E-06	Chd3
ENSMUSG00000051557	-1.0997	0.007358	Pusl1
ENSMUSG00000024219	-1.0989	1.76E-13	Anks1
ENSMUSG00000033439	-1.098	2.26E-05	Trmt13
ENSMUSG00000032842	-1.0979	0.000326	Abcc10
ENSMUSG00000026925	-1.0974	3.21E-07	Inpp5e
ENSMUSG00000025218	-1.0972	1.25E-05	Poll
ENSMUSG00000037995	-1.0971	3.94E-07	Igsf9
ENSMUSG00000038740	-1.0971	1.22E-05	Mvb12b
ENSMUSG00000030727	-1.0968	3.98E-07	Rabep2
ENSMUSG00000015850	-1.0963	0.000285	Adamtsl4
ENSMUSG00000003752	-1.0963	0.000517	Itpkc
ENSMUSG00000021356	-1.0954	3.09E-16	Irf4
ENSMUSG00000003033	-1.0948	6.41E-12	Ap1m1
ENSMUSG00000003813	-1.094	2.21E-08	Rad23a
ENSMUSG00000020926	-1.0928	2.91E-05	Adam11
ENSMUSG00000030424	-1.0928	0.00651	Zfp939
ENSMUSG00000031833	-1.0913	4.78E-08	Mast3
ENSMUSG00000078440	-1.091	0.0116	Dohh
ENSMUSG00000034354	-1.091	1.65E-11	Mtmr3
ENSMUSG00000023942	-1.0898	1.74E-05	Slc29a1
ENSMUSG00000019842	-1.0897	2.13E-05	Traf3ip2
ENSMUSG00000024925	-1.089	0.022631	Rnaseh2c
ENSMUSG00000005417	-1.0883	2.18E-09	Mprip
ENSMUSG00000036966	-1.0869	1.15E-09	Spryd3
ENSMUSG00000020893	-1.0867	6.05E-09	Per1
ENSMUSG00000020329	-1.0861	0.000453	Polrmt
ENSMUSG00000059981	-1.0861	6.09E-12	Taok2
ENSMUSG00000001924	-1.0848	3.19E-16	Uba1
ENSMUSG00000022831	-1.0844	1.04E-14	Hcls1
ENSMUSG00000035671	-1.0843	1.29E-10	Zswim4
ENSMUSG00000039568	-1.0836	1.66E-10	Ubald1

ENSMUSG00000096910	-1.083	8.03E-05	Zfp955b
ENSMUSG00000033767	-1.0822	8.26E-12	Tmem131l
ENSMUSG00000048997	-1.0811	0.000677	Atxn7l2
ENSMUSG00000018974	-1.0807	5.34E-08	Sart3
ENSMUSG00000007950	-1.0799	0.000951	Abhd8
ENSMUSG00000020432	-1.0792	1.84E-06	Tcn2
ENSMUSG00000039953	-1.0781	1.54E-05	Clstn1
ENSMUSG00000028454	-1.0775	0.000171	Pigo
ENSMUSG00000032596	-1.0772	6.46E-10	Uba7
ENSMUSG00000025227	-1.077	8.97E-06	Mfsd13a
ENSMUSG00000030583	-1.0767	2.07E-20	Sipa1l3
ENSMUSG00000039850	-1.0761	2.30E-05	Endov
ENSMUSG00000041654	-1.0758	6.57E-09	Slc39a11
ENSMUSG00000111264	-1.0752	2.21E-08	E030022I16Rik
ENSMUSG00000034771	-1.0747	8.66E-05	Tle2
ENSMUSG00000032392	-1.0746	0.000212	Parp16
ENSMUSG00000034245	-1.0742	0.04101	Hdac11
ENSMUSG00000032513	-1.0733	7.87E-06	Gorasp1
ENSMUSG00000021254	-1.0725	3.42E-08	Gpatch2l
ENSMUSG00000059409	-1.0719	1.80E-08	Ppp2r5d
ENSMUSG00000038807	-1.0719	8.23E-05	Rap1gap2
ENSMUSG00000006800	-1.0719	2.37E-07	Sulf2
ENSMUSG00000024963	-1.0718	4.73E-05	Dnajc4
ENSMUSG00000017837	-1.0713	1.12E-09	Nkiras2
ENSMUSG00000022614	-1.0712	3.14E-08	Lmf2
ENSMUSG00000034570	-1.0705	1.17E-05	Inpp5j
ENSMUSG00000020143	-1.0699	2.06E-22	Dock2
ENSMUSG00000038838	-1.0695	0.001586	Vars2
ENSMUSG00000055681	-1.0681	8.54E-10	Cope
ENSMUSG00000035781	-1.068	1.63E-10	R3hdm4
ENSMUSG00000024169	-1.0674	2.99E-10	Ift140
ENSMUSG00000041939	-1.0664	0.0003	Mvk
ENSMUSG00000017176	-1.0655	0.014886	Nt5c3b
ENSMUSG00000029456	-1.0651	0.004537	Acad10
ENSMUSG00000015605	-1.0648	2.25E-08	Srf
ENSMUSG00000019194	-1.0644	0.00075	Scn1b
ENSMUSG00000017754	-1.064	2.31E-08	Pltp
ENSMUSG00000031787	-1.0635	8.65E-06	Katnb1
ENSMUSG00000037791	-1.0635	3.08E-15	Phf12
ENSMUSG00000017756	-1.0613	2.76E-14	Slc12a7
ENSMUSG00000034194	-1.0611	2.20E-05	R3hcc1
ENSMUSG00000020802	-1.0608	9.68E-08	Ube2o
ENSMUSG00000030035	-1.0604	6.92E-07	Wbp1
ENSMUSG00000023990	-1.06	0.000597	Tfeb
ENSMUSG00000061981	-1.0598	5.44E-11	Flot2
ENSMUSG00000027523	-1.0593	2.55E-05	Gnas
ENSMUSG00000035382	-1.0591	6.26E-09	Pcsk7
ENSMUSG00000022517	-1.0575	2.69E-11	Mgrn1
ENSMUSG00000019978	-1.0573	4.40E-15	Epb41l2
ENSMUSG00000025224	-1.0569	5.09E-13	Gbf1
ENSMUSG00000034595	-1.0557	8.53E-13	Ppp1r18
ENSMUSG00000037405	-1.0549	0.007579	Icam1
ENSMUSG00000029028	-1.0535	7.67E-07	Lrrc47

ENSMUSG00000078716	-1.0534	2.17E-05	Tmem8b
ENSMUSG00000021782	-1.0531	0.001349	Dlg5
ENSMUSG00000022900	-1.0517	0.008544	Ildr1
ENSMUSG00000020538	-1.0517	2.00E-10	Srebf1
ENSMUSG00000034121	-1.0512	0.000282	Mks1
ENSMUSG00000029598	-1.0507	2.69E-09	Plbd2
ENSMUSG00000028041	-1.0489	3.20E-08	Adam15
ENSMUSG00000028463	-1.0487	6.52E-05	Car9
ENSMUSG00000031827	-1.0464	1.27E-13	Cotl1
ENSMUSG00000018661	-1.0461	2.31E-09	Cog1
ENSMUSG00000023495	-1.0461	1.08E-06	Pcbp4
ENSMUSG00000013419	-1.0458	0.00366	Zfp651
ENSMUSG00000020437	-1.0443	3.65E-12	Myo1g
ENSMUSG00000019471	-1.0434	1.05E-12	Cdc37
ENSMUSG00000028064	-1.0433	1.46E-13	Sema4a
ENSMUSG00000066306	-1.0417	1.31E-15	Numa1
ENSMUSG00000024222	-1.0412	3.91E-13	Fkbp5
ENSMUSG00000036270	-1.0404	2.93E-08	Edc4
ENSMUSG00000023034	-1.0404	0.04477	Nr4a1
ENSMUSG00000042350	-1.0403	1.15E-10	Arel1
ENSMUSG00000006611	-1.0382	1.20E-11	Hfe
ENSMUSG00000030595	-1.038	5.48E-09	Nfkbib
ENSMUSG00000001138	-1.0373	8.54E-09	Cnnm3
ENSMUSG00000028459	-1.0367	5.19E-06	Cd72
ENSMUSG00000044037	-1.0362	2.37E-06	Als2cl
ENSMUSG00000031530	-1.0359	7.23E-13	Dusp4
ENSMUSG00000044072	-1.0358	5.38E-06	Eml6
ENSMUSG00000031659	-1.0349	1.52E-13	Adcy7
ENSMUSG00000024858	-1.0348	8.51E-10	Grk2
ENSMUSG00000005410	-1.0342	0.000501	Mcm5
ENSMUSG00000040721	-1.0341	2.63E-07	Zfhx2
ENSMUSG00000002052	-1.0332	3.35E-13	Supt6
ENSMUSG00000024947	-1.0329	9.90E-07	Men1
ENSMUSG00000081684	-1.0321	0.019419	Rps2-ps13
ENSMUSG00000024862	-1.0312	2.79E-06	Klc2
ENSMUSG00000038058	-1.0308	1.07E-07	Nod1
ENSMUSG00000037820	-1.0301	1.51E-12	Tgm2
ENSMUSG00000022779	-1.0297	1.50E-10	Top3b
ENSMUSG00000026856	-1.0273	5.18E-07	Dolpp1
ENSMUSG00000035342	-1.0273	8.14E-06	Lzts2
ENSMUSG00000036427	-1.0265	9.20E-11	Gpi1
ENSMUSG00000013089	-1.0264	3.52E-06	Etv5
ENSMUSG00000029250	-1.0261	1.33E-09	Polr2b
ENSMUSG00000021257	-1.0255	0.003185	Angel1
ENSMUSG00000010047	-1.0254	0.000973	Hyal2
ENSMUSG00000036026	-1.0254	0.013652	Tmem63b
ENSMUSG00000057948	-1.0254	3.88E-10	Unc13d
ENSMUSG00000001128	-1.0252	3.15E-09	Cfp
ENSMUSG00000074886	-1.0252	1.02E-07	Grk6
ENSMUSG00000028973	-1.0248	0.00051	Abcb8
ENSMUSG00000052085	-1.0241	3.61E-14	Dock8
ENSMUSG00000029860	-1.0238	1.17E-07	Zyx
ENSMUSG00000032409	-1.023	5.00E-14	Atr

ENSMUSG00000063524	-1.023	2.66E-10	Eno1
ENSMUSG00000035273	-1.0229	5.17E-12	Hpse
ENSMUSG00000018925	-1.0222	2.93E-07	Heatr9
ENSMUSG00000035673	-1.02	2.30E-07	Sbno2
ENSMUSG00000042215	-1.0199	0.002303	Bag2
ENSMUSG00000027198	-1.0193	1.45E-07	Ext2
ENSMUSG00000047921	-1.0192	9.33E-08	Trappc9
ENSMUSG00000055200	-1.0187	0.000172	Sertad3
ENSMUSG00000070305	-1.0181	1.01E-06	Mpzl3
ENSMUSG00000025574	-1.018	1.65E-09	Tk1
ENSMUSG00000027263	-1.0163	1.03E-10	Tubgcp4
ENSMUSG00000004113	-1.0153	1.10E-06	Cacna1b
ENSMUSG00000024525	-1.0149	1.71E-06	Impa2
ENSMUSG00000045216	-1.0148	2.85E-05	Hs6st1
ENSMUSG00000043068	-1.0147	3.12E-05	Fam89a
ENSMUSG00000062157	-1.014	0.013465	Ifnlr1
ENSMUSG00000039183	-1.0137	5.97E-08	Nubp2
ENSMUSG00000001525	-1.0129	1.07E-15	Tubb5
ENSMUSG00000041697	-1.0124	1.67E-09	Cox6a1
ENSMUSG00000024941	-1.0124	9.15E-09	Scyl1
ENSMUSG00000026070	-1.0122	0.013607	Il18r1
ENSMUSG00000059851	-1.0109	4.12E-10	Kmt5c
ENSMUSG00000019489	-1.01	0.005374	Cd70
ENSMUSG00000020876	-1.0089	3.39E-09	Snx11
ENSMUSG00000034201	-1.0087	3.31E-09	Gas2l1
ENSMUSG00000039901	-1.0077	8.77E-06	Armh3
ENSMUSG00000024831	-1.0073	0.000145	Ighmbp2
ENSMUSG00000033161	-1.0071	1.02E-12	Atp1a1
ENSMUSG00000026797	-1.007	1.67E-10	Stxbp1
ENSMUSG00000044783	-1.0063	4.30E-15	Hjurp
ENSMUSG00000039849	-1.0062	3.19E-06	Pcif1
ENSMUSG00000020260	-1.006	2.17E-06	Pofut2
ENSMUSG00000007036	-1.0056	1.75E-07	Abhd16a
ENSMUSG00000033712	-1.0054	1.89E-12	Ccar2
ENSMUSG00000020190	-1.005	2.03E-11	Mknk2
ENSMUSG00000020235	-1.0049	9.45E-08	Fzr1
ENSMUSG00000030528	-1.0048	8.28E-05	Blm
ENSMUSG00000056071	-1.0039	0.009726	S100a9
ENSMUSG00000026269	-1.0035	1.57E-09	Rnpepl1
ENSMUSG00000034994	-1.0032	3.61E-10	Eef2
ENSMUSG00000028433	-1.003	6.94E-09	Ubap2
ENSMUSG00000073423	-1.0025	0.001616	Zfp414
ENSMUSG00000025422	-1.0023	0.000236	Agap2
ENSMUSG00000035203	-1.0017	3.47E-07	Epn1
ENSMUSG00000042303	-1.0006	0.000151	Sgsm3
ENSMUSG00000022440	-1.0004	5.98E-06	C1qtnf6
ENSMUSG00000095098	-1.0001	0.025775	Ccdc85b
ENSMUSG00000024359	1.0004	5.11E-15	Hspa9
ENSMUSG00000066000	1.002	0.034539	Zfp979
ENSMUSG00000016833	1.0024	7.86E-07	Mrps18c
ENSMUSG00000035329	1.0029	5.02E-10	Fbxo33
ENSMUSG00000069862	1.0039	0.044263	Rps12-ps9
ENSMUSG00000042541	1.0052	3.56E-12	Sem1

ENSMUSG00000024583	1.007	7.71E-12	Txnl1
ENSMUSG00000020863	1.0071	5.96E-12	Luc7l3
ENSMUSG00000022106	1.0073	6.03E-09	Rcbtb2
ENSMUSG00000020074	1.0076	8.07E-10	Ccar1
ENSMUSG00000027739	1.0088	1.70E-09	Rab33b
ENSMUSG00000037443	1.0096	1.21E-14	Cep85
ENSMUSG00000032902	1.0107	3.15E-05	Slc16a1
ENSMUSG00000073616	1.0108	2.63E-11	Cops9
ENSMUSG00000032320	1.0111	6.87E-07	Rcn2
ENSMUSG00000018425	1.0119	8.93E-16	Dhx40
ENSMUSG00000021466	1.0121	2.01E-08	Ptch1
ENSMUSG00000024712	1.0122	2.70E-15	Rfk
ENSMUSG00000066456	1.0131	1.53E-06	Hmgn3
ENSMUSG00000057858	1.0141	3.20E-05	Fam204a
ENSMUSG00000078349	1.0152	1.08E-05	AW011738
ENSMUSG00000036309	1.0159	2.44E-12	Skp1a
ENSMUSG00000022698	1.016	1.19E-10	Naa50
ENSMUSG00000047881	1.0169	1.46E-06	Rell1
ENSMUSG00000063172	1.0177	3.37E-07	Hspb11
ENSMUSG00000021519	1.0187	6.30E-11	Mterf3
ENSMUSG00000021025	1.0197	6.07E-13	Nfkbia
ENSMUSG00000010751	1.02	0.000409	Tnfrsf22
ENSMUSG00000047368	1.0203	6.73E-07	Abhd17b
ENSMUSG00000027822	1.0209	7.53E-13	Slc33a1
ENSMUSG00000115946	1.021	0.018005	Mirt2
ENSMUSG00000028936	1.022	2.93E-15	Rpl22
ENSMUSG00000048924	1.0222	0.011129	Ccdc125
ENSMUSG00000033386	1.0234	3.03E-09	Frrs1
ENSMUSG00000040152	1.0284	1.69E-12	Thbs1
ENSMUSG00000021982	1.0287	6.94E-09	Cdadc1
ENSMUSG00000029390	1.0303	5.28E-20	Tmed2
ENSMUSG00000053012	1.0304	4.11E-11	Krcc1
ENSMUSG00000028575	1.0325	0.035927	Eqtn
ENSMUSG00000052033	1.033	9.62E-09	Pfdn4
ENSMUSG00000024084	1.0331	8.92E-07	Qpct
ENSMUSG00000071072	1.0332	9.33E-14	Ptges3
ENSMUSG00000022020	1.0334	1.44E-07	Naa16
ENSMUSG00000052688	1.0336	1.17E-05	Rab7b
ENSMUSG00000038010	1.0338	0.003042	Ccdc138
ENSMUSG00000115230	1.0348	4.29E-05	AU022793
ENSMUSG00000036977	1.0357	6.85E-09	Anapc10
ENSMUSG00000034205	1.036	4.73E-05	Loxl2
ENSMUSG00000079641	1.0373	2.04E-15	Rpl39
ENSMUSG00000060002	1.0375	1.64E-09	Chpt1
ENSMUSG00000024270	1.0378	2.58E-09	Slc39a6
ENSMUSG00000027357	1.038	7.58E-08	Crls1
ENSMUSG00000022323	1.0387	2.92E-06	Rida
ENSMUSG00000028161	1.0392	1.34E-13	Ppp3ca
ENSMUSG00000044408	1.04	1.25E-13	Sptssa
ENSMUSG00000027236	1.0402	6.92E-08	Eif3j1
ENSMUSG00000039899	1.0407	4.58E-10	Fgl2
ENSMUSG00000050668	1.0412	8.82E-08	Gpatch11
ENSMUSG00000039232	1.0431	4.46E-19	Stx11

ENSMUSG00000008859	1.0436	8.95E-12	Rala
ENSMUSG00000038374	1.0445	4.97E-16	Rbm8a
ENSMUSG00000075266	1.0449	0.041806	Cenpw
ENSMUSG00000036572	1.0449	2.02E-08	Upf3b
ENSMUSG00000039234	1.045	4.85E-10	Sec24d
ENSMUSG00000006818	1.0454	1.04E-17	Sod2
ENSMUSG00000087141	1.0465	0.000938	Plcxd2
ENSMUSG00000014075	1.0465	3.38E-09	Tctex1d2
ENSMUSG00000078713	1.0486	1.79E-10	Tomm5
ENSMUSG00000034109	1.049	1.78E-08	Golim4
ENSMUSG00000078941	1.05	6.88E-07	Ak6
ENSMUSG00000063245	1.0507	7.40E-05	Zfp993
ENSMUSG00000028327	1.0513	0.006946	Stra6l
ENSMUSG00000072620	1.0516	4.48E-21	Slfn2
ENSMUSG00000020664	1.0546	3.17E-09	Dld
ENSMUSG00000024646	1.0572	7.15E-23	Cyb5a
ENSMUSG00000025804	1.0579	8.81E-12	Ccr1
ENSMUSG00000022419	1.0585	0.00768	Deptor
ENSMUSG00000072704	1.0586	3.36E-08	Smim10l1
ENSMUSG00000025351	1.0587	1.11E-16	Cd63
ENSMUSG00000045573	1.0596	3.65E-15	Penk
ENSMUSG00000024597	1.0602	3.01E-05	Slc12a2
ENSMUSG00000026872	1.0618	4.94E-10	Zeb2
ENSMUSG00000026600	1.0628	5.37E-14	Soat1
ENSMUSG00000033917	1.0629	1.32E-14	Gde1
ENSMUSG00000000581	1.0635	2.93E-07	C1d
ENSMUSG00000071415	1.0641	1.21E-21	Rpl23
ENSMUSG00000079555	1.0648	2.77E-07	Haus3
ENSMUSG00000025979	1.0665	2.16E-11	Mob4
ENSMUSG00000026097	1.0665	2.74E-08	Ormdl1
ENSMUSG00000028035	1.0674	1.27E-13	Dnajb4
ENSMUSG00000041777	1.0675	1.85E-10	Cir1
ENSMUSG00000026083	1.071	2.79E-11	Eif5b
ENSMUSG00000046093	1.0711	0.003209	Hpcal4
ENSMUSG00000018927	1.0714	1.89E-15	Ccl6
ENSMUSG00000028609	1.0717	2.58E-09	Magoh
ENSMUSG00000039958	1.0721	1.54E-09	Etfbkmt
ENSMUSG00000004897	1.0725	2.46E-12	Hdgf
ENSMUSG00000042734	1.0727	1.32E-09	Ttc9
ENSMUSG00000062082	1.0734	2.30E-08	Cd200r4
ENSMUSG00000086429	1.0736	1.98E-07	Gt(ROSA)26Sor
ENSMUSG00000083822	1.0741	0.009851	Hmgb1-ps5
ENSMUSG00000027074	1.0748	4.30E-10	Slc43a3
ENSMUSG00000029186	1.0781	1.96E-14	Pi4k2b
ENSMUSG00000019777	1.0786	1.50E-09	Hdac2
ENSMUSG00000032251	1.0797	0.035072	Irak1bp1
ENSMUSG00000021250	1.0808	2.17E-08	Fos
ENSMUSG00000098274	1.0814	0.00354	Rpl24
ENSMUSG00000019857	1.0841	3.73E-06	Asf1a
ENSMUSG00000082284	1.0853	8.54E-05	H3f3a-ps1
ENSMUSG00000025746	1.0855	2.22E-11	Il6
ENSMUSG00000025508	1.0855	9.39E-18	Rplp2
ENSMUSG00000022863	1.0858	3.14E-06	Btg3

ENSMUSG00000048895	1.0858	1.60E-07	Cdk5r1
ENSMUSG00000026456	1.0862	6.33E-08	Cyb5r1
ENSMUSG00000055639	1.0875	4.03E-08	Dach1
ENSMUSG00000032715	1.0879	3.34E-07	Trib3
ENSMUSG00000030742	1.0887	0.03257	Lat
ENSMUSG00000025451	1.0888	1.27E-11	Paip1
ENSMUSG00000027706	1.0892	7.19E-13	Sec62
ENSMUSG00000031352	1.0895	6.20E-08	Hccs
ENSMUSG00000045672	1.0897	5.08E-12	Col27a1
ENSMUSG00000113948	1.0899	0.007104	Rpl17-ps3
ENSMUSG00000040855	1.0913	0.008256	Reps2
ENSMUSG00000048007	1.0926	0.00181	Timm8a1
ENSMUSG00000097754	1.0928	0.001209	Ptgs2os2
ENSMUSG00000032575	1.0937	9.46E-19	Manf
ENSMUSG00000038402	1.0939	0.010452	Foxf2
ENSMUSG00000079143	1.0954	0.003994	Atxn7l1os1
ENSMUSG00000044768	1.0961	6.34E-09	D1Ertd622e
ENSMUSG00000063415	1.0966	5.63E-11	Cyp26b1
ENSMUSG00000020328	1.0973	1.09E-10	Nudcd2
ENSMUSG00000023074	1.0977	1.99E-07	Mospd1
ENSMUSG00000010721	1.0992	1.52E-08	Lmbr1
ENSMUSG00000063882	1.1	4.19E-19	Uqcrh
ENSMUSG00000038622	1.1012	1.64E-08	Med30
ENSMUSG00000075701	1.1026	6.57E-16	Selenos
ENSMUSG00000026482	1.1037	1.57E-11	Rgl1
ENSMUSG00000027968	1.106	4.60E-07	Larp7
ENSMUSG00000031445	1.1078	0.008561	Proz
ENSMUSG00000079652	1.1087	0.001812	Fam71f2
ENSMUSG00000055480	1.1092	0.012957	Zfp458
ENSMUSG00000037406	1.1096	0.003397	Htra4
ENSMUSG00000005883	1.1096	0.026351	Spo11
ENSMUSG00000000982	1.1098	2.84E-14	Ccl3
ENSMUSG00000031362	1.11	0.022586	Xlr4c
ENSMUSG00000051397	1.1129	0.004236	Tacstd2
ENSMUSG00000037849	1.1139	1.33E-11	Ifi206
ENSMUSG00000052415	1.1145	4.45E-09	Tchh
ENSMUSG00000051579	1.1155	3.43E-09	Tceal8
ENSMUSG00000022838	1.1158	0.024449	Eaf2
ENSMUSG00000028698	1.1167	0.007475	Pik3r3
ENSMUSG00000031762	1.121	0.003364	Mt2
ENSMUSG00000046688	1.122	4.85E-07	Tifa
ENSMUSG00000031590	1.123	1.63E-11	Frg1
ENSMUSG00000093661	1.1231	4.90E-12	Eif4e3
ENSMUSG00000015759	1.1235	4.98E-13	Cnih1
ENSMUSG00000028221	1.1235	1.89E-15	Pip4p2
ENSMUSG00000014496	1.1237	1.10E-09	Ankrd28
ENSMUSG00000021930	1.1238	1.22E-08	Spryd7
ENSMUSG00000066595	1.1242	2.76E-12	Flvcr1
ENSMUSG00000087687	1.1252	9.88E-08	Pet100
ENSMUSG00000042043	1.1258	3.59E-12	Tbca
ENSMUSG00000019122	1.1263	1.20E-17	Ccl9
ENSMUSG00000031226	1.1287	3.12E-10	Pbdc1
ENSMUSG00000113902	1.1289	2.07E-10	Ndufb1-ps

ENSMUSG00000034586	1.1323	5.50E-07	Hid1
ENSMUSG00000025764	1.1333	1.78E-10	Jade1
ENSMUSG00000016534	1.134	1.04E-13	Lamp2
ENSMUSG00000005686	1.1348	1.22E-13	Ampd3
ENSMUSG00000025967	1.1366	1.78E-21	Eef1b2
ENSMUSG00000028295	1.1368	2.88E-07	Smim8
ENSMUSG00000047648	1.137	9.99E-12	Fbxo30
ENSMUSG00000028367	1.1381	1.09E-24	Txn1
ENSMUSG00000026107	1.1405	1.32E-14	Nabp1
ENSMUSG00000041075	1.1414	1.54E-09	Fzd7
ENSMUSG00000058135	1.1414	6.66E-19	Gstm1
ENSMUSG00000028261	1.1419	6.55E-07	Ndufaf4
ENSMUSG00000063714	1.1421	0.000231	Sp3os
ENSMUSG00000069792	1.1422	5.72E-12	Wfdc17
ENSMUSG00000032024	1.143	8.66E-11	Clmp
ENSMUSG00000000730	1.1431	3.96E-06	Dnmt3l
ENSMUSG00000018930	1.144	4.61E-11	Ccl4
ENSMUSG00000098234	1.1446	6.26E-11	Snhg6
ENSMUSG00000051989	1.1447	3.62E-06	Smim11
ENSMUSG00000105388	1.1449	1.25E-06	Rpl36a-ps2
ENSMUSG00000062248	1.1453	2.83E-08	Cks2
ENSMUSG00000055435	1.1471	3.36E-06	Maf
ENSMUSG00000095687	1.1475	0.047657	Rnaset2a
ENSMUSG00000045502	1.1481	1.64E-10	Hcar2
ENSMUSG00000020137	1.1481	1.00E-07	Thap2
ENSMUSG00000068882	1.149	1.98E-11	Ssb
ENSMUSG00000030268	1.1494	6.09E-06	Bcat1
ENSMUSG00000036368	1.1524	0.000629	Rmdn2
ENSMUSG00000028291	1.1543	1.60E-10	Akirin2
ENSMUSG00000058755	1.1543	1.34E-19	Osm
ENSMUSG00000044667	1.1556	0.007969	Plppr4
ENSMUSG00000085793	1.1562	1.31E-05	Lin52
ENSMUSG00000064363	1.1563	3.84E-12	mt-Nd4
ENSMUSG00000027132	1.1573	3.61E-11	Katnbl1
ENSMUSG00000030659	1.1574	1.68E-09	Nucb2
ENSMUSG00000029486	1.1596	1.26E-09	Mrpl1
ENSMUSG00000033355	1.1597	3.00E-19	Rtp4
ENSMUSG00000021758	1.1598	0.013534	Ddx4
ENSMUSG00000023467	1.1609	0.000425	Tulp2
ENSMUSG00000023349	1.1611	1.61E-27	Clec4n
ENSMUSG00000079184	1.1617	6.14E-09	Mphosph8
ENSMUSG00000031007	1.1653	1.75E-14	Atp6ap2
ENSMUSG00000043881	1.1657	1.92E-06	Kbtbd7
ENSMUSG00000102824	1.1662	1.40E-05	Pdcd5-ps
ENSMUSG00000027676	1.167	1.13E-06	Ccdc39
ENSMUSG00000058351	1.167	0.000124	Smim4
ENSMUSG00000028232	1.1677	3.55E-11	Tmem68
ENSMUSG00000001700	1.1682	1.92E-05	Gramd3
ENSMUSG00000026970	1.169	4.22E-14	Rbms1
ENSMUSG00000044583	1.1692	1.48E-06	Tlr7
ENSMUSG00000064372	1.1728	3.28E-14	mt-Tp
ENSMUSG00000022205	1.1742	5.11E-19	Sub1
ENSMUSG00000025779	1.1744	1.88E-10	Ly96

ENSMUSG00000043421	1.1753	2.65E-16	Hilpda
ENSMUSG00000030878	1.1754	0.000184	Cdr2
ENSMUSG00000085178	1.1804	0.012657	Kdm6bos
ENSMUSG00000036214	1.1821	0.049263	Znrd1as
ENSMUSG00000047749	1.1838	4.15E-05	Zc3hav1l
ENSMUSG00000029086	1.1854	0.000646	Prom1
ENSMUSG00000063779	1.1868	3.54E-05	Chil4
ENSMUSG00000071052	1.1877	6.10E-07	Rpl7a-ps5
ENSMUSG00000023966	1.1878	0.021892	Rsph9
ENSMUSG00000038692	1.1885	4.49E-08	Hoxb4
ENSMUSG00000031266	1.1896	5.35E-20	Gla
ENSMUSG00000050148	1.19	2.33E-10	Ubqln2
ENSMUSG00000001774	1.1901	4.79E-12	Chordc1
ENSMUSG00000000605	1.1906	5.88E-14	Clcn4
ENSMUSG00000040370	1.1921	2.47E-11	Etfrf1
ENSMUSG00000067288	1.1926	3.74E-19	Rps28
ENSMUSG00000026315	1.1953	1.82E-05	Serpinb8
ENSMUSG00000021552	1.196	1.44E-10	Gkap1
ENSMUSG00000039001	1.1973	3.85E-25	Rps21
ENSMUSG00000051950	1.1982	2.27E-09	B3glct
ENSMUSG00000060636	1.2011	7.82E-19	Rpl35a
ENSMUSG00000047898	1.2012	7.63E-07	Ccr4
ENSMUSG00000047965	1.2034	1.49E-07	Rpl9-ps7
ENSMUSG00000023961	1.2048	2.32E-05	Enpp4
ENSMUSG00000043415	1.2066	4.04E-10	Otud1
ENSMUSG00000071856	1.2071	0.038458	Mcc
ENSMUSG00000031639	1.2076	9.12E-05	Tlr3
ENSMUSG00000040429	1.2094	4.76E-05	Mterf1a
ENSMUSG00000052270	1.2109	2.77E-11	Fpr2
ENSMUSG00000060126	1.2165	5.35E-21	Tpt1
ENSMUSG00000026511	1.2175	1.74E-13	Srp9
ENSMUSG00000025980	1.2178	4.80E-18	Hspd1
ENSMUSG00000020099	1.2188	0.041444	Unc5b
ENSMUSG00000090641	1.2188	0.006995	Zfp712
ENSMUSG00000042133	1.2195	2.77E-12	Ppig
ENSMUSG00000022707	1.2257	8.82E-16	Gbe1
ENSMUSG00000028007	1.2262	7.72E-14	Snx7
ENSMUSG00000072623	1.2293	0.009403	Zfp9
ENSMUSG00000071226	1.2317	0.031377	Cecr2
ENSMUSG00000029553	1.2318	1.19E-15	Tfec
ENSMUSG00000036478	1.2323	3.58E-22	Btg1
ENSMUSG00000046675	1.2343	5.52E-12	Tmem251
ENSMUSG00000025757	1.2345	4.14E-18	Hspa4l
ENSMUSG00000021520	1.2389	9.90E-13	Uqcrb
ENSMUSG00000023068	1.2397	4.01E-17	Nus1
ENSMUSG00000028347	1.2398	5.47E-08	Tmeff1
ENSMUSG00000074579	1.2432	0.000927	Lekr1
ENSMUSG00000031584	1.2448	1.30E-22	Gsr
ENSMUSG00000028180	1.245	3.81E-14	Zranb2
ENSMUSG00000044033	1.2451	1.04E-05	Ccdc141
ENSMUSG00000035530	1.251	1.46E-21	Eif1
ENSMUSG00000078771	1.2511	5.44E-14	Evi2a
ENSMUSG00000019960	1.2541	5.26E-07	Dusp6

ENSMUSG00000040713	1.2587	1.66E-17	Creg1
ENSMUSG00000014905	1.2603	7.70E-16	Dnajb9
ENSMUSG00000067297	1.2621	0.025954	Ifit1bl2
ENSMUSG00000077737	1.2628	0.011897	Snord72
ENSMUSG00000019817	1.2629	0.028982	Plagl1
ENSMUSG00000046982	1.2634	6.68E-11	Tshz1
ENSMUSG00000002475	1.2686	0.005144	Abhd3
ENSMUSG00000047843	1.2717	2.56E-18	Bri3
ENSMUSG00000062960	1.2721	6.24E-09	Kdr
ENSMUSG00000060429	1.2725	0.000269	Sntb1
ENSMUSG00000082044	1.2727	4.65E-06	Snrpert
ENSMUSG00000024679	1.2738	7.62E-18	Ms4a6d
ENSMUSG00000031609	1.2749	5.31E-14	Sap30
ENSMUSG00000099583	1.2752	0.04154	Hist1h3d
ENSMUSG00000028249	1.2775	1.93E-15	Sdcbp
ENSMUSG00000027276	1.2797	4.23E-14	Jag1
ENSMUSG00000040133	1.2805	0.036903	Gpr176
ENSMUSG00000030142	1.2834	4.89E-14	Clec4e
ENSMUSG00000028517	1.2866	8.49E-16	Plpp3
ENSMUSG00000069911	1.2874	0.040241	Fam196b
ENSMUSG00000037266	1.2879	8.91E-25	Rsrp1
ENSMUSG00000021770	1.2894	1.35E-16	Samd8
ENSMUSG00000074754	1.2908	6.33E-05	Smim26
ENSMUSG00000045624	1.2958	1.40E-10	Esf1
ENSMUSG00000058600	1.2982	1.70E-17	Rpl30
ENSMUSG00000046573	1.2983	4.20E-09	Lyrm4
ENSMUSG00000056300	1.3018	0.01332	Zfp981
ENSMUSG00000049130	1.3028	0.003195	C5ar1
ENSMUSG00000039196	1.3045	2.87E-12	Orm1
ENSMUSG00000036863	1.3046	0.001016	Syde2
ENSMUSG00000021716	1.3063	2.56E-11	Srek1ip1
ENSMUSG00000024696	1.3082	1.95E-10	Lpxn
ENSMUSG00000028497	1.3105	8.25E-08	Hacd4
ENSMUSG00000034647	1.3105	4.59E-14	Ankrd12
ENSMUSG00000054000	1.3116	0.001715	Tusc1
ENSMUSG00000005054	1.3119	2.26E-25	Cstb
ENSMUSG00000031245	1.3127	6.03E-06	Hmgn5
ENSMUSG00000025037	1.3143	2.18E-10	Maoa
ENSMUSG00000027834	1.3148	0.007355	Serpini1
ENSMUSG00000024072	1.3151	1.24E-12	Yipf4
ENSMUSG00000074896	1.3166	2.75E-19	Ifit3
ENSMUSG00000044103	1.3171	2.17E-14	Il1f9
ENSMUSG00000045996	1.318	1.44E-08	Polr2k
ENSMUSG00000027274	1.3184	0.000598	Mkks
ENSMUSG00000021290	1.3185	4.20E-15	Atp5mpl
ENSMUSG00000051022	1.3201	0.031004	Hs3st1
ENSMUSG00000022122	1.322	2.54E-15	Ednrb
ENSMUSG00000026701	1.3273	3.50E-22	Prdx6
ENSMUSG00000021775	1.3363	7.79E-16	Nr1d2
ENSMUSG00000073676	1.3365	3.69E-20	Hspe1
ENSMUSG00000057315	1.3386	0.012076	Arhgap24
ENSMUSG00000027808	1.3416	2.29E-23	Serp1
ENSMUSG00000090553	1.3417	8.71E-14	Snrpe

ENSMUSG00000023868	1.3434	0.001978	Pde10a
ENSMUSG00000036902	1.3461	0.02482	Neto2
ENSMUSG00000084319	1.347	2.79E-20	Tpt1-ps3
ENSMUSG00000023915	1.3494	3.50E-22	Tnfrsf21
ENSMUSG00000059325	1.3511	7.01E-16	Hopx
ENSMUSG00000079435	1.3541	3.37E-21	Rpl36a
ENSMUSG00000027611	1.3566	1.27E-26	Procr
ENSMUSG00000021054	1.3574	7.03E-13	Sgpp1
ENSMUSG00000054619	1.3577	4.05E-20	Mettl7a1
ENSMUSG00000062328	1.3594	0.042373	Rpl17
ENSMUSG00000052783	1.367	6.68E-06	Grk4
ENSMUSG00000027698	1.3684	1.85E-13	Nceh1
ENSMUSG00000015947	1.3716	1.30E-11	Fcgr1
ENSMUSG00000005800	1.3724	4.68E-11	Mmp8
ENSMUSG00000068587	1.3727	0.021614	Mgam
ENSMUSG00000025938	1.3773	3.93E-05	Slco5a1
ENSMUSG00000029762	1.3819	7.96E-08	Akr1b8
ENSMUSG00000064341	1.3827	4.40E-05	mt-Nd1
ENSMUSG00000052565	1.3832	0.036509	Hist1h1d
ENSMUSG00000033022	1.3907	0.009864	Cdo1
ENSMUSG00000085148	1.3915	1.98E-24	Mir22hg
ENSMUSG00000024430	1.3922	3.90E-05	Cabyr
ENSMUSG00000037287	1.3982	1.73E-21	Tbcel
ENSMUSG00000045362	1.3991	2.02E-19	Tnfrsf26
ENSMUSG00000034557	1.4009	0.002338	Zfyve9
ENSMUSG00000046997	1.4039	1.54E-05	Spsb4
ENSMUSG00000006732	1.404	0.000112	Mettl1
ENSMUSG00000021996	1.4067	8.68E-33	Esd
ENSMUSG00000057123	1.4069	9.58E-18	Gja5
ENSMUSG00000030157	1.4189	1.89E-19	Clec2d
ENSMUSG00000004359	1.4201	7.43E-08	Spic
ENSMUSG00000030256	1.4205	1.47E-11	Bhlhe41
ENSMUSG00000034898	1.4229	0.013304	Filip1
ENSMUSG00000061175	1.4267	4.36E-23	Fnip2
ENSMUSG00000041488	1.431	1.51E-16	Stx3
ENSMUSG00000032418	1.4323	3.79E-11	Me1
ENSMUSG00000034226	1.4354	2.40E-07	Rhov
ENSMUSG00000034460	1.4365	0.003765	Six4
ENSMUSG00000025355	1.4384	3.51E-19	Mmp19
ENSMUSG00000024087	1.441	1.45E-21	Cyp1b1
ENSMUSG00000097028	1.4443	0.003565	Ptgs2os
ENSMUSG00000025290	1.4483	9.50E-22	Rps24
ENSMUSG00000038570	1.4489	0.043546	Saxo2
ENSMUSG00000027313	1.4502	0.002711	Chac1
ENSMUSG00000040747	1.4511	1.19E-23	Cd53
ENSMUSG00000025431	1.4524	6.54E-05	Crisp1
ENSMUSG00000024677	1.4536	5.68E-08	Ms4a6b
ENSMUSG00000047757	1.4613	0.045672	Fancb
ENSMUSG00000023927	1.4617	1.05E-14	Satb1
ENSMUSG00000000392	1.4624	7.02E-06	Fap
ENSMUSG00000030219	1.4627	0.025746	Erp27
ENSMUSG00000079101	1.4631	0.003889	Esd-ps
ENSMUSG00000061666	1.465	4.26E-08	Gdpd1

ENSMUSG00000032487	1.4662	4.95E-15	Ptgs2
ENSMUSG00000040405	1.4668	0.00459	Havcr1
ENSMUSG00000062310	1.4708	4.76E-06	Glrp1
ENSMUSG00000090516	1.4723	0.000449	Rps11-ps1
ENSMUSG00000025591	1.4749	1.26E-36	Tma16
ENSMUSG00000040717	1.4806	0.004921	Il17rd
ENSMUSG00000031202	1.4812	0.014816	Rab39b
ENSMUSG00000042212	1.4831	0.005691	Sprr2d
ENSMUSG00000026317	1.4842	1.64E-21	Cln8
ENSMUSG00000109864	1.486	3.79E-10	Eid3
ENSMUSG00000042712	1.4904	2.07E-10	Tceal9
ENSMUSG00000046101	1.4907	0.022219	Mcmdc2
ENSMUSG00000037434	1.5003	4.99E-12	Slc30a1
ENSMUSG00000079480	1.5006	2.12E-13	Pin4
ENSMUSG00000019838	1.5018	1.37E-05	Slc16a10
ENSMUSG00000029304	1.5042	2.13E-17	Spp1
ENSMUSG00000074656	1.5067	7.04E-15	Eif2s2
ENSMUSG00000028266	1.5093	5.45E-16	Lmo4
ENSMUSG00000004105	1.5126	2.66E-10	Angptl2
ENSMUSG00000068205	1.5142	0.005925	Macrod2
ENSMUSG00000039770	1.5144	3.65E-28	Ypel5
ENSMUSG00000021125	1.5162	4.62E-10	Arg2
ENSMUSG00000043668	1.518	8.78E-14	Tox3
ENSMUSG00000049804	1.5304	5.96E-12	Armcx4
ENSMUSG00000028407	1.531	0.024896	Smim27
ENSMUSG00000021270	1.5323	2.33E-22	Hsp90aa1
ENSMUSG00000033446	1.5421	8.03E-16	Lpar6
ENSMUSG00000045690	1.5459	9.74E-24	Wdr89
ENSMUSG00000052889	1.5495	8.28E-12	Prkcb
ENSMUSG00000019970	1.5495	8.99E-21	Sgk1
ENSMUSG00000090110	1.5556	3.77E-12	Cmc4
ENSMUSG00000064345	1.5577	4.83E-05	mt-Nd2
ENSMUSG00000048988	1.5643	0.030325	Elfn1
ENSMUSG00000068263	1.5644	0.002538	Efcc1
ENSMUSG00000063406	1.5656	5.13E-16	Tmed5
ENSMUSG00000055301	1.5669	0.024774	Adh7
ENSMUSG00000026712	1.5677	1.98E-06	Mrc1
ENSMUSG00000053178	1.5687	0.000193	Mterf1b
ENSMUSG00000034317	1.5733	3.97E-14	Trim59
ENSMUSG00000018217	1.5748	1.59E-18	Pmp22
ENSMUSG00000021477	1.5783	1.79E-27	Ctsl
ENSMUSG00000032561	1.5791	3.55E-29	Acpp
ENSMUSG00000041453	1.5814	0.005376	Rpl21
ENSMUSG00000036009	1.5821	0.001419	Mettl25
ENSMUSG00000039221	1.5849	5.47E-18	Rpl22l1
ENSMUSG00000026986	1.5853	0.01877	Hnmt
ENSMUSG00000022816	1.5865	4.96E-12	Fstl1
ENSMUSG00000103653	1.587	0.000729	Gstp-ps
ENSMUSG00000063275	1.591	0.003795	Hacd1
ENSMUSG00000061039	1.5911	1.05E-27	Olfr920
ENSMUSG00000041857	1.598	0.008261	Oosp1
ENSMUSG00000035954	1.6001	2.25E-12	Dock4
ENSMUSG00000029379	1.601	2.08E-24	Cxcl3

ENSMUSG00000028124	1.6072	2.28E-37	Gclm
ENSMUSG00000003279	1.6075	0.004673	Dlgap1
ENSMUSG00000032113	1.6083	2.64E-05	Chek1
ENSMUSG00000028378	1.6165	1.47E-33	Ptgr1
ENSMUSG00000047632	1.6187	1.57E-08	Fgfbp3
ENSMUSG00000020638	1.6189	1.66E-23	Cmpk2
ENSMUSG00000037887	1.6203	0.021477	Dusp8
ENSMUSG00000053580	1.6217	7.42E-16	Tanc2
ENSMUSG00000054932	1.6218	4.73E-05	Afp
ENSMUSG00000057322	1.6218	1.93E-26	Rpl38
ENSMUSG00000023089	1.6229	9.23E-13	Ndufa5
ENSMUSG00000036390	1.6253	6.78E-06	Gadd45a
ENSMUSG00000052534	1.6261	9.67E-11	Pbx1
ENSMUSG00000109509	1.629	0.000281	Rps12-ps4
ENSMUSG00000107369	1.63	0.000843	Gstm2-ps1
ENSMUSG00000083411	1.6309	2.37E-31	Rpl30-ps10
ENSMUSG00000064360	1.6361	0.002296	mt-Nd3
ENSMUSG00000081087	1.6408	2.68E-06	Rps15a-ps7
ENSMUSG00000028494	1.6447	1.89E-32	Plin2
ENSMUSG00000089726	1.6448	5.12E-16	Mir17hg
ENSMUSG00000042035	1.6458	5.27E-05	Igsf3
ENSMUSG00000038539	1.6472	5.20E-40	Atf5
ENSMUSG00000048153	1.6522	0.044754	Olfr49
ENSMUSG00000029761	1.6556	0.002249	Cald1
ENSMUSG00000062345	1.6618	2.47E-05	Serpinb2
ENSMUSG00000034459	1.6652	2.79E-24	Ifit1
ENSMUSG00000038248	1.6679	5.96E-05	Sobp
ENSMUSG00000064359	1.6684	0.0119	mt-Tg
ENSMUSG00000025586	1.6689	1.11E-10	Cpeb1
ENSMUSG00000040167	1.6729	9.52E-06	Ikzf5
ENSMUSG00000057321	1.6729	0.018791	Usp17ld
ENSMUSG00000014226	1.674	3.18E-28	Cacybp
ENSMUSG00000064346	1.674	0.003787	mt-Tw
ENSMUSG00000027737	1.6747	6.38E-27	Slc7a11
ENSMUSG00000087107	1.682	1.09E-24	AI662270
ENSMUSG00000095892	1.6887	0.041791	Rnu5g
ENSMUSG00000023913	1.6914	6.35E-38	Pla2g7
ENSMUSG00000022015	1.6953	2.47E-09	Tnfsf11
ENSMUSG00000039982	1.7028	3.35E-29	Dtx4
ENSMUSG00000001627	1.7161	1.78E-19	Ifrd1
ENSMUSG00000039960	1.7237	2.45E-14	Rhou
ENSMUSG00000025934	1.724	3.00E-07	Gsta3
ENSMUSG00000007682	1.7253	9.83E-06	Dio2
ENSMUSG00000030313	1.7263	0.007686	Dennd5b
ENSMUSG00000036523	1.7316	0.000119	Greb1
ENSMUSG00000065037	1.739	5.05E-05	Rn7sk
ENSMUSG00000050621	1.7395	0.001338	Rps27rt
ENSMUSG00000039081	1.7403	0.00447	Zfp503
ENSMUSG00000041426	1.758	0.001262	Hibch
ENSMUSG00000008540	1.765	6.65E-26	Mgst1
ENSMUSG00000064348	1.7704	1.95E-14	mt-Tn
ENSMUSG00000090877	1.7807	6.77E-18	Hspa1b
ENSMUSG00000086993	1.7838	0.049413	Rsf1os2

ENSMUSG00000064369	1.7896	0.044635	mt-Te
ENSMUSG00000031379	1.7907	6.28E-05	Pir
ENSMUSG00000045312	1.794	2.00E-24	Lhfpl2
ENSMUSG00000058427	1.7976	4.39E-25	Cxcl2
ENSMUSG00000041831	1.8083	3.22E-06	Sytl3
ENSMUSG00000086199	1.8118	0.000466	Bcas3os1
ENSMUSG00000032060	1.8172	5.32E-09	Cryab
ENSMUSG00000031647	1.822	1.05E-08	Mfap3l
ENSMUSG00000029657	1.8228	7.73E-62	Hsph1
ENSMUSG00000080921	1.8304	1.53E-17	Rpl38-ps2
ENSMUSG00000069270	1.8308	0.003763	Hist1h2ac
ENSMUSG00000037712	1.835	1.42E-05	Fermt2
ENSMUSG00000008734	1.8376	0.000135	Gprc5b
ENSMUSG00000074634	1.8398	1.49E-08	Tmem267
ENSMUSG00000029380	1.8426	2.31E-44	Cxcl1
ENSMUSG00000001948	1.8435	3.44E-05	Spa17
ENSMUSG00000064364	1.8481	0.001671	mt-Th
ENSMUSG00000060923	1.8519	0.032433	Acyp2
ENSMUSG00000064366	1.8549	2.07E-05	mt-Tl2
ENSMUSG00000041620	1.8574	9.82E-06	Mmp1b
ENSMUSG00000005413	1.8787	8.16E-61	Hmox1
ENSMUSG00000029819	1.8895	3.43E-05	Npy
ENSMUSG00000048388	1.8911	0.000354	Fam171b
ENSMUSG00000046080	1.8929	0.000504	Clec9a
ENSMUSG00000067704	1.8966	0.013533	Wfdc13
ENSMUSG00000064337	1.9005	5.71E-07	mt-Rnr1
ENSMUSG00000037759	1.9025	2.52E-19	Ptger2
ENSMUSG00000085939	1.9031	7.71E-30	Cd63-ps
ENSMUSG00000070883	1.9121	1.61E-10	Ccdc173
ENSMUSG00000020651	1.9122	0.04077	Slc26a4
ENSMUSG00000000402	1.9263	0.002737	Egfl6
ENSMUSG00000030208	1.9383	2.68E-31	Emp1
ENSMUSG00000030144	1.9442	8.82E-34	Clec4d
ENSMUSG00000035299	1.9518	4.59E-28	Mid1
ENSMUSG00000062582	1.9618	0.037567	Rpl30-ps8
ENSMUSG00000041324	1.975	3.77E-08	Inhba
ENSMUSG00000099398	1.9785	4.38E-12	Ms4a14
ENSMUSG00000093668	1.9806	3.04E-05	Pou5f2
ENSMUSG00000005148	1.9811	2.93E-07	Klf5
ENSMUSG00000091625	1.9834	3.52E-15	Lsm5
ENSMUSG00000028238	1.9856	3.10E-45	Atp6v0d2
ENSMUSG00000015619	1.987	0.002933	Gata3
ENSMUSG00000020460	1.9907	0.005327	Rps27a
ENSMUSG00000017002	1.995	8.26E-46	Slpi
ENSMUSG00000096351	2.0057	7.45E-10	Samd11
ENSMUSG00000095134	2.019	1.00E-12	Mid1-ps1
ENSMUSG00000019890	2.0359	2.75E-24	Nts
ENSMUSG00000064349	2.0385	2.73E-18	mt-Tc
ENSMUSG00000028691	2.0448	1.25E-47	Prdx1
ENSMUSG00000036136	2.0459	2.16E-13	Fam110c
ENSMUSG00000011008	2.0476	1.05E-53	Mcoln2
ENSMUSG00000081968	2.0502	4.77E-05	Rpl23a-ps2
ENSMUSG00000027239	2.0605	0.023999	Mdk

ENSMUSG00000031438	2.0616	3.63E-24	Rnf128
ENSMUSG00000003849	2.0625	2.09E-40	Nqo1
ENSMUSG00000050440	2.0631	1.46E-32	Hamp
ENSMUSG00000048022	2.0649	0.000841	Tmem229a
ENSMUSG00000020641	2.077	6.07E-36	Rsad2
ENSMUSG00000026604	2.0784	0.005392	Ptpn14
ENSMUSG00000031936	2.0844	0.000151	Hephl1
ENSMUSG00000032350	2.0887	4.87E-66	Gclc
ENSMUSG00000021643	2.0966	2.54E-12	Serf1
ENSMUSG00000027208	2.1004	0.001102	Fgf7
ENSMUSG00000040732	2.1094	0.048008	Erg
ENSMUSG00000070345	2.1097	0.021788	Hsf5
ENSMUSG00000004151	2.1352	0.000596	Etv1
ENSMUSG00000041020	2.136	0.001051	Map7d2
ENSMUSG00000044164	2.1438	0.027815	Rnf182
ENSMUSG00000099773	2.1448	3.46E-05	Hmgb1-rs16
ENSMUSG00000054252	2.1486	0.000818	Fgfr3
ENSMUSG00000064365	2.1492	2.09E-05	mt-Ts2
ENSMUSG00000064340	2.1619	2.10E-09	mt-Tl1
ENSMUSG00000074695	2.1889	2.98E-05	Il22
ENSMUSG00000024672	2.1908	2.20E-21	Ms4a7
ENSMUSG00000020205	2.1977	2.34E-24	Phlda1
ENSMUSG00000020913	2.2157	1.47E-11	Krt24
ENSMUSG00000020620	2.2222	1.03E-07	Abca8b
ENSMUSG00000028583	2.2477	2.67E-26	Pdpn
ENSMUSG00000038224	2.2566	0.022797	Serpinf2
ENSMUSG00000028780	2.2592	7.80E-05	Sema3c
ENSMUSG00000064361	2.2726	0.006671	mt-Tr
ENSMUSG00000074743	2.2738	0.00366	Thbd
ENSMUSG00000064339	2.2815	4.66E-07	mt-Rnr2
ENSMUSG00000037621	2.2982	0.035224	Atoh8
ENSMUSG00000036853	2.3013	0.013922	Mcoln3
ENSMUSG00000105741	2.3208	1.40E-14	C79130
ENSMUSG00000092695	2.3229	0.000244	Mir3092
ENSMUSG00000073805	2.334	3.04E-10	Fam196a
ENSMUSG00000026012	2.3485	2.59E-15	Cd28
ENSMUSG00000049929	2.3679	0.045823	Lpar4
ENSMUSG00000052861	2.393	0.001165	Dnah6
ENSMUSG00000085415	2.4198	0.000416	Selenok-ps1
ENSMUSG00000053965	2.4252	2.56E-11	Pde5a
ENSMUSG00000032348	2.4785	3.08E-08	Gsta4
ENSMUSG00000028597	2.4865	0.04703	Gpx7
ENSMUSG00000026235	2.5022	0.001992	Epha4
ENSMUSG00000029231	2.5168	0.04862	Pdgfra
ENSMUSG00000060509	2.5345	8.76E-20	Xcr1
ENSMUSG00000051228	2.5359	0.02803	Nyx
ENSMUSG00000042246	2.5401	0.014006	Tmc7
ENSMUSG00000041323	2.5437	0.000188	Ak7
ENSMUSG00000065999	2.5623	0.01507	Zfp985
ENSMUSG00000033149	2.5647	0.014811	Phldb2
ENSMUSG00000074553	2.5779	0.010692	Eif1-ps1
ENSMUSG00000050953	2.5797	1.51E-25	Gja1
ENSMUSG00000049926	2.5988	0.009749	Olfr921

ENSMUSG00000027358	2.6082	2.82E-05	Bmp2
ENSMUSG00000082532	2.6207	0.010076	Rpl31-ps6
ENSMUSG00000014704	2.6728	0.003185	Hoxa2
ENSMUSG00000064350	2.7046	3.69E-13	mt-Ty
ENSMUSG00000046447	2.7261	4.27E-29	Camk2n1
ENSMUSG00000081058	2.7329	0.008622	Hist2h3c2
ENSMUSG00000025383	2.7362	2.58E-15	Il23a
ENSMUSG00000030500	2.7512	0.004787	Slc17a6
ENSMUSG00000071342	2.7657	0.005129	Lsmem1
ENSMUSG00000029371	2.7874	9.35E-41	Cxcl5
ENSMUSG00000057933	2.8128	6.01E-11	Gsta2
ENSMUSG00000059108	2.8756	0.004158	Ifitm6
ENSMUSG00000042895	2.9156	0.017874	Abra
ENSMUSG00000031760	2.9511	8.50E-14	Mt3
ENSMUSG00000027261	2.9832	0.000313	Hao1
ENSMUSG00000098879	3.1072	0.014603	Mir7675
ENSMUSG00000070323	3.2298	0.004461	Mmp27
ENSMUSG00000025993	3.2889	8.12E-66	Slc40a1
ENSMUSG00000024600	3.3076	0.002687	Slc27a6
ENSMUSG00000056501	3.3077	2.61E-13	Cebpb
ENSMUSG00000085201	3.3364	0.00012	Nr6a1os
ENSMUSG00000038415	3.3493	0.000729	Foxq1
ENSMUSG00000019966	3.4388	9.18E-09	Kitl
ENSMUSG00000066071	3.4639	4.94E-09	Cyp4a12a
ENSMUSG00000021388	3.5333	0.004249	Aspn
ENSMUSG00000029189	3.5428	2.83E-06	Sel1l3
ENSMUSG00000026205	3.5469	0.00293	Slc23a3
ENSMUSG00000078597	3.5711	0.016526	Cyp4a12b
ENSMUSG00000109764	3.5958	0.00075	Klkb1
ENSMUSG00000021730	3.6039	0.049149	Hcn1
ENSMUSG00000078190	3.6579	0.012695	Dnm3os
ENSMUSG00000021879	3.8315	0.025882	Dnah12
ENSMUSG00000021186	3.9823	6.57E-05	Fbln5
ENSMUSG00000026765	4.2741	0.035451	Lypd6b
ENSMUSG00000031802	4.3007	0.000593	Phxr4
ENSMUSG00000064347	4.4057	0.025417	mt-Ta
ENSMUSG00000019230	4.5521	0.018546	Lhx9
ENSMUSG00000097107	4.7249	0.04191	Platr6
ENSMUSG00000032726	4.7634	0.008778	Bmp8a
ENSMUSG00000037683	4.7686	0.034732	Armc3
ENSMUSG00000021010	4.7691	0.038239	Npas3
ENSMUSG00000117220	4.8125	0.039959	CT025671.1
ENSMUSG00000030069	4.8711	0.026457	Prok2
ENSMUSG00000055489	4.8712	0.028788	Ano5
ENSMUSG00000029706	4.8714	0.034303	Pax4
ENSMUSG00000017204	4.9301	0.027633	Gsdma
ENSMUSG00000064338	5.0215	0.017585	mt-Tv
ENSMUSG00000041247	5.1988	0.003659	Lamp3
ENSMUSG00000098181	5.2037	0.010413	Rps12-ps24
ENSMUSG00000059991	5.2105	0.002348	Nptx2
ENSMUSG00000090175	5.4538	0.000698	Ugt1a9
ENSMUSG00000038567	7.9635	8.28E-09	Cyp24a1

Table S2. The primers for relative quantitative PCR were listed

Gene	Forward primer	Reverse primer
mice pxr	5’-gacctgcctattgaggacca-3’	5’-ttctggaagccaccattagg-3’
mice α5β1	5’-agcgactggaatcctcaaga-3’	5’-tgctgagtcctgtcaccttg-3’
mice gr	5’-aggccgctcagtgttttcta-3’	5’-tacagcttccacacgtcagc-3’
mice tgr5	5’-ttctctctgtccgcgtgttg-3’	5’-ggtgctgcccaatgagatga-3’
mice vdr	5’-gatgaggaggtgcagcgtaa-3’	5’-gtcgtaggtcttgtggtggg-3’
mice fxr	5’-aggggatgagctgtgtgttg -3’	5’-acactggatttcagttaacaaacct-3’
mice car	5’-ggaggaccagatctcccttc-3’	5’-ctcgtactggaaccctgcat-3’
mice GAPDH	5’-acccagaagactgtggatgg-3’	5’-cacattgggggtaggaacac-3’
mice IL-17	5’-ctcaaccgttccacgtcaccct-3’	5’-ccagctttccctccgcatt-3’
mice IFN-γ	5’-tcaagtggcatagatgtggaagaa-3’	5’-tggctctgcaggattttcatg-3’
mice MCP-1	5’-ttaaaaacctggatcggaaccaa-3’	5’-gcattagcttcagatttacgggt-3’
mice TNF-α	5’-accctggtatgagcccatatac-3’	5’-acacccattcccttcacagag-3’
mice T-bet	5’-ccagtatcctgttcccagcc-3’	5’-cataactgtgttcccgaggtgtc-3’
mice RORC	5’-ccgctgagagggcttcac-3’	5’-tgcaggagtaggccacattaca-3’
mice MCP-1	5’-ttaaaaacctggatcggaaccaa-3’	5’-gcattagcttcagatttacgggt-3’
mice TNF-α	5’-accctggtatgagcccatatac-3’	5’-acacccattcccttcacagag-3’
mice IL-6	5’-gaggataccactcccaacagacc-3’	5’- aagtgcatcatcgttgttcataca-3’
human β-actin	5’-gagaaaatctggcaccacacc-3’	5’-ggatagcacagcctggatagcaa-3’
human TGR5	5′-tctagtcggtttggtccctt-3′	5′-tctagtcggtttggtccctt-3′
